# Valve involvement in infective endocarditis among intravenous drug users: a systematic review and meta-analysis

**DOI:** 10.1186/s12879-026-13284-9

**Published:** 2026-05-12

**Authors:** Minh H. N. Le, Hung H. Phan, Vien T. Truong, Minh P. Tang, Phat K. Nguyen, An Thuy Vo, Loc T. Vu, Duy Chung, Chi N. Duong, Quoc Bui, Han H. Huynh, Trang D. T. Le, Hoai H. Le, Hien Q. Kha, Nhi H. H. Le, Dai Q. Phan, Dang Nguyen, Thach Nguyen, Tuan Vinh, Nguyen Quoc Khanh Le

**Affiliations:** 1 Section of Cardiovascular Medicine, Department of Internal Medicine, Yale School of Medicine, New Haven, CT USA; 2 International Ph.D. Program in Medicine, College of Medicine, Taipei Medical University, Taipei, Taiwan; 3 AIBioMed Research Group, Taipei Medical University, Taipei, 110 Taiwan; 4 Cardiovascular Research Laboratories, Interventional Cardiology Department, Methodist Hospital, Merrillville, IN 46410 USA; 5 School of Medicine, University of Medicine and Pharmacy at Ho Chi Minh City, Ho Chi Minh City, 700000 Vietnam; 6 Department of Cardiology, The Christ Hospital Health Network, Lindner Research Center, Cincinnati, OH 45219 USA; 7 International Master Program in Medicine, College of Medicine, Taipei Medical University, Taipei, Taiwan; 8 Faculty of Pharmacy, Can Tho University of Medicine and Pharmacy, Can Tho City, 900000 Vietnam; 9 Department of Medicine, Northwest Medical Center, Tucson, AZ 85741 USA; 10 Department of Biomedical & Nutritional Sciences, University of Massachusetts Lowell, Lowell, MA 01854 USA; 11 Department of Medicine, South Texas Health System GME Consortium, College of Medicine, Texas A&M, Edinburg, TX 78539 USA; 12 International Master Program for Translational Science, College of Medical Science and Technology, Taipei Medical University, Taipei, Taiwan; 13 Master Program in Graduate Institute of Metabolism and Obesity Sciences, College of Nutrition, Taipei Medical University, Taipei, Taiwan; 14 Mien Dong Innovative Technology University, Dong Nai, 810000 Vietnam; 15 Harvard T.H. Chan School of Public Health, Harvard University, Boston, MA 02115 USA; 16 Interventional Cardiology Department, St Mary Medical Center, Hobart, IN 46432 USA; 17 Medical Sciences Division, University of Oxford, Oxford, UK; 18 In-Service Master Program in Artificial Intelligence in Medicine, College of Medicine, Taipei Medical University, Taipei, 110 Taiwan; 19 Translational Imaging Research Center, Taipei Medical University Hospital, Taipei, 110 Taiwan

**Keywords:** Infective endocarditis, Intravenous drug use, Right-sided endocarditis, Tricuspid valve, Meta-analysis

## Abstract

**Background:**

Intravenous drug use (IVDU) is a major risk factor for infective endocarditis (IE), with right-sided infective endocarditis (RSIE) most frequently observed. However, precise estimates of valve involvement and the relative risk (RR) of RSIE within the IVDU population remain unclear. This systematic review and meta-analysis aimed to quantify the prevalence of RSIE, left-sided IE (LSIE), both-sided IE, and valve-specific involvement in IVDUs while assessing the relative risk of right-sided versus left-sided IE within the IVDU population.

**Methods:**

A systematic search was conducted in PubMed, Embase, and Web of Science to identify studies reporting IE in IVDUs. Inclusion criteria followed the PICO framework, ensuring clear definitions of IVDU status and valve involvement. A random-effects model was used to calculate pooled prevalence estimates and RR ratios with 95% confidence intervals (CIs). Heterogeneity was assessed using the I² statistic, and sensitivity analyses were performed.

**Results:**

A total of 14 studies involving 1,783 patients met the inclusion criteria. The pooled prevalence of right-sided infective endocarditis (RSIE) was 59% (95% CI: 54%–65%), left-sided infective endocarditis (LSIE) was 39% (95% CI: 33%–46%), and both-sided infective endocarditis was 8% (95% CI: 6%–12%). Among individual valves, the tricuspid valve was most commonly affected (59% (95% CI: 53%–64%)), followed by the mitral valve (21% (95% CI: 18%–26%)) and the aortic valve (16% (95% CI: 12%–20%)). Pulmonic valve involvement was rare (1% (95% CI: 0%–3%)). Overall, individuals who inject drugs (IVDUs) had a 1.49-fold increased risk of developing right-sided IE compared to left-sided IE (RR = 1.49 (95% CI: 1.15–1.95), *p* = 0.008).

**Conclusion:**

This SRMA confirms the predominance of right-sided IE, particularly tricuspid valve involvement, in IVDUs, with a significantly higher risk compared to left-sided IE. The findings underscore the need for targeted screening, early intervention, and IVDU-specific management strategies in IE care. Future research should focus on regional variations, microbiological patterns, and long-term outcomes in this high-risk population.

**Clinical trial number:**

Not applicable.

**Supplementary Information:**

The online version contains supplementary material available at 10.1186/s12879-026-13284-9.

## Key points

Right-sided infective endocarditis is the most common presentation in IVDUs, with the tricuspid valve involved in over half of cases; IVDUs have a 1.49-fold higher risk of RSIE than LSIE.

Left-sided IE (LSIE) occurs in nearly 40% of IVDU cases, often involving the mitral and aortic valves, and is associated with higher rates of complications, surgeries, and mortality.

Routine transesophageal echocardiography (TEE) and comprehensive valve evaluation are essential in IVDU-IE to avoid underdiagnosing LSIE and guide timely surgical interventions.

**Supplementary Information:**

The online version contains supplementary material available at 10.1186/s12879-026-13284-9.

## Unstructured Abstract

This systematic review and meta-analysis highlights that right-sided infective endocarditis (RSIE), particularly tricuspid valve involvement, is predominant in intravenous drug users (IVDUs), with a 1.49-fold increased risk compared to left-sided disease. However, nearly 40% of cases involve left-sided valves, which are associated with more complications, surgeries, and higher mortality. These findings challenge traditional assumptions, emphasizing the need for routine transesophageal echocardiography in all suspected IVDU-associated IE and integrated care pathways. Clinicians should be vigilant for left-sided involvement to guide early intervention, reduce complications, and optimize outcomes in this high-risk population.

## Introduction

Infective endocarditis (IE) is a severe infection of the endocardial surface, often affecting one or more heart valves [1]. Despite improvements in antimicrobial therapy and cardiac surgery, IE remains associated with high morbidity and mortality worldwide [1]. Its incidence ranges from 3 to 10 cases per 100,000 person years [2, 3]. Clinical presentations may include non-specific symptoms such as fever and malaise, along with more characteristic findings such as new heart murmurs or embolic events [2]. Without prompt diagnosis and treatment, IE can lead to complications including heart failure, systemic embolism, and stroke [2, 3].

Traditionally, IE primarily occurred in older adults with prosthetic valves or structural abnormalities. However, recent epidemiologic changes show an increasing burden among younger patients with intravenous drug use (IVDU) [3]. his shift highlights the need for updated evidence to inform diagnosis, management, and prevention. Systematic reviews and meta-analyses are essential to consolidate existing studies, resolve discrepancies, and identify patterns in IVDU associated IE. The terms ‘intravenous drug use ’ and ‘people who inject drugs (PWID)’ are used interchangeably in the literature; for consistency with included studies, we use IVDU throughout this manuscript.

The incidence of IE among IVDUs has increased substantially in parallel with the opioid epidemic [4]. Right-sided IE (RSIE), especially tricuspid valve involvement, remains the most common presentation in this group​ [4]. The pathogenesis involves repeated intravenous access introducing skin flora and particulate matter into the bloodstream, causing endothelial damage and favoring vegetation on the right-sided valves. Staphylococcus aureus is the predominant pathogen and accounts for up to 70% of RSIE in IVDUs.

RSIE makes up 5–10% of all IE cases, and the tricuspid valve is involved in as many as 90% of RSIE presentations [5]. Patients often present with bacteremia, persistent fever, and pulmonary symptoms caused by septic emboli. Although RSIE is associated with better outcomes than left-sided IE (LSIE), recurrence is common, especially in patients who continue drug use [5, 6].

Recent reports show rising LSIE cases among IVDUs, affecting mainly the mitral and aortic valves [4]. LSIE has more severe outcomes, including embolic events, valve destruction, and higher mortality. Still, the frequency of LSIE among IVDUs is not consistently reported, and comparisons with non-IVDU populations vary across studies [4].

While RSIE remains dominant, contemporary cohorts estimate that 20–35% of IVDU associated IE cases involve left-sided valves [6, 7]. A Swedish registry from 2008 to 2019, with 586 IVDU-IE episodes, reported 35% LSIE [4]. LSIE in IVDUs is often multivalvular, unlike RSIE, which usually involves only the tricuspid valve. Pulmonic valve involvement remains rare.

S. aureus remains the most common causative agent in both RSIE and LSIE. However, its proportion is higher in RSIE (up to 85%) compared to LSIE (46–48%)​ [4]. Left-sided cases had a higher proportion of streptococcal and enterococcal infections relative to right-sided cases [4]. LSIE shows relatively more streptococcal and enterococcal infections. Patients with LSIE are older on average, with a mean age of 46 compared to 35 in RSIE [4]. Structural heart defects such as patent foramen ovale are more frequent in LSIE [7], suggesting that some pathogens bypass pulmonary filtration in predisposed individuals.

LSIE is associated with worse outcomes than RSIE. It has a greater risk of systemic embolism and a higher rate of surgery, often needed due to valve destruction or persistent infection [4]. Surgery is required in 40–45% of LSIE cases, compared to only 5–8% in RSIE​ [4]. Mortality is also higher: in one US center, in-hospital mortality was significantly greater in LSIE than RSIE​ [7]. In the Swedish cohort, 5-year survival was 55% for LSIE and 84% for RSIE​ [4]. LSIE has also been linked to increased stroke risk and up to fourfold higher short term mortality.

Despite numerous observational studies, evidence gaps persist. Many studies provide inconsistent valve-specific data, and few quantify the relative risk of RSIE versus LSIE in IVDUs. To date, no meta-analysis has pooled prevalence by valve type or compared RSIE risk between IVDUs and non-IVDUs.

### Rationale

Injection drug use is a well-established risk factor for IE, with incidence rising alongside the opioid epidemic [8]. Despite this, critical knowledge gaps remain regarding valve-specific patterns and comparative epidemiology of IE in intravenous drug users (IVDUs). Many studies exclude IVDU populations or provide inconsistent data, leaving important aspects of IVDU-related IE poorly characterized [8].

Valve-specific involvement: While traditional teaching emphasizes RSIE, particularly tricuspid valve disease, recent data suggest that over 40% of IVDU-associated IE cases involve left-sided valves (aortic or mitral) [8]. For example, contemporary data show > 40% of IVDU-associated IE cases involve left-sided valves (aortic or mitral), challenging the notion that this population presents almost exclusively with tricuspid disease [8]. These discrepancies indicate heterogeneous reporting and challenge the notion that IE in IVDUs is confined to the right heart.

Lack of pooled prevalence estimates: No prior meta-analysis has synthesized the prevalence of RSIE versus LSIE in IVDUs. Individual studies vary substantially some reporting predominantly right-sided IE, others showing near equal distribution or higher left-sided involvement [8]. Without pooled estimates, the true distribution remains unclear, making generalization across settings difficult.

No prior meta-analysis of relative risk: Although it is widely accepted that IVDUs have a higher tendency for RSIE due to venous exposure to pathogens, no previous meta-analysis has quantified this relative risk compared to non-IVDUs​ [8]. The American Heart Association has noted this pattern, yet existing reviews stop short of presenting formal estimates [8].

In summary, more precise estimates of valve-specific involvement and relative risk are needed to inform diagnosis, triage, and management. This systematic review and meta-analysis aims to quantify the prevalence of RSIE, LSIE, and both-sided IE among IVDUs; characterize valve-specific patterns; and estimate the relative likelihood of right-sided versus left-sided IE within individuals who inject drugs. These findings will provide actionable insights to clinicians, researchers, and policymakers addressing the burden of IE in this growing, high risk population.

## Methods

### Protocol registration

This systematic review and meta-analysis is designed to quantify the prevalence of valve-specific infective endocarditis (IE) among intravenous drug users (IVDUs) and assess the relative risks of right-sided versus left-sided IE. The study protocol was prospectively registered in PROSPERO (Registration number: CRD420251010004) and followed PRISMA 2020 guidelines [9], ensuring that all objectives, search strategies, and planned analyses were pre-specified [Supplementary File 1].

### Literature search strategy

A comprehensive search was executed in PubMed/MEDLINE, Embase (via Ovid and Embase.com), Web of Science, and Scopus. The search strategy combined controlled vocabulary (e.g., MeSH and Emtree terms) with free-text keywords. For instance, the PubMed search string was: (“infective endocarditis” OR “endocarditis”) AND (“intravenous drug use” OR “IV drug use” OR “injection drug use” OR “substance abuse”) AND (“prevalence” OR “epidemiology” OR “incidence”). Database-specific strategies were developed, and complete search details (filters, search dates, and retrieval numbers) are provided in the [Supplementary File 2].

The primary search window was established between January 2021 and December 2025 to prioritize contemporary cohorts that reflect the current phase of the global opioid epidemic and the modern echocardiographic era. This period was intentionally selected to capture studies that utilize current diagnostic standards—including more frequent use of transesophageal echocardiography (TEE)—which have significantly improved the detection of left-sided and multivalvular involvement compared to older studies. To ensure that foundational evidence and historical trends were not overlooked, we supplemented our electronic database search with extensive citation chaining (‘snowballing’) from reference lists of relevant reviews and manual searches, allowing for the inclusion of key earlier works that met our clinical criteria.

### Eligibility criteria

Studies were eligible if they enrolled hospitalized adults (≥ 18 years) diagnosed with IE associated with IVDU. Only studies reporting echocardiographic findings (via transthoracic [3] or transesophageal (TEE) echocardiography) specifying valve involvement (right-sided, left-sided, or both-sided) were included. Studies were eligible if infective endocarditis was diagnosed using modified Duke criteria, definite IE definitions, or clearly stated clinical diagnostic frameworks supported by echocardiographic findings. We extracted information on whether studies included only “definite” IE cases or also included “possible” IE according to Duke criteria. Studies relying solely on administrative coding without clinical or echocardiographic confirmation were excluded. Articles had to be published in English in peer-reviewed journals, and both observational and randomized study designs were considered-although the majority of our included studies are observational.

Exclusion criteria comprised case reports, narrative reviews, editorials, conference abstracts without full text, and studies lacking explicit echocardiographic or left heart data. Studies that did not clearly define IVDU status or used only a historical record of drug use (rather than evidence of recent use) were excluded to ensure that the risk of infection could be directly linked to recent intravenous drug exposure [Supplementary File 3].

### Study selection process

All identified records were imported into EndNote (version X9) for deduplication. Following removal of duplicates, the remaining references were exported to Rayyan.ai for blinded title and abstract screening. Two independent reviewers [M.H.N.L., H.H.P.] screened the studies using pre-established inclusion criteria. Upon completion of this phase, blinding was lifted, and any discrepancies were resolved through consensus or consultation with a third reviewer [V.T.T.], with reference to the predefined criteria. Studies deemed potentially eligible were retrieved in full text for further evaluation. The overall selection process was systematically tracked and illustrated using a PRISMA flow diagram.

### Data collection and extraction

Data extraction was performed independently by two reviewers [M.P.T., P.K.N.] using a standardized extraction form developed in accordance with our “General-rules-for-extraction” document. The form was designed by the authors [M.H.N.L., H.H.P.] to capture detailed study identifiers (such as Reviewer, ID, Year, Title, Author, Last name of first author, and study name), final verdict decisions and reasons for exclusion, as well as key study descriptors and outcomes. Specific variables extracted include hospital and demographic characteristics (e.g., age, sex, race), cardiovascular comorbidities, structural cardiac abnormalities, substance use details, bacteriology profiles, Duke criteria-based variables, echocardiographic findings, valve involvement specifics, and clinical outcomes (including surgical interventions, complications, follow-up duration, and survival outcomes). Valve surgery was defined as surgical valve repair or valve replacement performed for infective endocarditis during the index hospitalization or during reported follow-up, as described in each study. Indications for surgery were not uniformly specified across studies and were extracted as reported.

Where studies reported medians and interquartile ranges instead of means and standard deviations, conversion was performed using validated tools (https://meta-converter.com/conversions/mean-sd-iqr). All extracted data were entered into a master spreadsheet, and quality control measures (including manual checks by assigned reviewers on 20 March 2025) ensured data accuracy.

### Risk of bias and quality assessment

Risk of bias for each included study was assessed using the ROBINS-E tool [10] and its updated version (ROBINS-E new), both tailored for evaluating nonrandomized studies of exposures. These tools assess multiple domains, including selection bias, exposure measurement, confounding, outcome assessment, and reporting bias. Two independent reviewers [T.D.T.L., H.H.L.] performed these assessments, and discrepancies were resolved by consensus or by consulting a third reviewer [V.T.T.].

Studies were categorized as low, moderate, or high risk of bias based on predefined criteria. Detailed risk of bias tables are available in the [Appendix Table 5], and sensitivity analyses were planned to determine the impact of high risk studies on the overall pooled estimates. This rigorous quality assessment process ensures that the meta-analysis relies on methodologically sound evidence.

### Data synthesis and statistical analysis

Pooled estimates for the prevalence of right-sided, left-sided, and both-sided infective endocarditis were computed using a random-effects meta-analysis (DerSimonian and Laird method) [11] to account for inter-study heterogeneity, performed by [M.H.N.L., V.T.T.]. For studies reporting relative risk (RR) comparing RSIE in IVDUs to non-IVDUs, log-transformation of RR values was applied, and corresponding 95% confidence intervals were calculated. Statistical significance was set at *p* < 0.05.

Heterogeneity was assessed using the I² statistic with thresholds of 25%, 50%, and 75% signifying low, moderate, and high heterogeneity, respectively and Cochran’s Q test [12] was employed. Forest plots were generated for visual representation of individual study estimates and pooled results, while funnel plots and Egger’s regression tests [13] were conducted to evaluate publication bias. All statistical analyses were conducted using review manager (RevMan version X) and R.

Sensitivity and Influence Diagnostics To ensure the robustness of the pooled estimates, influence diagnostics were performed for each outcome using Cook’s distance, dffits, Studentized residuals, and changes in tau² and Cochran’s Q upon study deletion [14]. Baujat plots were generated to visualize the contribution of individual studies to overall heterogeneity [15]. Furthermore, a leave-one-out sensitivity analysis was conducted for all primary outcomes to determine if any single study significantly altered the pooled prevalence. In cases where significant outliers or highly influential studies were identified, a post-sensitivity analysis was performed to provide adjusted pooled estimates. Funnel plots for proportions were constructed using logit-transformed values to evaluate publication bias.

### Assessment of publication bias

Publication bias was investigated through visual inspection of funnel plots, plotting effect sizes against standard errors [M.H.N.L.]. Egger’s regression test was used to statistically evaluate funnel plot asymmetry, with *p* < 0.05 indicating potential bias. The impact of small-study effects on the pooled estimates was examined, and any necessary adjustments are documented.

### Data sharing statement

The data supporting the findings of this study are available from the corresponding author upon reasonable request. All extracted datasets, analysis scripts, and supplementary materials used for meta-analyses are stored in a secured institutional repository and can be shared with qualified researchers for academic and non-commercial purposes following approval of a data use agreement.

## Results

### Study selection and characteristics

**Fig. 1:**
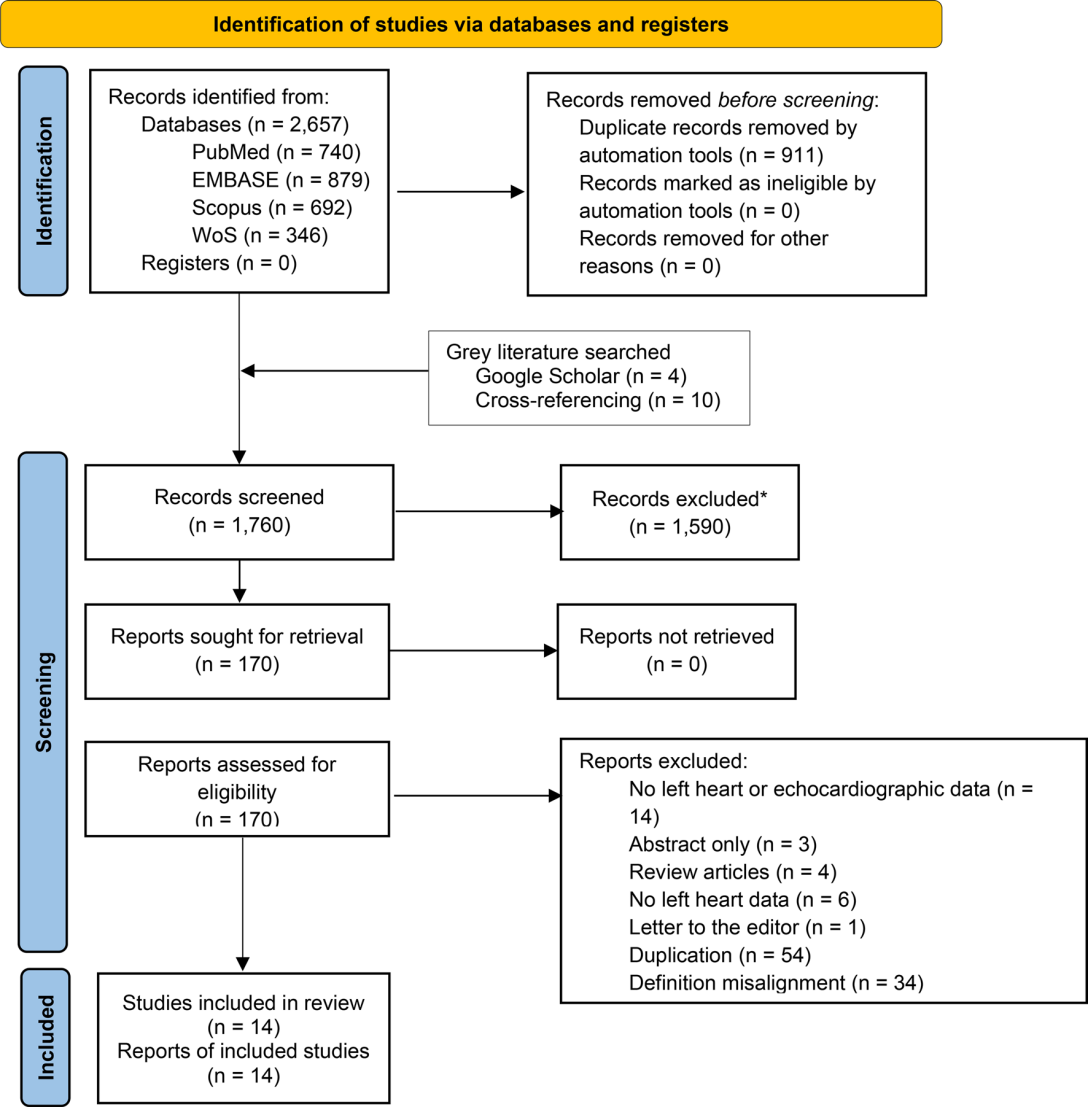
PRISMA flowchart diagram for study selection. * Title and abstracts were screened using Rayyan.ai, a semi-automated tool for abstract screening. All exclusion decision were made by human

A total of 2,657 records were identified through database searches, with 911 duplicates removed. After screening 1,760 records, 170 full-text articles were reviewed for eligibility. Of these, 156 were excluded for reasons such as missing echocardiographic data, abstract-only format, or misaligned definitions. Ultimately, 14 studies met the inclusion criteria and were included in the systematic review and meta-analysis. The selection process is detailed in the PRISMA flowchart (Fig. 1; Appendix Table 1).

Across the 14 included studies [16–29], a total of 1,783 patients with intravenous drug use–associated infective endocarditis (IVDU-IE) were analyzed. The majority were male (61.8%), with a mean age of 36.2 years (SD ± 11.86), reflecting a relatively young demographic. The crude all-cause mortality rate was 21.4%, and follow-up duration, when reported, was generally around 12 months but often incomplete. Transthoracic echocardiography (TTE) was utilized in 90.2% of patients, while transesophageal echocardiography (TEE) was performed in 53.2%. HIV co-infection was reported in 18.08% of cases, with notable rates of hepatitis B (6.45%) and hepatitis C (58.70%), highlighting a high burden of bloodborne viral comorbidities. Culture-negative infections accounted for 9.7% of cases. Among identified pathogens, *Enterococcus faecalis* (3.93%), *Pseudomonas spp.* (1.29%), and polymicrobial infections (5.16%) were most commonly reported, while *HACEK* organisms were rare (0.11%). Race and comorbidity data were inconsistently reported across studies.

### Risk of bias

Risk of bias was assessed using the ROBINS-E tool across all 14 included studies. The majority of studies exhibited some concerns in at least one domain, with only three studies (Asgeirsson 2016, Damlin 2021, and Hilbig 2020) judged to have an overall low risk of bias. Four studies (Jain 2008, Low 2020, Meel 2018, and Syed 2021) were assessed as having a high overall risk of bias, primarily due to serious limitations in domains such as confounding, selection of participants, and missing data. The remaining studies were categorized as having moderate risk, reflecting uncertainty in areas such as exposure measurement and control of confounding variables.

At the domain level, bias due to confounding was identified as a common issue, with only a minority of studies achieving low risk ratings. Measurement of the exposure and selection of participants were generally well handled, although a few studies demonstrated high risk ratings in these areas. Importantly, all studies were considered to have low risk of bias related to post exposure interventions. In contrast, missing data emerged as a consistent concern across nearly all studies, contributing significantly to the overall risk profiles. Outcome measurement and selection of reported results were mostly deemed reliable, though some studies had minor issues. These findings highlight variability in methodological quality and underscore the importance of sensitivity analyses. To account for this, we conducted additional analyses excluding high risk studies, confirming the robustness of the main meta-analytic findings [Supplementary Fig. 1].

Among the 14 included studies, 10 employed the Modified Duke criteria [17–19,21−24,27,29,30] and 4 applied the original Duke criteria for IE diagnosis [19, 24, 25, 27]. Of the 10 studies using Modified Duke criteria, 7 restricted inclusion to definite IE cases only, while the remaining 3 also incorporated possible IE cases. Among the 4 studies using the original Duke criteria, 1 restricted inclusion to definite cases only, while the remaining 3 also included possible IE cases. Variation in reporting of diagnostic classification may contribute to between-study heterogeneity, particularly in estimates of valve involvement and complication rates. Detailed study-level diagnostic definitions are provided in [Appendix Table 2].

### Tricuspid valve involvement

**Fig. 2:**
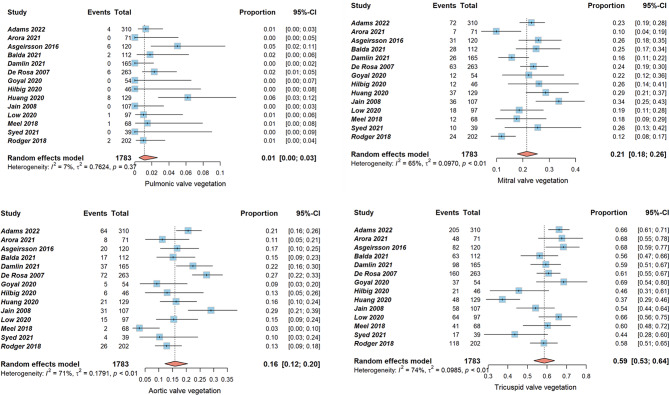
Forest plot: Pooled prevalence of heart valves involvement in IVDU-associated IE

Tricuspid valve involvement was the most prevalent in IVDU-associated IE, with a pooled prevalence of 59% (95% CI: 53%-64%) across 14 studies [16–29]. Despite moderate variability and substantial heterogeneity (I² = 74%), the estimate remained robust. The funnel plot showed no significant asymmetry, and Egger’s test (*p* = 0.120) indicated low risk of publication bias. These findings affirm the tricuspid valve’s dominant role due to its direct exposure to injected pathogens (Fig. 2; Appendix Fig. 5).

### Pulmonic valve involvement

Pulmonic valve involvement in IVDU-associated infective endocarditis was exceedingly rare, with a pooled prevalence of 1% (95% CI: 0%-3%) and low heterogeneity (I² = 7%). Most studies reported few or no cases, reflecting strong consistency. The funnel plot was symmetric; though interpretation is limited due to the low event rate. These findings affirm that pulmonic valve involvement is negligible in this population (Fig. 2; Appendix Fig. 7).

### Mitral valve involvement

Mitral valve involvement was observed in 21% (95% CI: 18%-26%) of IVDU-associated IE cases across 14 studies, with moderate heterogeneity (I² = 65%). Study estimates varied, but the funnel plot appeared symmetric and Egger’s test (*p* = 0.020) showed minor bias [16–29]. These results emphasize that mitral valve disease, though less common than tricuspid, remains clinically significant due to its link with embolic events and surgical need (Fig. 2; Appendix Fig. 9).

### Aortic valve involvement

Aortic valve involvement occurred in 16% (95% CI: 12%–20%) of IVDU, associated IE cases across 14 studies, with moderate-to-high heterogeneity (I² = 71%) [16–29]. The funnel plot showed slight asymmetry, and Egger’s test (*p* = 0.003) suggested possible small-study effects. Despite this, findings were consistent overall, underscoring the clinical relevance of aortic involvement due to its link with serious complications like acute insufficiency and systemic embolism (Fig. 2; Appendix Fig. 11).

### Left heart involvement

**Fig. 3:**
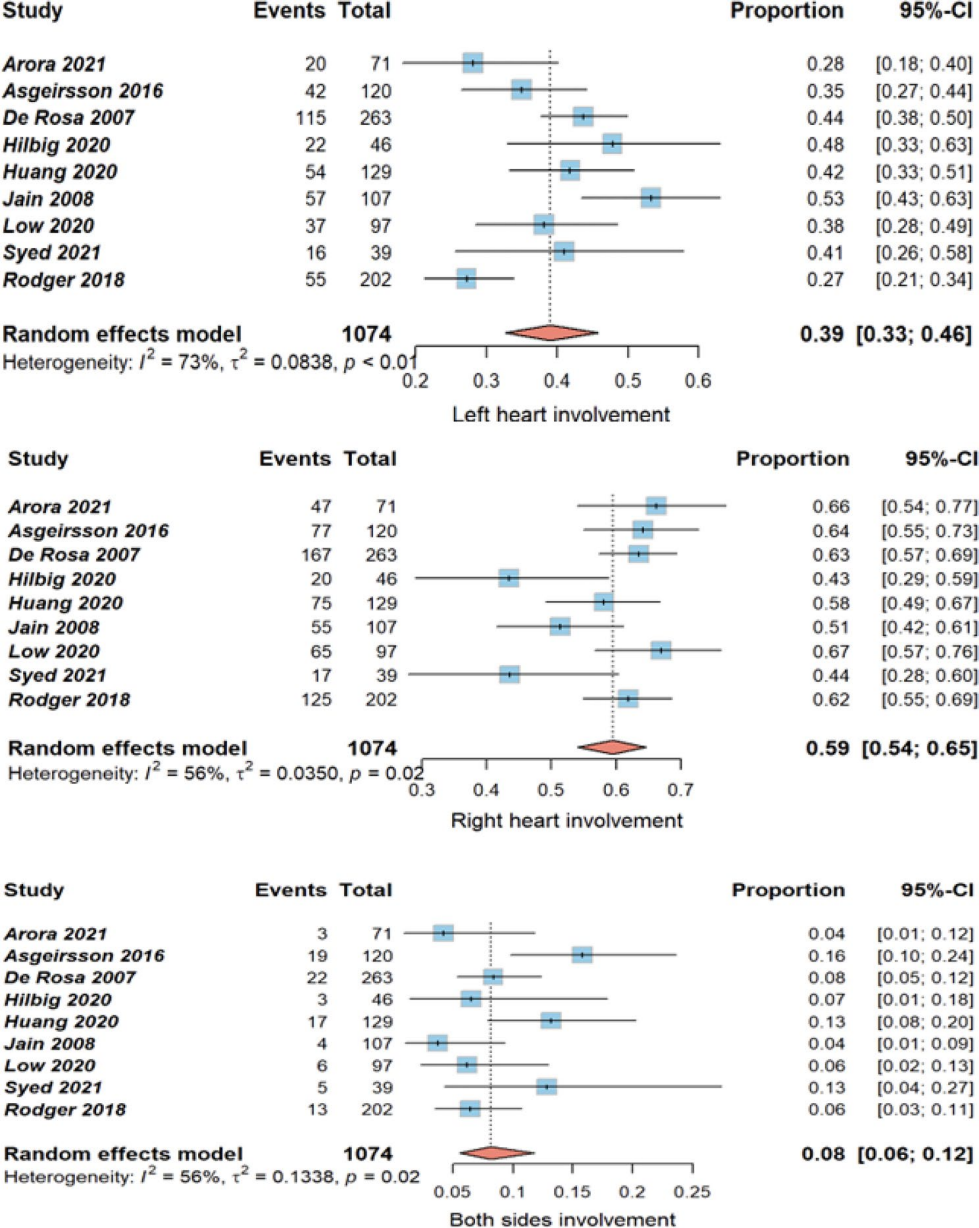
Forest plot: Pooled prevalence of heart side involvement in IVDU-associated IE

Left-sided valve involvement was present in 39% (95% CI: 33%-46%) of IVDU, associated IE cases across 8 studies, with substantial heterogeneity (I² = 73%). [17–20,25−28,30] The funnel plot appeared symmetric, and Egger’s test (*p* = 0.289) showed no significant publication bias. These results challenge the belief that IVDU-IE is mainly right-sided, underscoring the need for thorough evaluation and early intervention due to LSIE’s higher risk of complications (Fig. 3; Appendix Fig. 13).

### Right heart involvement

Right sided endocarditis was the most prevalent form in IVDU, associated IE, with a pooled prevalence of 59% (95% CI: 54%–65%) across 8 studies (I² = 56%). [17–20,25−28,30] Individual proportions ranged from 43% to 67%. The funnel plot appeared symmetrical, and Egger’s test suggested minor publication bias (*p* = 0.010). These findings confirm the predominance of right heart involvement, particularly tricuspid valve disease, in this population. However, the notable presence of left-sided involvement emphasizes the need for full echocardiographic evaluation in all IVDU-IE cases (Fig. 3; Appendix Fig. 15).

### Both sides valve involvement

Both-sided valve involvement was relatively uncommon, with a pooled prevalence of 8% (95% CI: 6%–12%) across 8 studies (I² = 56%) [16, 18, 19, 24–27, 29]. Estimates ranged from 4% to 16%, indicating moderate heterogeneity. The funnel plot showed a fairly symmetrical distribution, and no major publication bias was evident. Although rare, both-sided IE may reflect more aggressive disease or delayed diagnosis. This underscores the importance of comprehensive imaging and early intervention in IVDU patients presenting with severe or multivalvular symptoms (Fig. 3; Appendix Fig. 17).

### Right sides risk

**Fig. 4:**
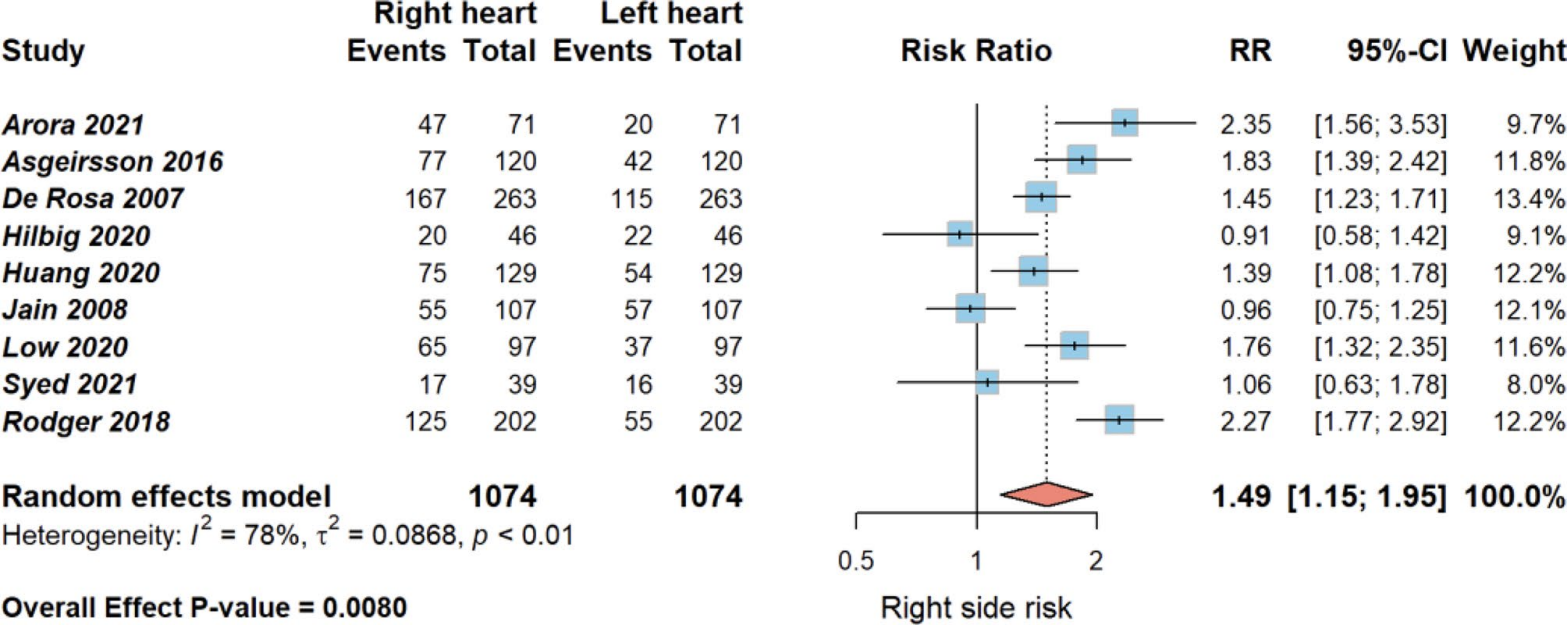
Forest plot: Relative risk of right-sided IE in IVDUs vs. left-sided IE

Pooled analysis of 8 studies showed that IVDUs had a 1.49, fold higher risk of developing right-sided IE compared to left-sided IE (RR = 1.49, 95% CI: 1.15–1.95, *p* = 0.008) [16, 18, 19, 24–27, 29]. Heterogeneity was substantial (I² = 78%), suggesting some between study variability. Despite this, the funnel plot appeared balanced, with no strong indication of publication bias. These findings quantitatively reinforce the clinical observation that right-sided endocarditis is more likely in the IVDU population, especially involving the tricuspid valve (Fig. 4; Appendix Fig. 19).

**Fig. 5:**
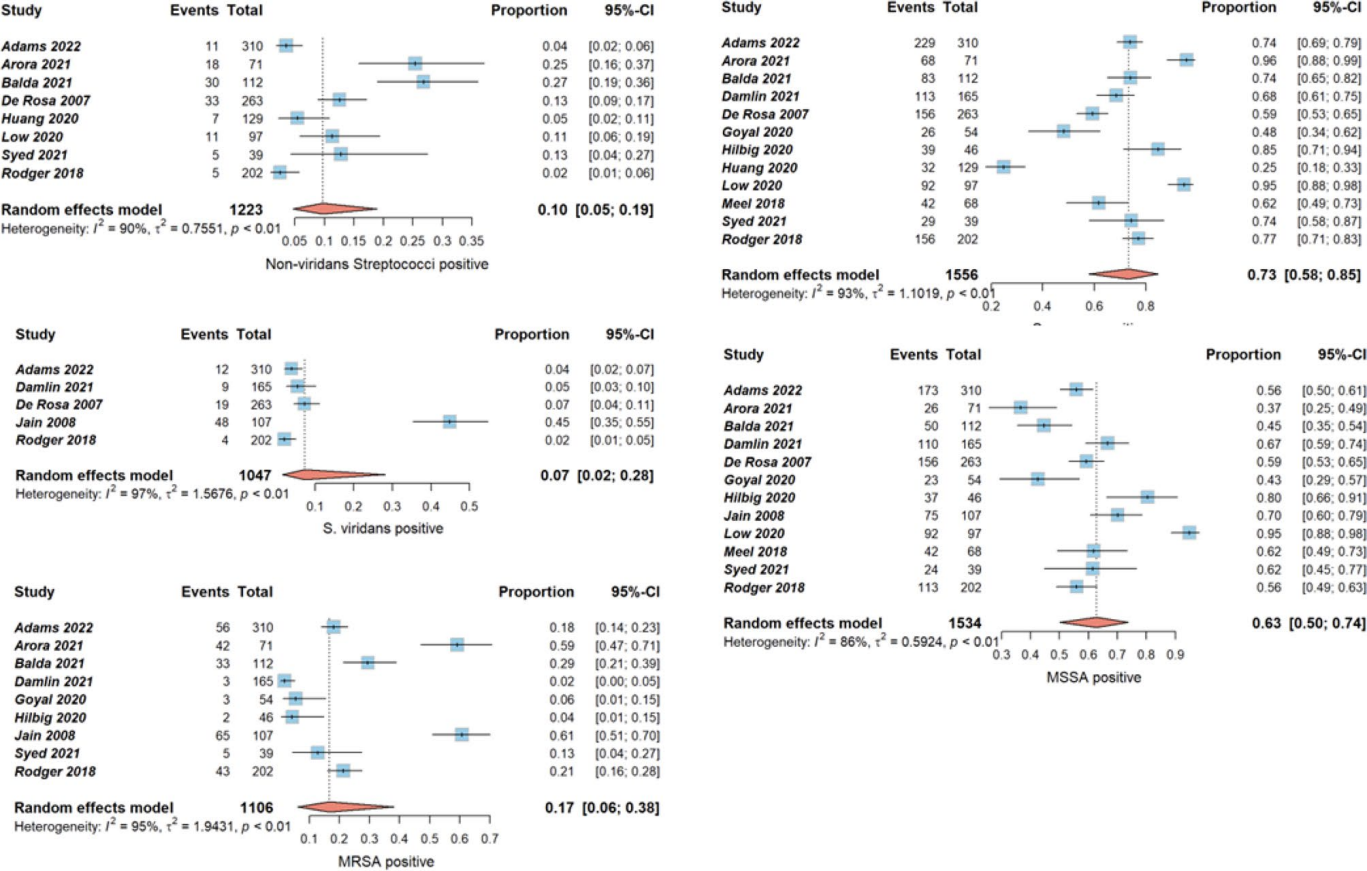
Forest plot: Micro-organism prevalence in IVDU-associated IE

### Staphylococcus aureus positive

Staphylococcus aureus was the most frequently isolated pathogen in IVDU, associated IE, with a pooled prevalence of 73% (95% CI: 58%–85%) across 12 studies (I² = 93%). [18–25,27−30] Individual proportions ranged from 25% to 96%, indicating high heterogeneity. The funnel plot showed some asymmetry, likely reflecting small study effects or variation in microbiological methods. Despite variability, these findings highlight S. aureus, especially methicillin-sensitive strains as the dominant causative agent in this population, supporting empiric coverage in initial treatment regimens (Fig. 5; Appendix Fig. 21).

### MRSA positive

The pooled prevalence of methicillin-resistant Staphylococcus aureus (MRSA) among IVDU, associated IE cases was 17% (95% CI: 6%-38%) across 9 studies (I² = 95%), indicating high heterogeneity [18–22, 25, 27–29]. Individual study estimates ranged from 2% to 50%. The funnel plot showed mild asymmetry, possibly due to small study effects or reporting variation. Despite the variability, MRSA represents a significant minority of S. aureus infections in this population, underscoring the importance of considering MRSA coverage in empiric antibiotic regimens, especially in high prevalence settings (Fig. 5; Appendix Fig. 23).

### MSSA positive

Methicillin-sensitive Staphylococcus aureus (MSSA) accounted for the majority of S. aureus infections, with a pooled prevalence of 63% (95% CI: 50%–74%) across 12 studies (I² = 86%). [18–26,28−30] Study-level estimates ranged widely (37%–76%), indicating considerable heterogeneity. The funnel plot showed slight asymmetry, possibly reflecting reporting differences. Despite this, MSSA remains the dominant subtype in IVDU-associated IE, reinforcing the importance of β-lactam coverage in initial empiric therapy when local resistance patterns permit (Fig. 5; Appendix Fig. 25).

### Non-viridans streptococci positivity

The pooled prevalence of non-viridans Streptococci in IVDU-associated IE was 10% (95% CI: 5%–19%) across 8 studies (I² = 90%), reflecting high heterogeneity [17, 18, 21, 22, 24, 26, 27, 29]. Individual estimates varied, with some studies reporting rates as high as 27%. The funnel plot showed symmetric distribution, suggesting minimal publication bias. While less common than S. aureus, these organisms still warrant consideration, particularly in left-sided infections (Fig. 5; Appendix Fig. 27).

### S. viridans positivity

The pooled prevalence of Streptococcus viridans in IVDU-related IE was 7% (95% CI: 2%–28%) across 5 studies, with very high heterogeneity (I² = 97%) [20, 21, 24, 25, 29]. Most studies reported low rates, except one outlier (Jain 2008) with a notably higher estimate. The funnel plot showed asymmetry, suggesting potential small-study effects. While uncommon, S. viridans may still contribute to IE in select cases, particularly those with underlying structural heart disease (Fig. 5; Appendix Fig. 29).

### Valve surgeries

**Fig. 6:**
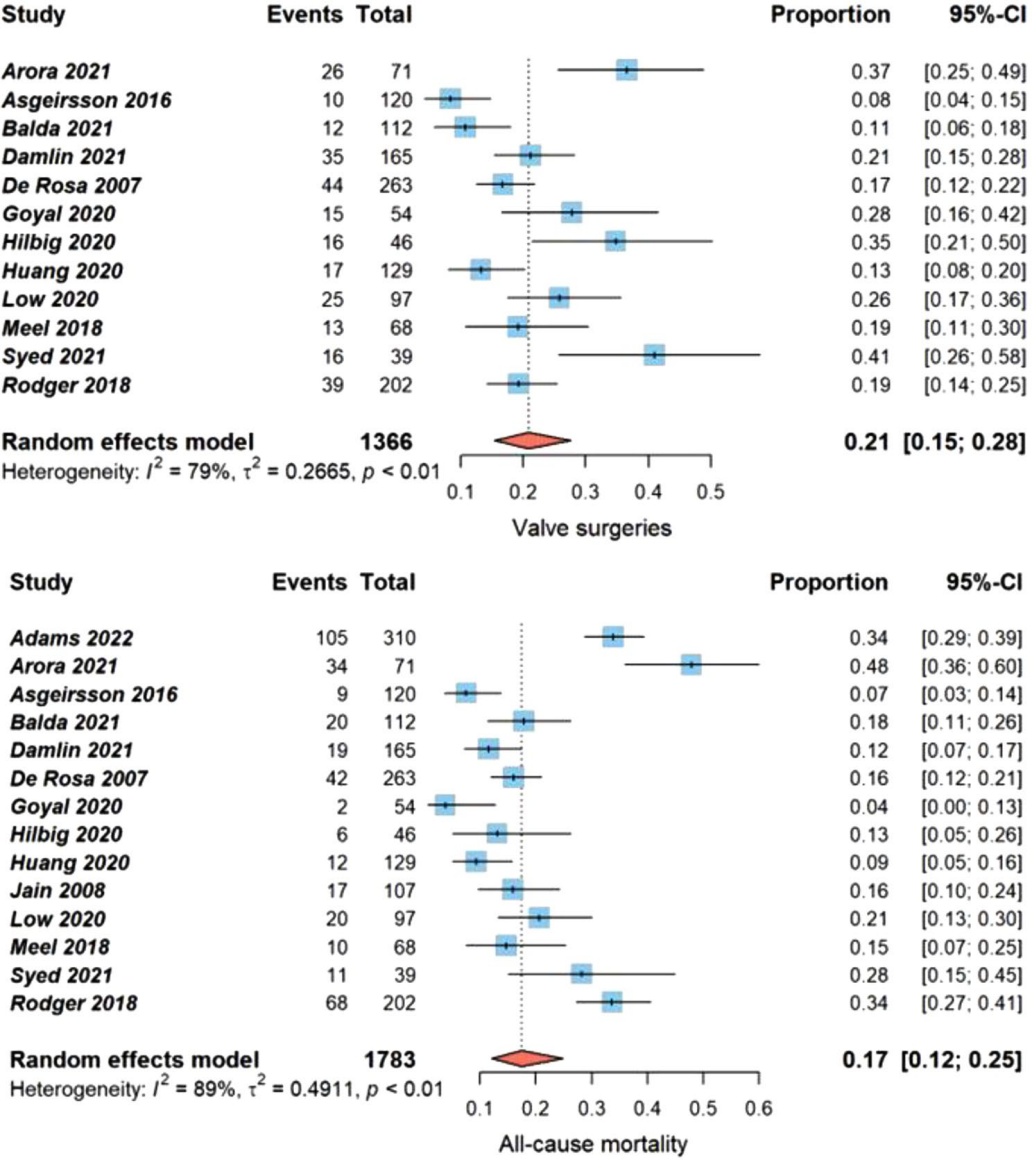
Forest plot: Valve surgery and all-cause mortality in IVDU-associated IE

The pooled prevalence of valve surgeries among IVDU-associated IE cases was 21% (95% CI: 15%–28%) across 12 studies (I² = 79%). [17–21,23−25,27–30] Surgical rates varied notably between studies (range: 8%–37%), likely reflecting differences in disease severity and access to care. The funnel plot showed good symmetry, suggesting minimal publication bias. These findings emphasize the significant surgical burden in this population, particularly in cases with left-sided involvement or complications (Fig. 6; Appendix Fig. 31).

### All cause mortality

The pooled all-cause mortality among IVDU-associated IE patients was 17% (95% CI: 12%–25%) across 14 studies (I² = 89%), indicating high heterogeneity [16–29]. Mortality rates varied widely (3%–48%), likely reflecting differences in clinical severity and treatment access. Mortality reporting varied substantially across included cohorts: Several studies reported exclusively in-hospital mortality, including Hilbig 2020, Damlin 2021 (4% in IDU-IE), Balda 2021 (12% at 90 days), Meel 2018 (14.7%), De Rosa 2007 (16%), Goyal 2020 (19.5% overall; 22.2% in IVDA), and Jain 2008 (6.3% for tricuspid valve IE, 32% for mitral valve IE). Several studies reported in-hospital mortality (or outcomes until discharge), while others reported fixed short-term mortality (e.g., 10-week or 90-day mortality) or mortality during follow-up with variable observation windows. Follow-up duration was inconsistently reported, limiting the ability to standardize mortality across studies: Asgeirsson 2016 reported both in-hospital (2.5%) and 1-year mortality (8.0%); Low 2020 reported in-hospital (16%) and 1-year follow-up outcomes; Huang 2020 reported 10-week mortality (22% for left-sided IE vs. 5% for non-left-sided IE in PWID); Syed 2021a reported 15% in-hospital mortality and 28% overall mortality at a mean follow-up of 14 months; Adams 2022 reported 33.8% all-cause mortality over a mean follow-up of 1.3 years; and Rodger 2018 reported an all-cause mortality of 33.7% at 1-year follow-up. Furthermore, the type of mortality assessed also differed: while most studies reported all-cause mortality, some specifically examined cause-specific mortality (e.g., IE-related death), and the distinction between in-hospital, short-term (e.g., 10-week, 90-day), and longer-term mortality was not uniformly standardized. The funnel plot was relatively symmetrical, with no strong evidence of publication bias. These findings underscore the significant mortality burden in this population, highlighting the need for early diagnosis and integrated care strategies (Fig. 6; Appendix Fig. 33).

Nevertheless, several consistent patterns emerged qualitatively across studies: left-sided valve involvement was repeatedly identified as a predictor of higher mortality (Low 2020, Goyal 2020, Rodger 2018, De Rosa 2007, Huang 2020); Staphylococcus aureus infection—particularly methicillin-resistant strains—was associated with increased mortality in multiple cohorts (Meel 2018, Damlin 2021, Asgeirsson 2016); and referral to addiction treatment services was associated with reduced mortality in the studies that examined this variable (Rodger 2018, Adams 2022). These findings underscore that while IVDU-associated IE carries a substantial mortality burden, the variability in follow-up and outcome ascertainment across the current literature represents a significant limitation that future prospective, multi-center studies with standardized follow-up protocols should aim to address.

### Sensitivity analysis

Sensitivity analyses confirmed the robustness of our findings across all major outcomes. Exclusion of influential studies identified via Baujat plots and influence diagnostics. For example, Huang 2020 for tricuspid involvement and Rodger 2018 for mitral and left-sided involvement did not substantially alter pooled estimates. Leave-one-out analyses consistently showed that no single study materially changed the overall proportions or effect sizes. Heterogeneity was modestly reduced in several models after removing high-impact studies, but core conclusions remained stable. Full plots and diagnostic outputs are presented in [Appendix Figs. 34 to 61].

## Discussion

### Prevalence of left-sided IE in IVDUs

Historically, LSIE in IVDU was considered uncommon. Early cohorts reported very low rates of left-sided involvement. For example, Moss et al. found only 14% of IVDU-IE cases involved left-sided valves [30]. Notably, even in that 1995–1998 series the authors observed that more recent data suggested a rising proportion of left-sided disease [30]. Our pooled analysis confirms this trend: LSIE now accounts for approximately 39% of IVDU-IE cases. This is considerably higher than the single digit proportions reported in earlier studies and reflects a clear shift over time. In our dataset, the mitral valve was involved in more than 20% of cases, and the aortic valve in approximately 17%, often with multivalvular involvement. These findings indicate that clinicians should maintain a high index of suspicion for left-sided disease even in IVDUs, rather than assuming IE will be right-sided. Consistent with our results, large contemporary cohorts have reported substantial left-sided involvement in IVDUs. For example, a recent multicenter registry found left-sided valve involvement in over half of IVDU-IE episodes [31].

Infective endocarditis in PWID has been described as predominantly right-sided. However, our pooled estimate demonstrates substantial left-sided involvement (~ 39%), suggesting a potential epidemiologic shift. Several factors may explain this pattern. First, improved diagnostic sensitivity - particularly greater use of TEE - may have increased detection of left-sided vegetations and peri-annular complications that were previously underrecognized [7, 31, 32]. Second, evolving substance use patterns, including changes in injected substances and preparation practices, may alter bacteremia dynamics and endothelial injury [33]. Third, contemporary PWID cohorts increasingly include older individuals and patients with prior healthcare exposure or underlying valvular abnormalities, which may predispose to left-sided disease [31, 34]. 

Taken together, the observed rise in LSIE likely reflects both improved diagnostic recognition and true changes in patient characteristics and exposure patterns, underscoring the importance of comprehensive left-sided evaluation in this population.

### Peri-annular complications

LSIE is associated with higher rates of destructive complications. Peri-annular complications involve extension of infection into the valve annulus and adjacent cardiac tissues (e.g. the aortic root or fibrous skeleton). These typically manifest as perivalvular abscesses, pseudoaneurysms, or fistulae [35]. The prognostic burden of peri-annular complications in left-sided IE has been further quantified in a recent meta-analysis by Emara et al., which found that aortic root abscess - the most common form of paravalvular abscess - was associated with significantly higher in-hospital mortality, late mortality, increased IE recurrence, and greater rates of multi-organ failure compared to IE without this complication [36]. Such peri-annular extensions are clinically significant: they often lead to conduction abnormalities (e.g. heart block), aortocavitary communications, and rapid hemodynamic deterioration. In prosthetic valve endocarditis, peri-annular involvement was identified in approximately 17% of cases and conferred very high mortality [37]. In our review, LSIE patients had a markedly higher incidence of these complications than those with right-sided disease. These serious complications generally necessitate urgent surgical intervention, as medical therapy alone is usually insufficient [37]. Indeed, we found 40–45% of LSIE patients required valve surgery versus < 10% of isolated tricuspid cases, reflecting both the destructive nature of left-sided infections and the difficulty of managing peri-annular pathology medically.

### Clinical implications and diagnostic practices

The high burden of left-sided disease in IVDUs has important diagnostic implications. Echocardiography is the cornerstone of IE diagnosis. TTE is commonly used first, but its sensitivity for native valve IE is estimated to be between 50 and 75% [35]. By contrast, TEE offers significantly greater sensitivity, approaching 100% for native valves [35]. TEE is especially important for detecting left-sided vegetations, small lesions, and peri-annular complications that TTE can miss. In fact, guidelines recommend early TEE in any IE case with high risk features or equivocal TTE findings [35]. Given that we found 33–41% of IVDU-IE cases involve the left heart, optimal practice would be to perform TEE routinely in suspected IVDU-IE, even if TTE is negative or limited. Our findings reinforce prior evidence (and guideline recommendations) that a negative TTE alone is insufficient to rule out left-sided involvement in this population [35].

In practice, however, echo utilization varied among the studies we reviewed. Few studies detailed standardized imaging protocols: some relied primarily on TTE for initial diagnosis, while others performed TEE only when indicated. This heterogeneity likely contributed to variation in reported left-sided IE rates. For example, retrospective chart reviews at tertiary centers generally used TEE liberally and found LSIE in approximately 30% of cases, whereas population based registries or older series (which may have used TTE alone in some patients) reported lower LSIE prevalence. Although TTE was widely performed, TEE was utilized in only approximately 53% of cases across included cohorts. Given that TEE has substantially higher sensitivity than TTE for detecting left-sided vegetations, small lesions, and peri-annular complications, incomplete TEE utilization may have resulted in underestimation of LSIE prevalence and structural complications such as abscesses, pseudoaneurysms, and fistulae. This diagnostic variability may introduce misclassification bias, whereby some cases categorized as isolated right-sided IE could in fact have had undetected left-sided involvement. Furthermore, peri-annular complications—more common in LSIE—often serve as key indications for surgery; therefore, limited TEE use may also partially explain between-study variability in reported surgical rates.

Similarly, follow up practices differed: several studies did not report routine repeat echocardiography during treatment, making it hard to assess vegetation evolution or late complications uniformly. Best practice - endorsed by expert guidelines would include baseline TEE for thorough assessment, followed by periodic re-imaging (usually by echo) if the clinical course is complicated or if new murmurs/decompensation occur. Our analysis suggests that many IVDU-IE patients may not receive this full diagnostic workup in every setting, potentially delaying recognition of serious complications.

Substantial heterogeneity across pooled estimates—particularly for Staphylococcus aureus prevalence, MRSA/MSSA distributions, valve surgery, and mortality—likely reflects real-world clinical and epidemiologic diversity rather than methodological weakness alone. The included cohorts span multiple healthcare systems and eras (e.g., Sweden: Asgeirsson 2016; Damlin 2021; Australia: Low 2020; Hilbig 2020; India: Arora 2021; Goyal 2020; South Africa: Meel 2018; Italy: De Rosa 2007; North America: Jain 2008; Huang 2020; Syed 2021a; Adams 2022; Balda 2021; Rodger 2018), where temporal shifts in opioid/injection practices, differences in harm reduction coverage, and local microbiologic ecology can materially influence pathogen distributions and resistance profiles. Similarly, heterogeneity in diagnostic pathways—especially variation in TEE use (recorded at 53.2% in our pooled cohort) and timing of repeat imaging—may affect detection of left-sided disease and peri-annular complications, thereby influencing reported complication rates, surgical indications, and downstream mortality. Finally, thresholds for surgery and access to cardiothoracic expertise vary across centers and regions, leading to meaningful between-study differences in surgery rates and outcomes. Consequently, our pooled estimates should be interpreted as average effects across heterogeneous real-world settings; this enhances external validity for broad clinical contexts but implies that point estimates may differ when applied to specific regions, time periods, or institutional care pathways.

Although age and sex were commonly reported across studies, race/ethnicity and socioeconomic indicators (such as income, education, housing stability, and insurance status) were inconsistently captured, limiting subgroup analysis by these potentially important determinants of health. Missing demographic data may constrain the generalizability of our pooled estimates, especially given the known intersection between social determinants and IE outcomes. Structural inequities—such as differential access to preventive services, harm-reduction programs, addiction treatment, and cardiothoracic care—are increasingly recognized contributors to variation in IE outcomes; for example, socioeconomic deprivation has been associated with higher in-hospital mortality and adverse events among patients hospitalized for IE in a recent large national analysis in the United States [38]. Non-clinical factors including unstable housing, limited primary care access, and stigma may also influence thresholds for surgical referral and timing of care, indirectly affecting surgery rates and mortality in IVDU. Finally, most included cohorts were conducted in high-income settings, and the applicability of our findings to low- and middle-income countries—where IVDU patterns, healthcare infrastructure, and care access differ substantially—is uncertain. Recognizing these social and structural contributors enhances interpretation of our pooled estimates and underscores the need for future studies to collect and report comprehensive demographic and socioeconomic data to better inform equitable clinical practice.

Discrepancies were also apparent in management and follow up protocols. Some included studies described aggressive surgical strategies for left-sided lesions or high risk vegetations, whereas others favored extended antibiotic therapy with surgery only for clear indications. Post-discharge follow up (to monitor for relapse or recurrent infection) was often poorly described. This variability underscores the need for standardized care pathways in IVDU-IE. For example, ensuring every patient has at least one comprehensive echocardiographic evaluation and a clear plan for follow up imaging and addiction treatment. Aligning real world practice with guideline-recommended diagnostics (including routine TEE) and structured monitoring would likely improve detection of LSIE and its complications in this high risk group, ultimately enabling timelier interventions.

Although four studies were judged to have an overall high risk of bias, sensitivity analyses excluding these studies did not materially alter the direction or statistical significance of pooled prevalence estimates or the relative risk comparing right- versus left-sided IE. High-risk ratings were primarily driven by concerns related to confounding, missing data, and incomplete reporting rather than systematic misclassification of valve involvement. While these methodological limitations may have introduced some imprecision—particularly in microbiologic proportions, surgical rates, and mortality estimates—the stability of leave-one-out and influence analyses suggests that no single high-risk study disproportionately influenced the primary findings. Therefore, although caution is warranted in interpreting absolute prevalence values, the overall pattern of predominance of right-sided disease and substantial left-sided involvement appears robust across methodological strata.

### Prognosis and medical vs. surgical management

Our findings confirm that LSIE carries a much worse prognosis than isolated right-sided disease. Left-sided vegetations have access to the systemic circulation and are far more likely to embolize (to brain, kidneys, spleen, etc.), precipitate heart failure, and cause irreversible valvular destruction [30, 35]. Peri-annular abscesses and fistulae seen predominantly in left-sided cases further drive the need for surgical intervention. In keeping with this, many of patients with left-sided IE in our cohort required valve surgery (often urgently), compared to < 10% with isolated tricuspid IE. Surgery was typically indicated for persistent bacteremia, heart failure from regurgitation, or large mobile vegetations with embolic potential. These data highlight the disproportionate burden of surgical disease in LSIE, which aligns with existing guidelines that prioritize prompt surgery for complicated left-sided infections.

Surgical indications were variably described across studies and likely reflect differences in institutional expertise, referral pathways, and local surgical thresholds. Centers with established cardiothoracic programs may report higher surgical rates compared to settings with limited surgical access. Therefore, surgical prevalence should be interpreted as descriptive rather than prescriptive. Importantly, the primary aim of this meta-analysis was to characterize valve involvement and diagnostic patterns in IVDU-associated infective endocarditis, rather than to evaluate surgical management strategies.

### Socioeconomic and behavioral barriers

As discussed in the original manuscript, non-medical factors greatly influence outcomes in IVDUs with IE. Unstable housing, ongoing substance use, and limited access to care often hamper antibiotic adherence and surgical follow through. These issues are magnified for patients with LSIE because they often require longer hospital stays, more intensive imaging, and complex surgeries. From a diagnostic standpoint, engaging patients in care is also crucial: e.g., arranging outpatient follow up echocardiograms can ensure that any delayed complications of LSIE are caught early. Without addressing these broader issues, even perfect diagnostic practice may fail to translate into better outcomes for many IVDUs [39].

## Conclusions

In summary, our analysis indicates that LSIE in IVDUs is far more common than traditionally appreciated and is associated with severe complications. Older studies typically reported LSIE as a rare event [30], but contemporary evidence shows it now accounts for roughly one-third of cases. LSIE tends to occur in older IVDUs with more comorbidities and carries higher rates of embolic events, heart failure, and peri-annular complications. Clinicians must therefore adopt aggressive diagnostic strategies, including early TEE for all suspected IE in IVDUs, to ensure that left-sided involvement is not overlooked. While RSIE (predominantly tricuspid) remains the most common presentation with relatively better outcomes, the rising incidence of left-sided disease demands vigilant echocardiographic evaluation and coordinated care to improve patient outcomes [30, 35].

### Clinical implications

The evolving epidemiology of IVDU-IE implies that current screening and management protocols may need updating. Given the high prevalence of LSIE we observed, the practice of relying solely on TTE (especially in resource-constrained settings) may miss a substantial fraction of cases. According to expert recommendations, TEE should be strongly considered early in IVDU-IE, particularly if TTE is nondiagnostic or if the clinical picture suggests systemic involvement [35]. Furthermore, standardized care pathways – combining addiction treatment with prompt diagnostics and follow up represent best practice but are not yet universally implemented. Our findings suggest that harmonizing study and clinical protocols (e.g. echo timing, surgical criteria, follow-up schedules) will be important for future research and for improving care of intravenous drug use with IE.

## Supplementary Information

Below is the link to the electronic supplementary material.

Supplementary Material 1

Supplementary Material 2

Supplementary Material 3

Supplementary Material 4

## Data Availability

The data supporting the findings of this study are available from the corresponding author upon reasonable request. All extracted datasets, analysis scripts, and supplementary materials used for meta-analyses are stored in a secured institutional repository and can be shared with qualified researchers for academic and non-commercial purposes following approval of a data use agreement.

## Abbreviations

IE: Infective Endocarditis
IVDU: Intravenous Drug Use (or Intravenous Drug User)
RSIE: Right-Sided Infective Endocarditis
LSIE: Left-Sided Infective Endocarditis
TEE: Transesophageal Echocardiography
TTE: Transthoracic Echocardiography
MRSA: Methicillin-Resistant Staphylococcus aureus
MSSA: Methicillin-Sensitive Staphylococcus aureus
HIV: Human Immunodeficiency Virus
CI: Confidence Interval
