# Supplementary material for: Valve involvement in infective endocarditis among intravenous drug users: a systematic review and meta-analysis
Source: BMC Infect Dis. 2026 May 12;26:1262. doi: 10.1186/s12879-026-13284-9 (PMC13343716; doi:10.1186/s12879-026-13284-9)
Supplement: Supplementary file 1 — Supplementary Material 1 [file 12879_2026_13284_MOESM1_ESM.docx]

# **Appendix table 1. Incuded studies details**

| **No.** | **Study** | **Title** | **Author** |
| --- | --- | --- | --- |
| **1** | Asgeirsson 2016 | Low mortality but increasing incidence of Staphylococcus aureus endocarditis in people who inject drugs: Experience from a Swedish referral hospital | H. Asgeirsson, A. Thalme and O. Weiland |
| **2** | Low 2020 | Burden of infective endocarditis in an Australian cohort of people who inject drugs | Z. M. Low, S. Krishnaswamy, I. J. Woolley, R. L. Stuart, A. Boers, T. L. Barton and T. M. Korman |
| **3** | Arora 2021 | Changing spectrum of infective endocarditis in India: An 11-year experience from an academic hospital in North India | N. Arora, P. K. Panda, P. Cr, L. Uppal, A. Saroch, A. Angrup, N. Sharma, Y. P. Sharma, R. Vijayvergiya, M. K. Rohit, A. Gupta, B. K. Sihag, H. Gupta, N. Dahiya, A. Bahl, P. Singh, S. Mehrotra, P. Barwad and A. K. Pannu |
| **4** | Hilbig 2020 | Infective Endocarditis in the Intravenous Drug Use Population at a Tertiary Hospital in Melbourne, Australia | A. Hilbig and A. Cheng |
| **5** | Damlin 2021 | Patients with infective endocarditis and history of injection drug use in a Swedish referral hospital during 10 years | A. Damlin and K. Westling |
| **6** | Adams 2022 | Peripherally Inserted Central Catheter Line Misuse Among People Who Inject Drugs While on Therapy for Infective Endocarditis | J. Adams, T. Elton-Marshall, E. Shojaei and M. Silverman |
| **7** | Balda 2021 | Recent Trends in Infective Endocarditis Among Patients with and Without Injection Drug Use: An Eight-Year Single Center Study | J. Balda, R. Alpizar-Rivas, S. Elarabi, B. L. Jaber and C. Nader |
| **8** | Meel 2018 | Striking increase in the incidence of infective endocarditis associated with recreational drug abuse in urban South Africa | R. Meel and M. R. Essop |
| **9** | De Rosa 2007 | Infective endocarditis in intravenous drug users from Italy: The increasing importance in HIV-infected patients | F. G. De Rosa, S. Cicalini, F. Canta, S. Audagnotto, E. Cecchi and G. Di Perri |
| **10** | Jain 2008 | Infective endocarditis in an urban medical center: Association of individual drugs with valvular involvement | V. Jain, M. H. Yang, G. Kovacicova-Lezcano, L. S. Juhle, A. F. Bolger and L. G. Winston |
| **11** | Huang 2020 | Left-sided infective endocarditis in persons who inject drugs | G. Huang, K. A. Davis, S. A. Petty, W. A. Tan, E. W. Barnes and J. E. Peacock |
| **12** | Syed 2021a | Injection Drug Use Endocarditis: An Inner-City Hospital Experience | I. M. Syed, B. Yanagawa, S. Jeyaganth, S. Verma and A. N. Cheema |
| **13** | Goyal 2020 | Clinical characteristics and outcome of infective endocarditis among intravenous drug abusers in India | A. Goyal, B. Mohan, P. Kumar, D. Gupta, R. Tandon, S. Singla, G. Singh, B. Singh, S. T. Chhabra, N. Aslam and G. S. Wander |
| **14** | Rodger 2018 | Clinical Characteristics and Factors Associated With Mortality in First-Episode Infective Endocarditis Among Persons Who Inject Drugs | Rodger L, Glockler-Lauf SD, Shojaei E, et al. |

**Appendix table 2. Diagnostic Criteria Used to Define Infective Endocarditis**

| Study | Country | Diagnostic Criteria Used | Included “Definite” Only | Included “Possible” IE | Note |
| --- | --- | --- | --- | --- | --- |
| Asgeirsson 2016 | Sweden | Modified Duke | Yes | Yes | Cohort included all verified SAE episodes (35% were left-sided/definite). |
| Low 2020 | Australia | Modified Duke | Yes | Yes | Table 1 in study shows "Possible" cases were included. |
| Arora 2021 | India | Modified Duke | Yes | No | "Only cases of definite IE... were included." |
| Hilbig 2020 | Australia | Duke | Yes | Yes | Abstract states: "Included cases met Duke Criteria for 'definite' or 'possible' IE." |
| Damlin 2021 | Sweden | Modified Duke | Yes | Yes | Data obtained from the Swedish National Registry which includes both categories. |
| Adams 2022 | Canada | Modified Duke | Yes | No | Study states patients identified "with Modified Duke Criteria for definite IE." |
| Balda 2021 | USA | Modified Duke | Yes | No | Authors performed manual chart review to confirm cases as "definite IE." |
| Meel 2018 | South Africa | Modified Duke | Yes | No | All 68 patients in this cohort were "definite" IE cases. |
| De Rosa 2007 | Italy | Duke | Yes | No | "Only definite diagnosis according to the Duke criteria were analyzed." |
| Jain 2008 | USA | Duke | Yes | Yes | Methods specify a mix (76% definite, 24% possible). |
| Huang 2020 | USA | Modified Duke | Yes | No | Verified by ICD codes and manual confirmation of "definite IE." |
| Syed 2021a | Canada | Duke | Yes | No | States inclusion of patients with "definite infective endocarditis." |
| Goyal 2020 | India | Modified Duke | Yes | No | Explicitly states only "definite" IE cases were included. |
| Rodger 2018 | Canada | Modified Duke | Yes | No | Included only patients meeting criteria for "definite" IE. |

# **Appendix table 3. Mortality & Follow-up Table**

| **Study** | **Country** | **Follow-up Duration** | **Type of Mortality Reported** |
| --- | --- | --- | --- |
| Asgeirsson 2016 | Sweden | In-hospital & 30-day | All-cause |
| Low 2020 | Australia | In-hospital & 1-year | All-cause |
| Arora 2021 | India | N/A | All-cause |
| Hilbig 2020 | Australia | N/A | In-hospital mortality |
| Damlin 2021 | Sweden | N/A | In-hospital mortality |
| Adams 2022 | Canada | Mean 1.3 years | All-cause |
| Balda 2021 | USA | N/A | In-hospital mortality |
| Meel 2018 | South Africa | N/A | In-hospital mortality |
| De Rosa 2007 | Italy | N/A | In-hospital mortality |
| Jain 2008 | USA | N/A | In-hospital mortality |
| Huang 2020 | USA | 10 weeks | All-cause |
| Syed 2021a | Canada | Mean 14 months | All-cause |
| Goyal 2020 | India | N/A | In-hospital mortality |
| Rodger 2018 | Canada | 1 year | All-cause |

# **Appendix table 4. Preliminary meta-analysis**

| **Variable (MA)** | **Effect size** | **Heterogeneity** | | | **Funnel**  **deviation** | **Egger’s**  **p-val** | **F-test**  **p-val** |
| --- | --- | --- | --- | --- | --- | --- | --- |
|  |  | **I²** | **tau²** | **H²** |  |  |  |
| Tricuspid valve (%) | 0.59 [0.53 – 0.64] | 74% | 0.099 | 3.72 | Slight | 0.120 | 0.503 |
| Pulmonic valve (%) | 0.01 [0.00 – 0.03] | 7% | 0.762 | 1.07 | Slight | 0.007 | 0.006 |
| Mitral valve (%) | 0.21 [0.18 – 0.26] | 65% | 0.097 | 2.62 | None | 0.020 | 0.202 |
| Aortic valve (%) | 0.16 [0.12 – 0.20] | 71% | 0.197 | 1.44 | Skewed | 0.003 | 0.001 |
| Left heart (%) | 0.39 [0.33 – 0.46] | 73% | 0.084 | 3.17 | None | 0.289 | 0.907 |
| Right heart (%) | 0.59 [0.54 – 0.65] | 56% | 0.035 | 1.20 | Slight | 0.010 | 0.075 |
| Both sides (%) | 0.08 [0.06 – 0.12] | 56% | 0.134 | 1.51 | None | 0.006 | 0.078 |
| Right heart risk (RR) | 1.49 [1.15 – 1.95] | 78% | 0.087 | 5.71 | None | 0.132 | 0.524 |
| S. aureus (%) | 0.73 [0.58 – 0.85] | 93% | 1.102 | 14.06 | Slight | 0.329 | 0.009 |
| MRSA (%) | 0.17 [0.06 – 0.38] | 95% | 1.943 | 12.99 | Slight | 0.500 | 0.009 |
| MSSA (%) | 0.63 [0.50 – 0.74] | 86% | 0.592 | 10.21 | Slight | 0.171 | 0.020 |
| Non-viridans S. (%) | 0.10 [0.05 – 0.19] | 90% | 0.755 | 6.24 | None | 0.630 | 0.090 |
| S. viridans (%) | 0.07 [0.02 – 0.28] | 97% | 1.568 | 8.32 | Slight | 0.656 | 0.070 |
| Valve surgeries (%) | 0.21 [0.15 – 0.28] | 79% | 0.267 | 5.37 | None | 0.095 | 0.928 |
| Mortality (all-cause) | 0.17 [0.12 – 0.25] | 89% | 0.491 | 5.61 | Slight | 0.363 | 0.010 |

**Appendix table 5. Risk of bias**

| **Study** | **D1** | **D2** | **D3** | **D4** | **D5** | **D6** | **D7** | **Overall** |
| --- | --- | --- | --- | --- | --- | --- | --- | --- |
| Adams 2022 | Some concerns | Some concerns | Low | Low | Some concerns | Some concerns | Low | Some concerns |
| Arora 2021 | Some concerns | Some concerns | Low | Low | Some concerns | Some concerns | Low | Some concerns |
| Asgeirsson 2016 | Low | Low | Low | Low | Some concerns | Low | Low | Low |
| Balda 2021 | Some concerns | Some concerns | Low | Low | Some concerns | Some concerns | Some concerns | Some concerns |
| Damlin 2021 | Low | Some concerns | Low | Low | Some concerns | Low | Low | Low |
| De Rosa 2007 | Low | Some concerns | Some concerns | Low | Some concerns | Some concerns | Low | Some concerns |
| Goyal 2020 | Some concerns | Some concerns | Low | Some concerns | Some concerns | Some concerns | Low | Some concerns |
| Hilbig 2020 | Some concerns | Low | Low | Some concerns | Low | Low | Low | Low |
| Huang 2020 | Some concerns | Some concerns | Low | Low | Some concerns | Low | Low | Some concerns |
| Jain 2008 | Some concerns | Some concerns | High | Low | Some concerns | Low | Some concerns | High |
| Low 2020 | High | Low | Some concerns | High | Some concerns | Low | Some concerns | High |
| Meel 2018 | Some concerns | Some concerns | Low | Low | Some concerns | Some concerns | Low | Some concerns |
| Syed 2021 | High | Low | Some concerns | High | Some concerns | Low | Some concerns | High |
| Rodger 2018 | Low | Low | Low | High | Some concerns | Low | Some concerns | Low |
| ** Footnotes:*  Domain 1: Risk of bias due to confounding  Domain 2: Risk of bias arising from measurement of the exposure  Domain 3: Risk of bias in selection of participants into the study (or into the analysis)  Domain 4: Risk of bias due to post-exposure interventions  Domain 5: Risk of bias due to missing data  Domain 6: Risk of bias arising from measurement of the outcome  Domain 7: Risk of bias in selection of the reported result | | | | | | | | |


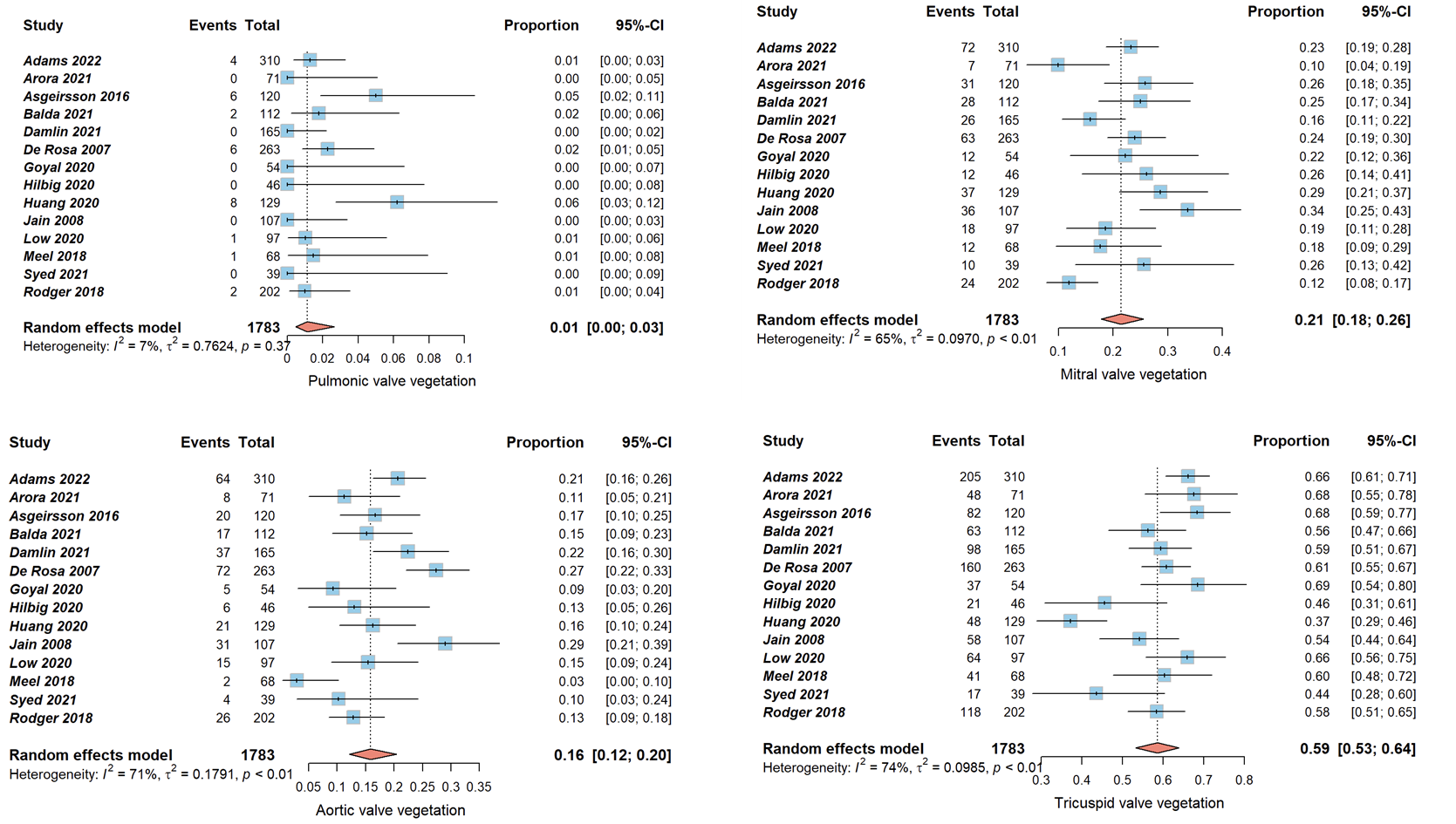


**Figure 1.** Forest plot: Pooled prevalence of heart valves involvement in IVDU-associated IE


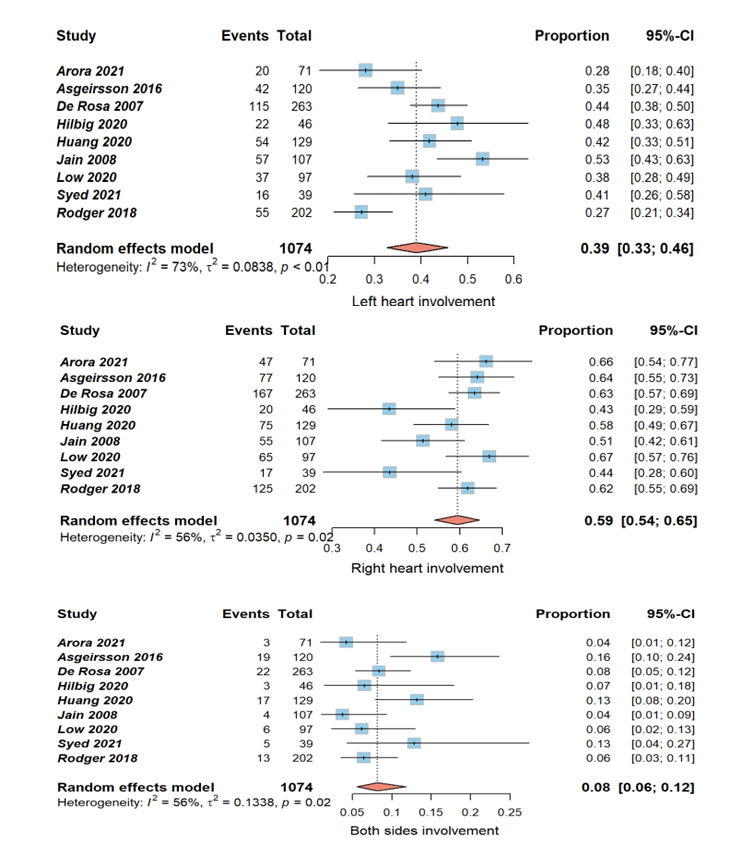


**Figure 2.** Forest plot: Pooled prevalence of heart side involvement in IVDU-associated IE


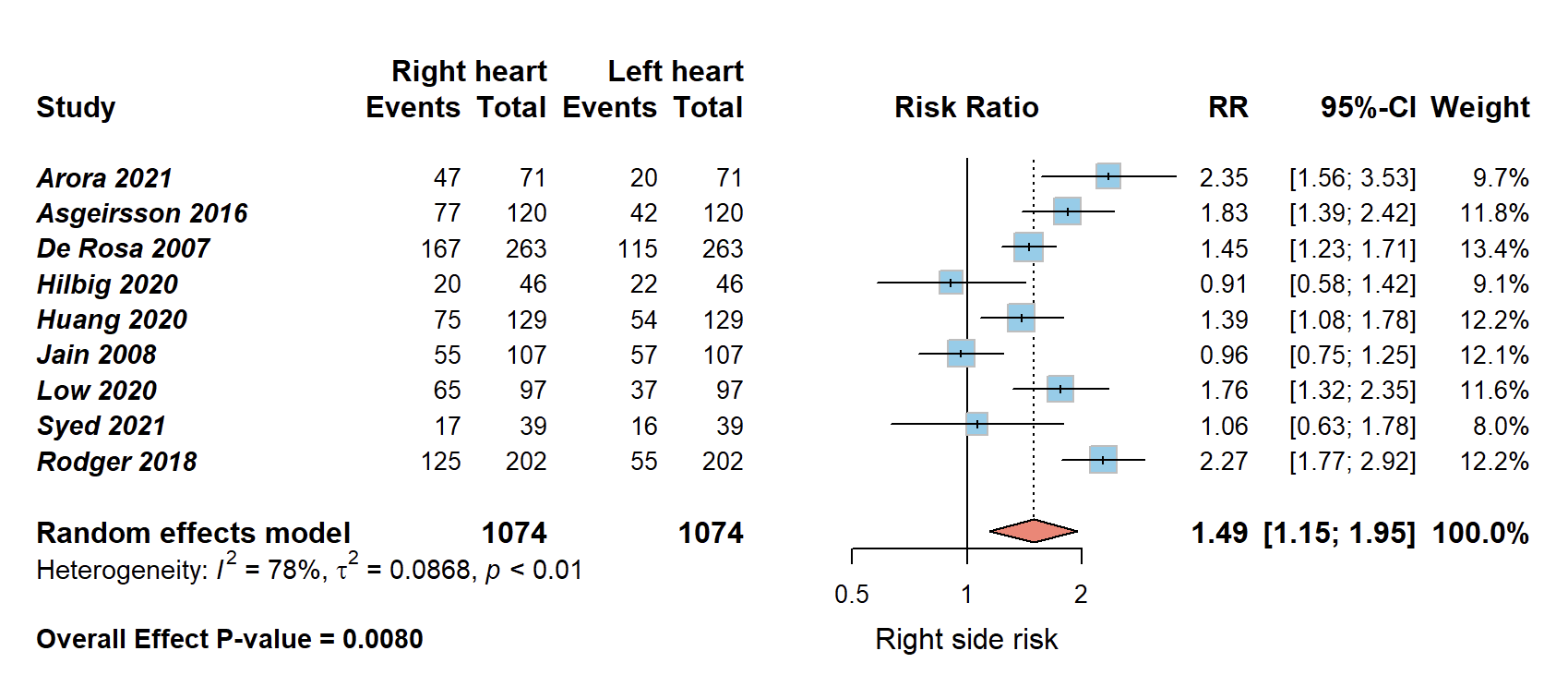


**Figure 3.** Forest plot: Relative risk of right-sided IE in IVDUs vs. left-sided IE

**Tricuspid valve involvement**


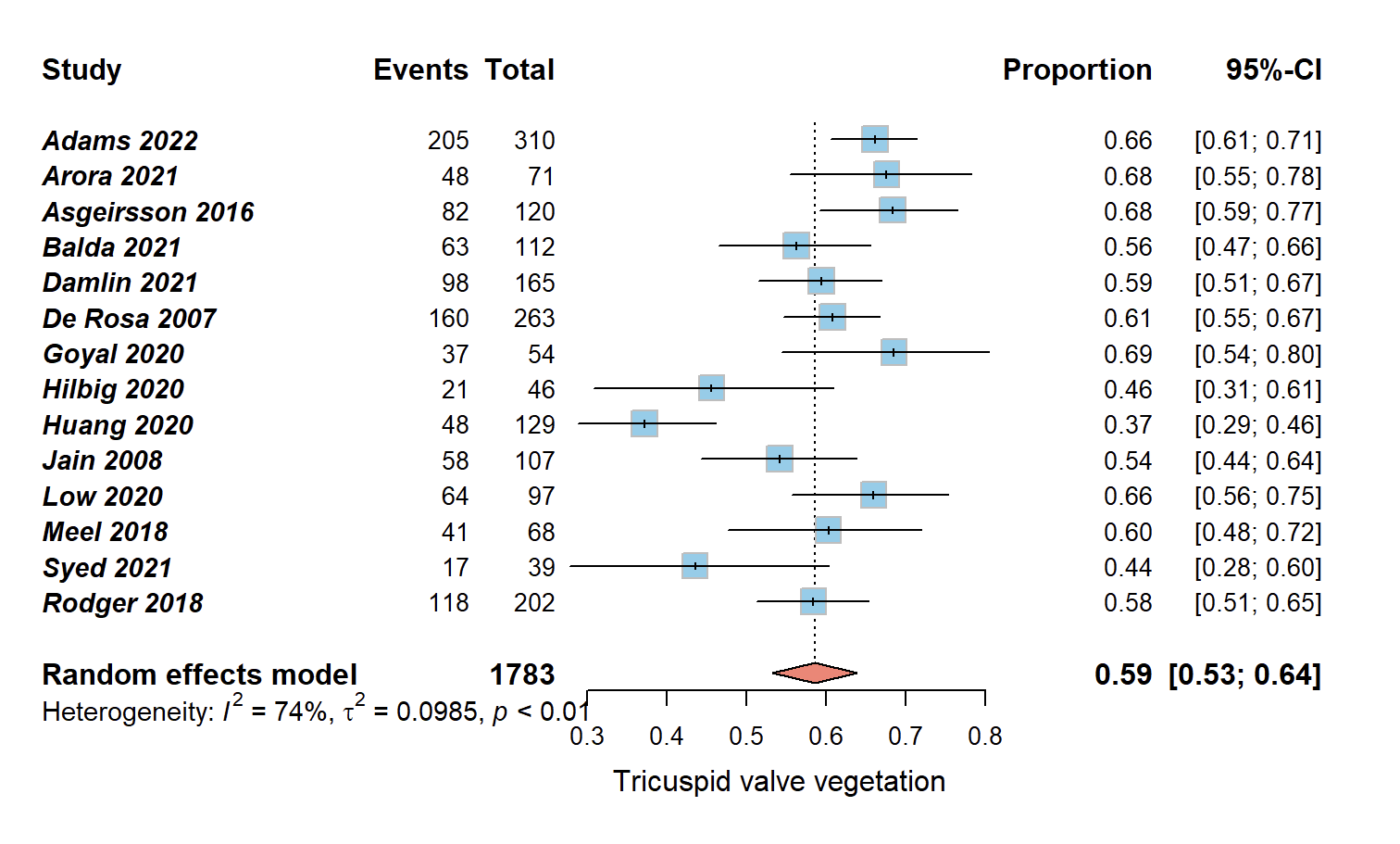


**Figure 4.** Forest plot: Pooled prevalence of tricuspid valve involvement in IVDU-associated IE


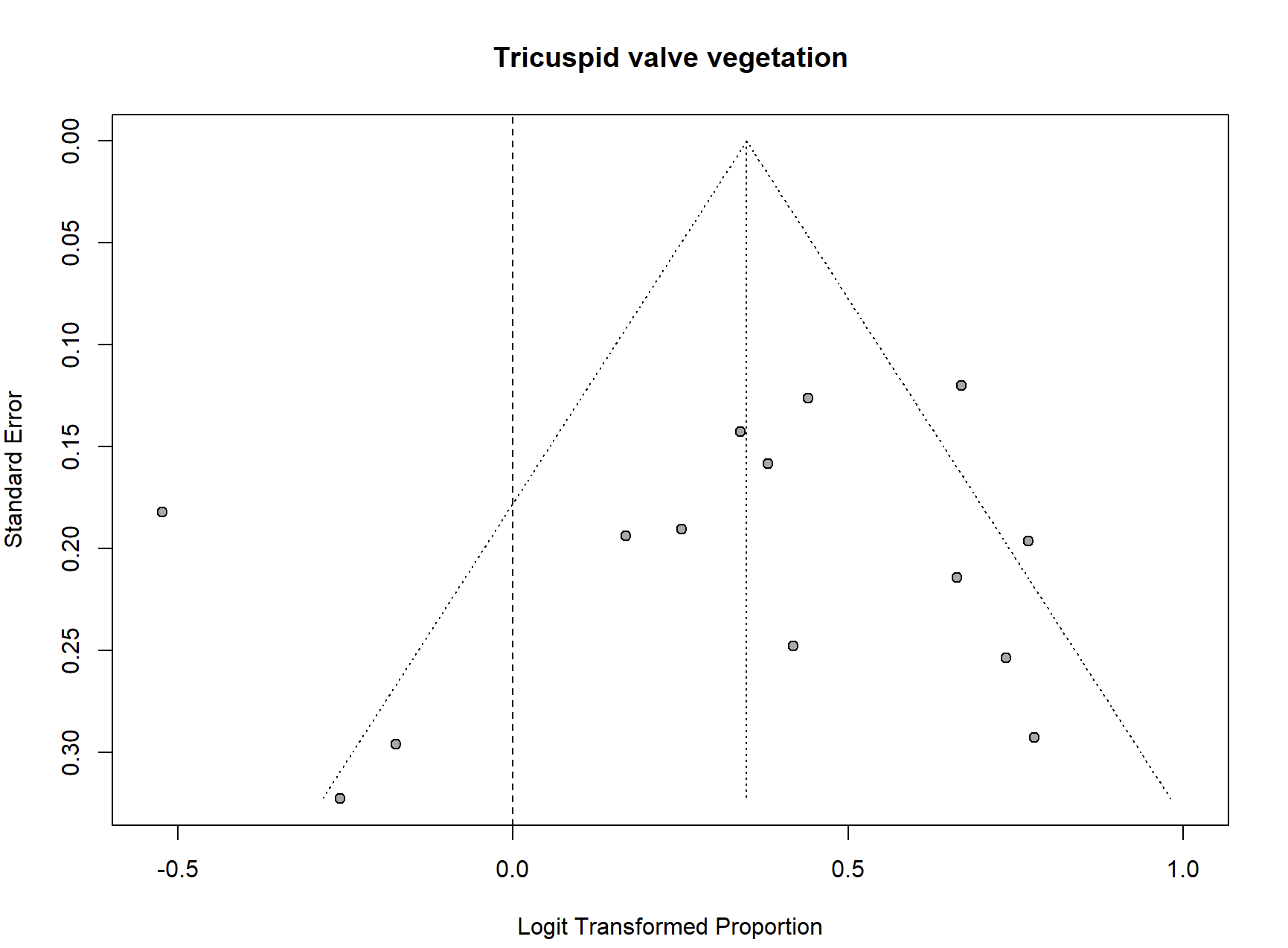


**Figure 5**. Funnel plot: Tricuspid valve involvement

**Pulmonic valve involvement**


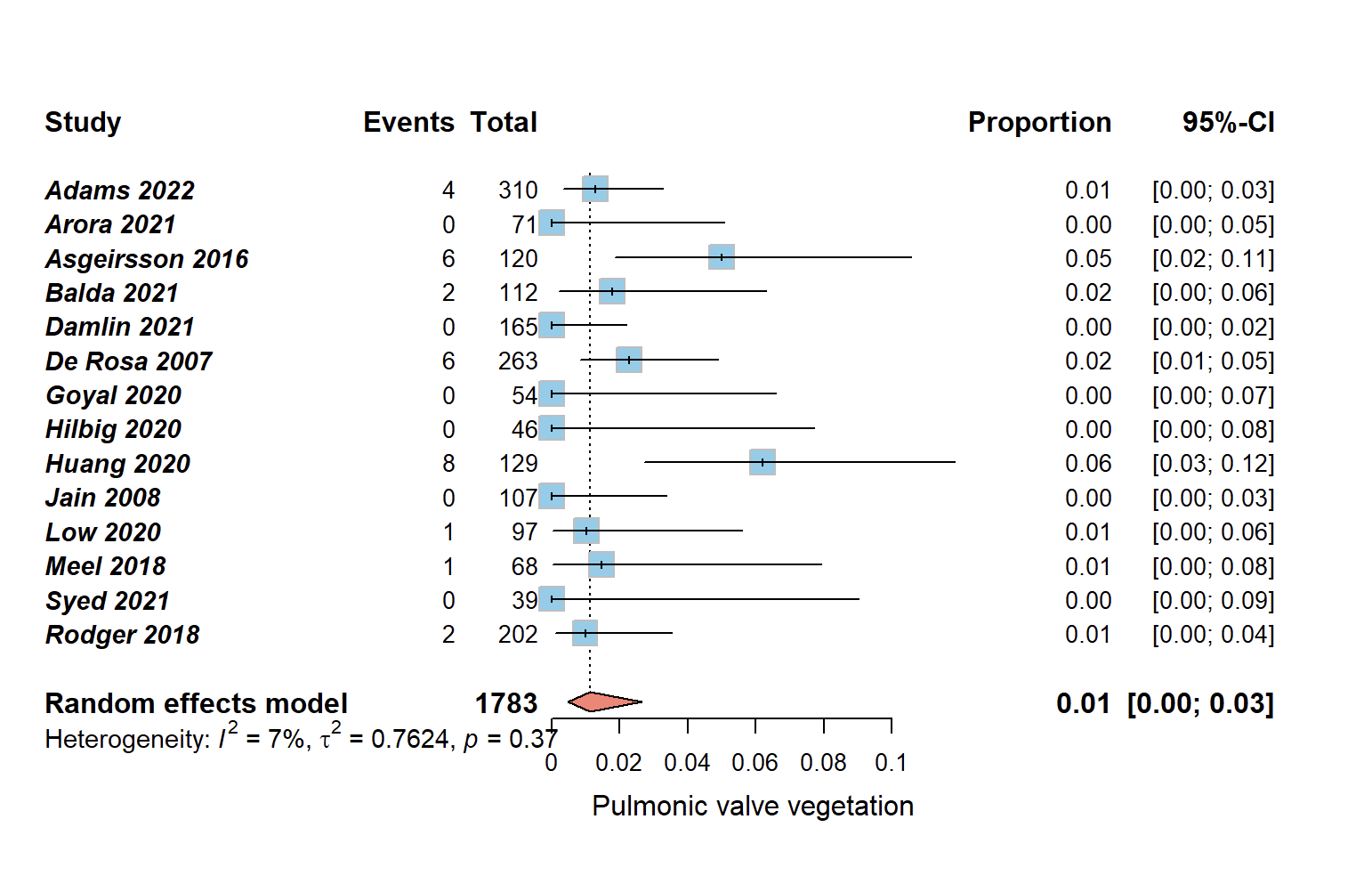


**Figure 6.** Forest plot: Pooled prevalence of pulmonic valve involvement in IVDU-associated IE


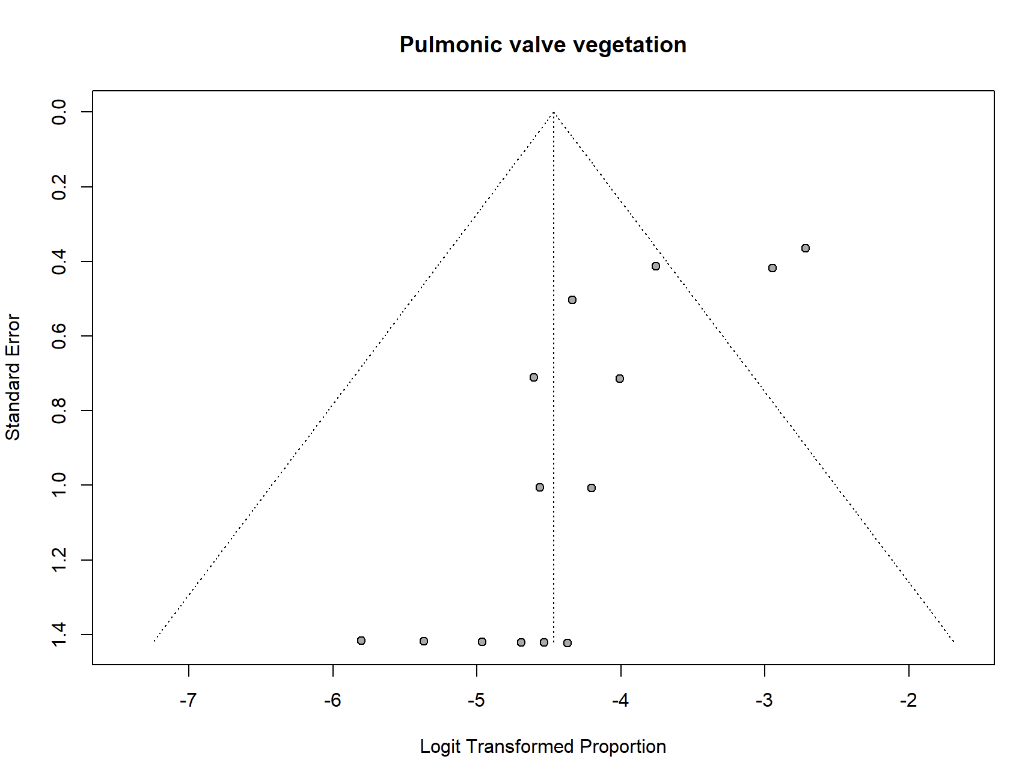


**Figure 7.** Funnel Plot of Logit-Transformed Proportions for pulmonic valve vegetation

**Mitral valve involvement**


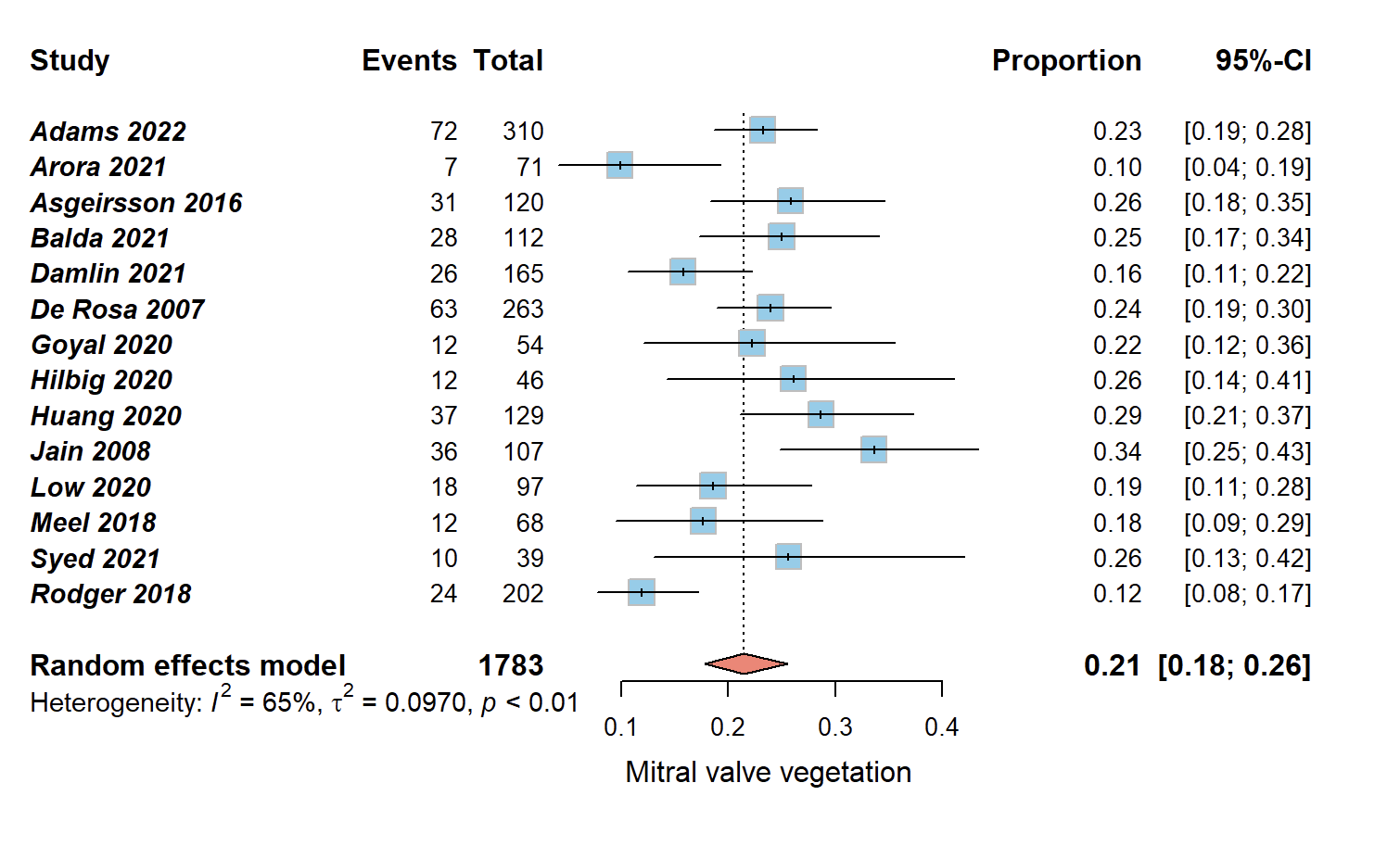


**Figure 8.** Forest plot: Pooled prevalence of mitral valve involvement in IVDU-associated IE
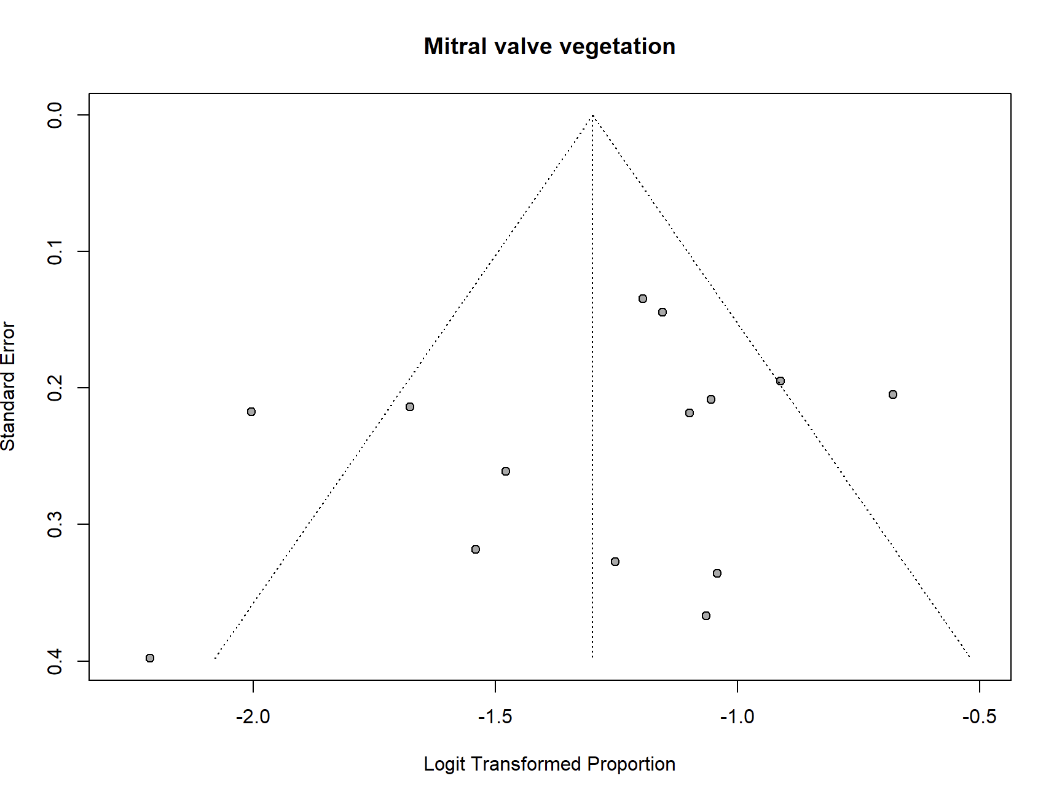


**Figure 9.** Funnel plot: Mitral valve involvement

**Aortic valve involvement**


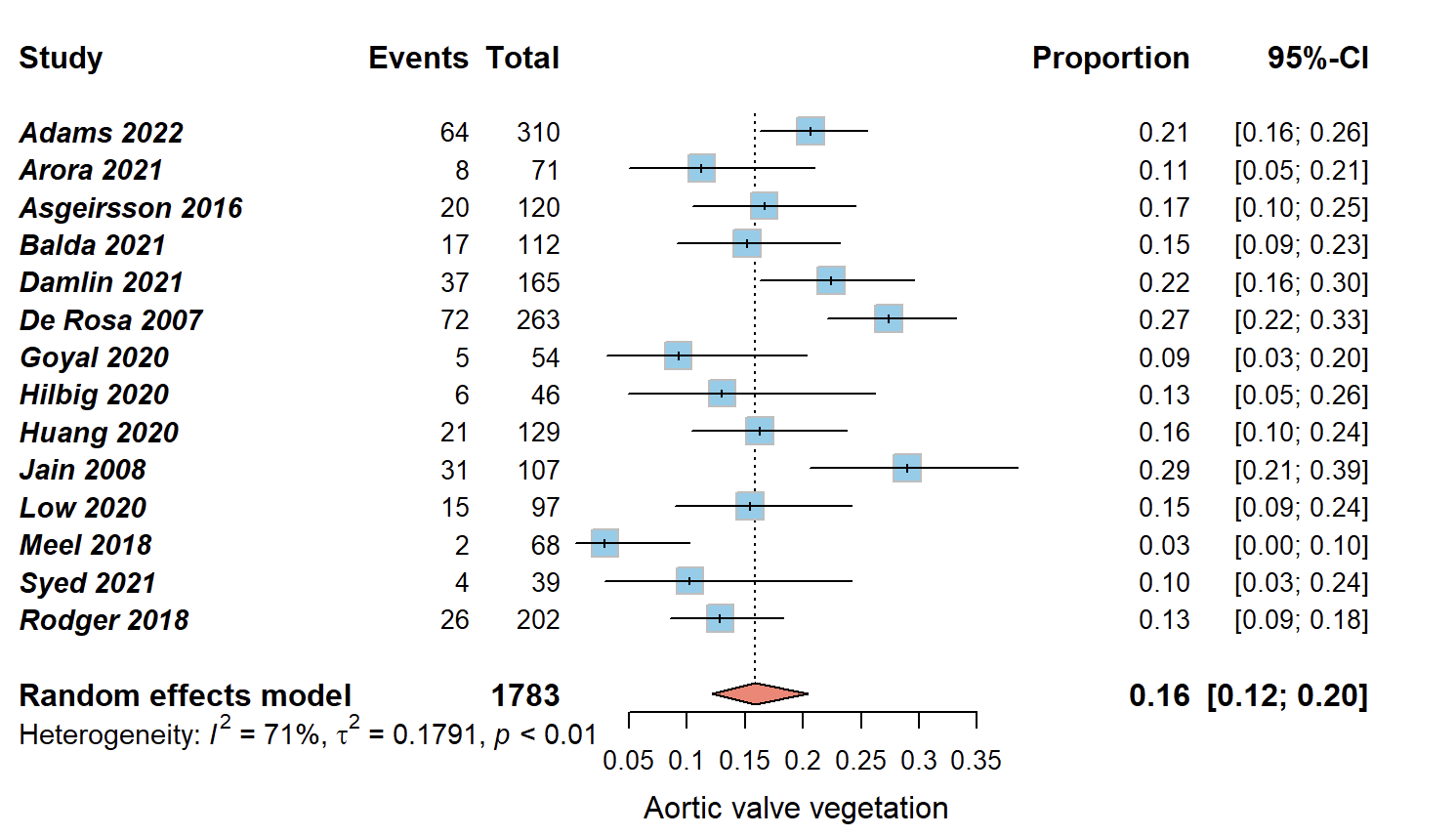


**Figure 10.** Forest plot: Pooled prevalence of aortic valve involvement in IVDU-associated IE
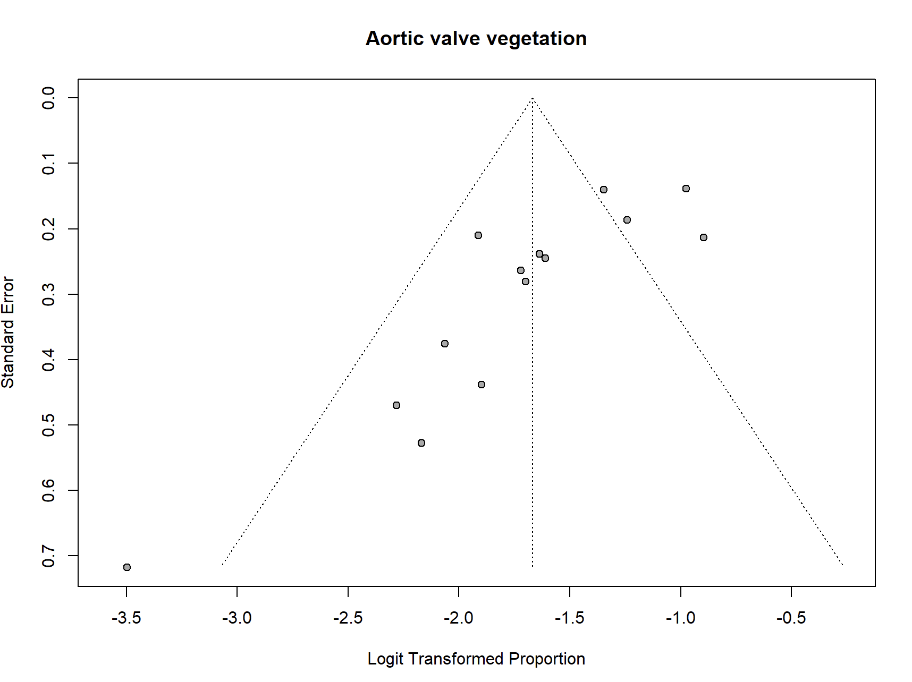


**Figure 11.** Funnel plot: Aortic valve involvement

**Left heart involvement**


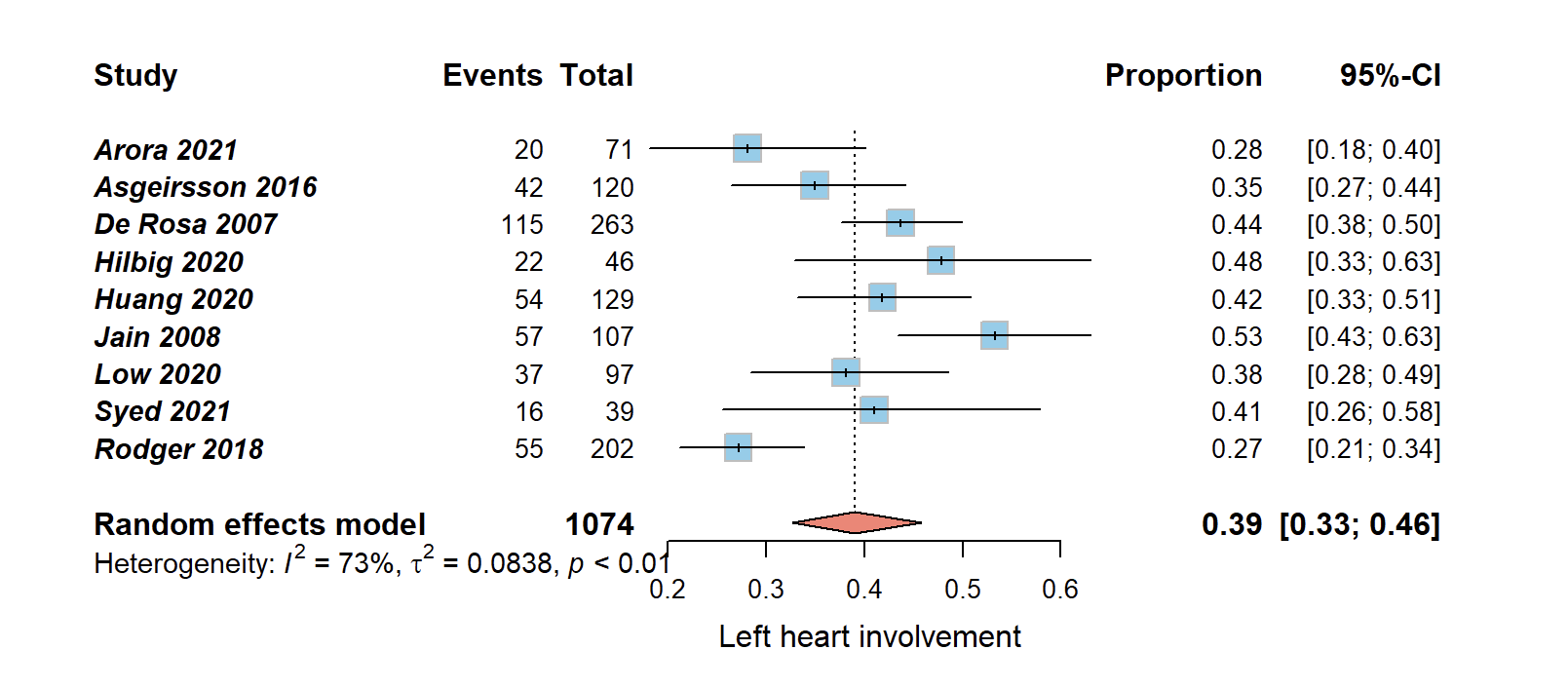


**Figure 12.** Forest plot of pooled prevalence of left-sided infective endocarditis in IVDUs


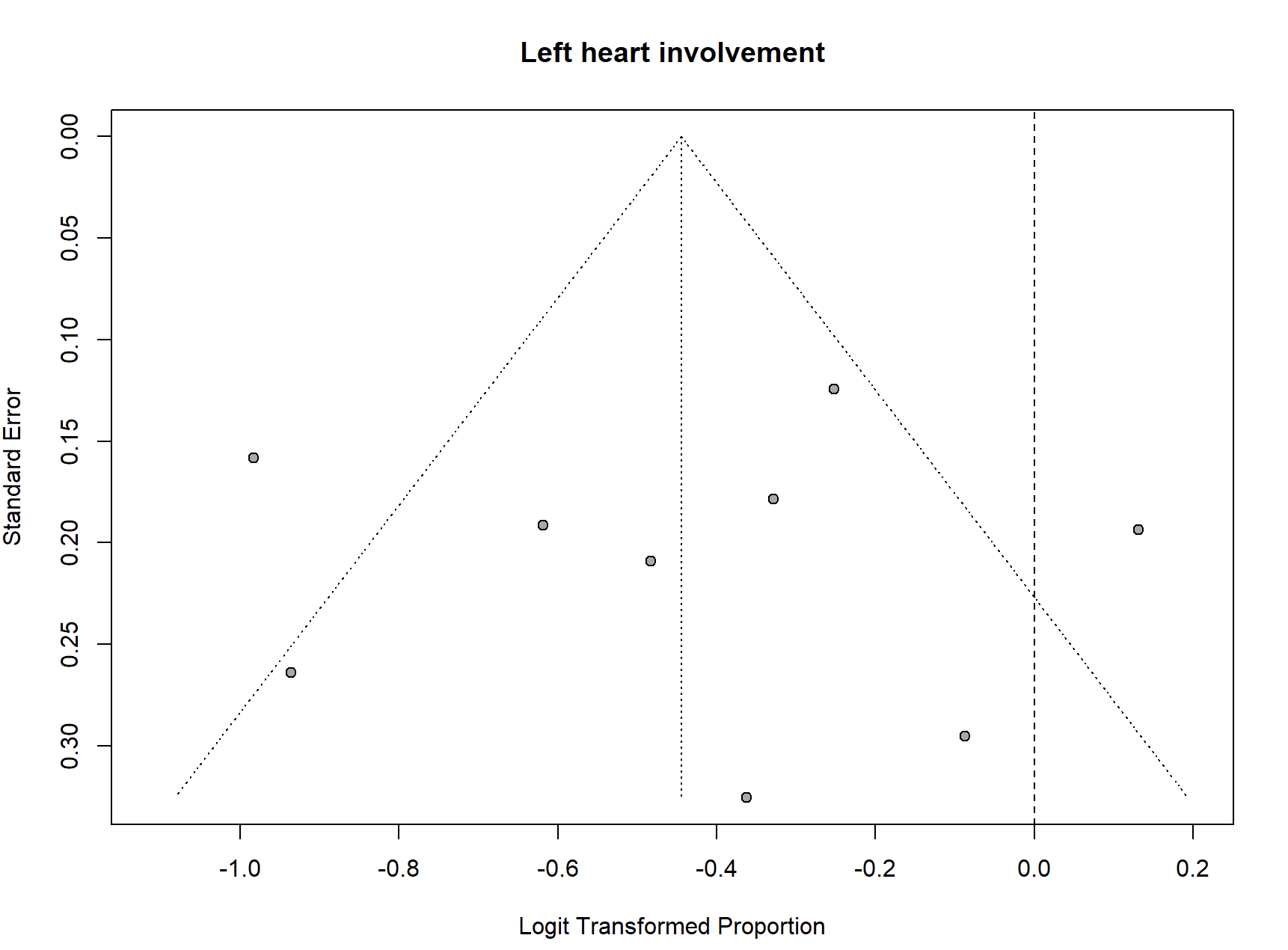


**Figure 13.** Funnel plot of logit-transformed proportions for left heart involvement

**Right heart involvement**


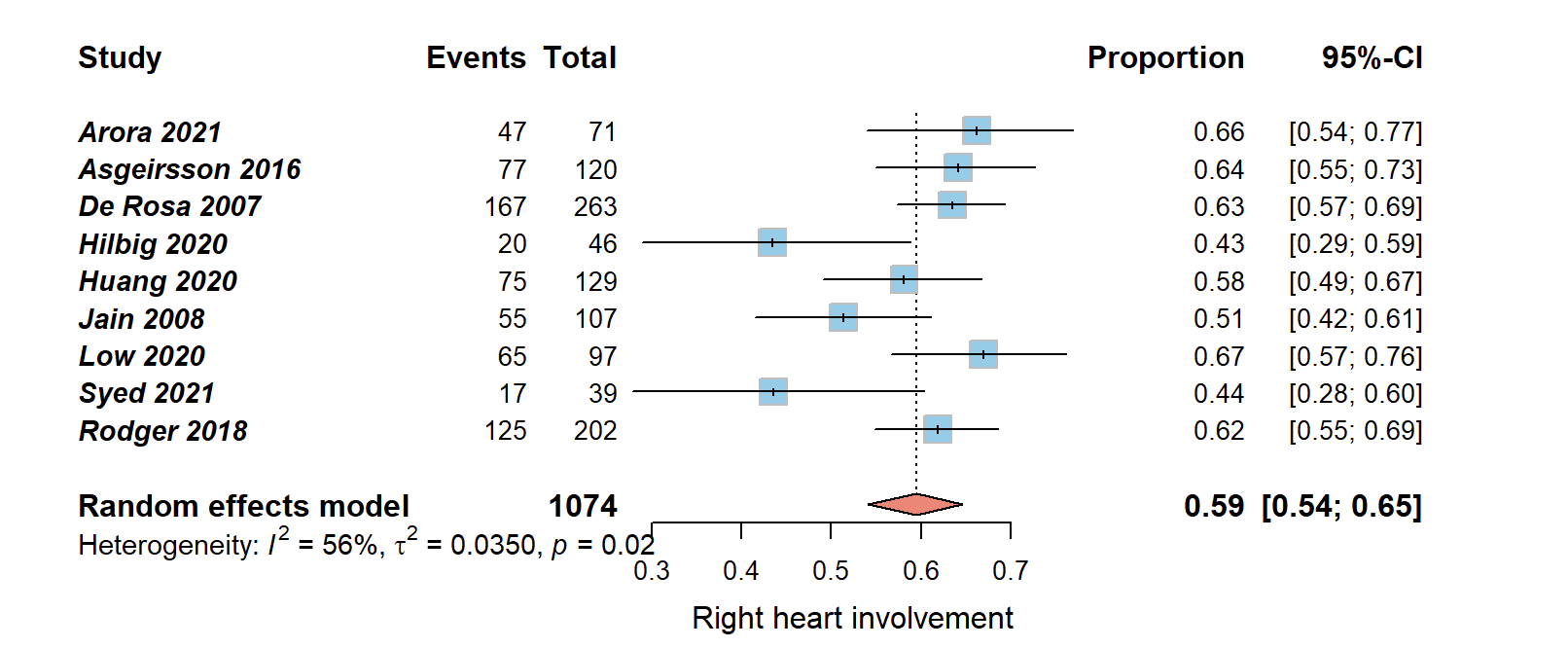


**Figure 14.** Forest plot: Pooled prevalence of right-sided endocarditis in IVDUs
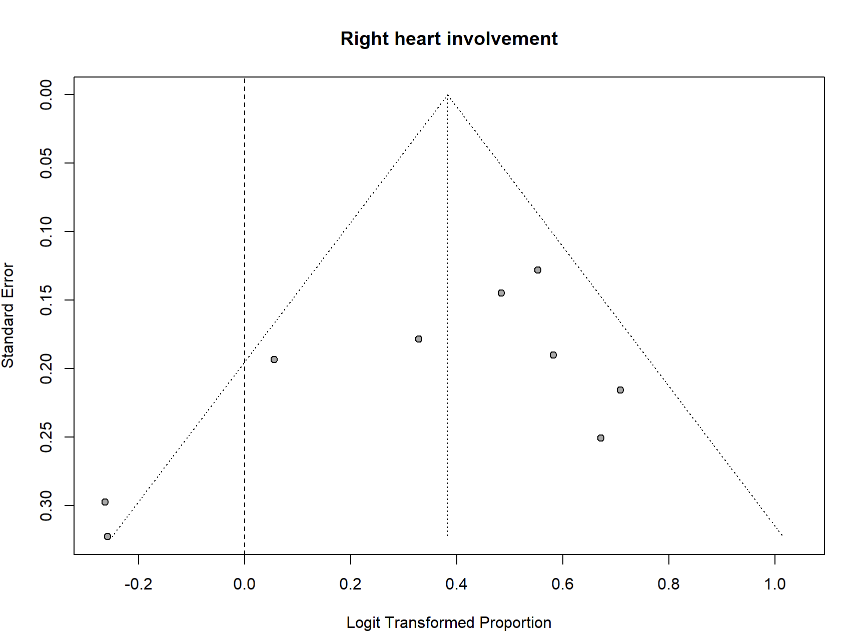


**Figure 15.** Funnel plot of logit-transformed proportions for right heart involvement

**Both sides valve involvement**


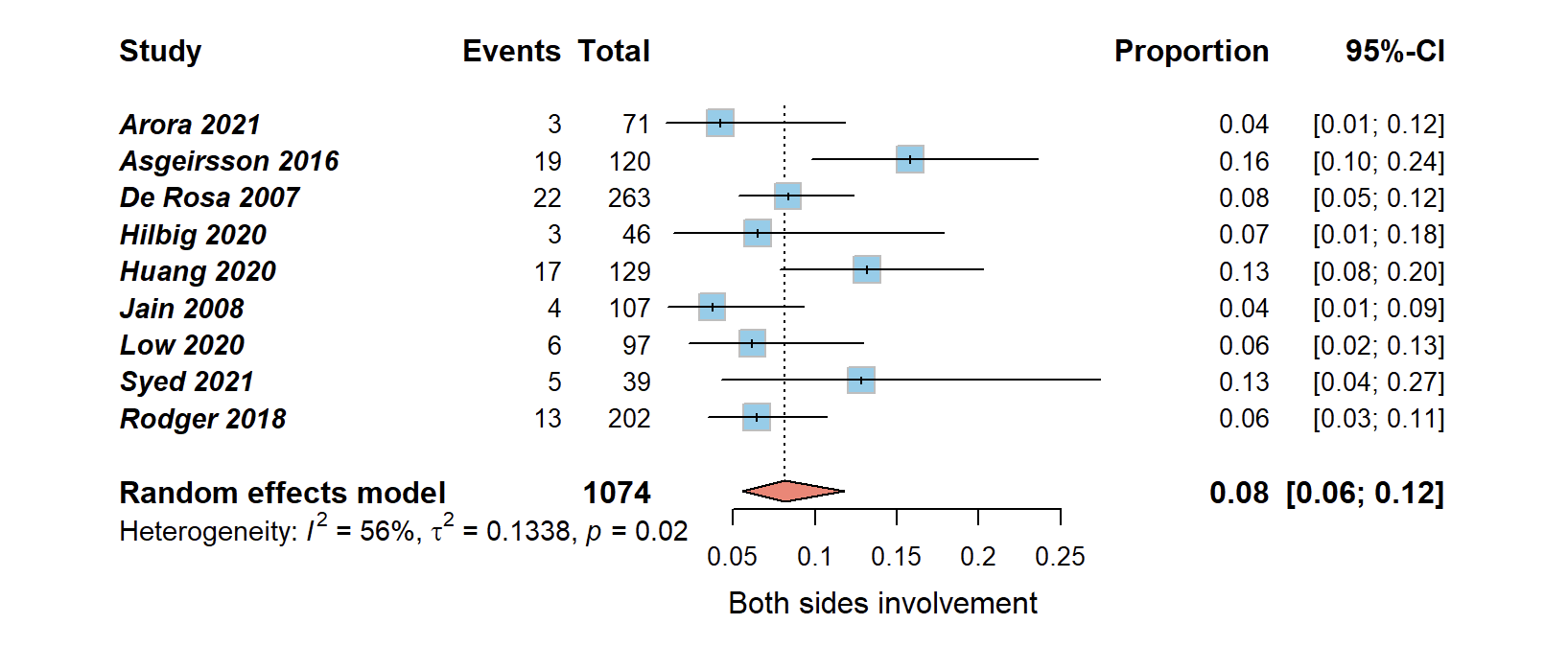


**Figure 16.** Forest plot: Pooled prevalence of both-sided valve involvement in IVDUs*
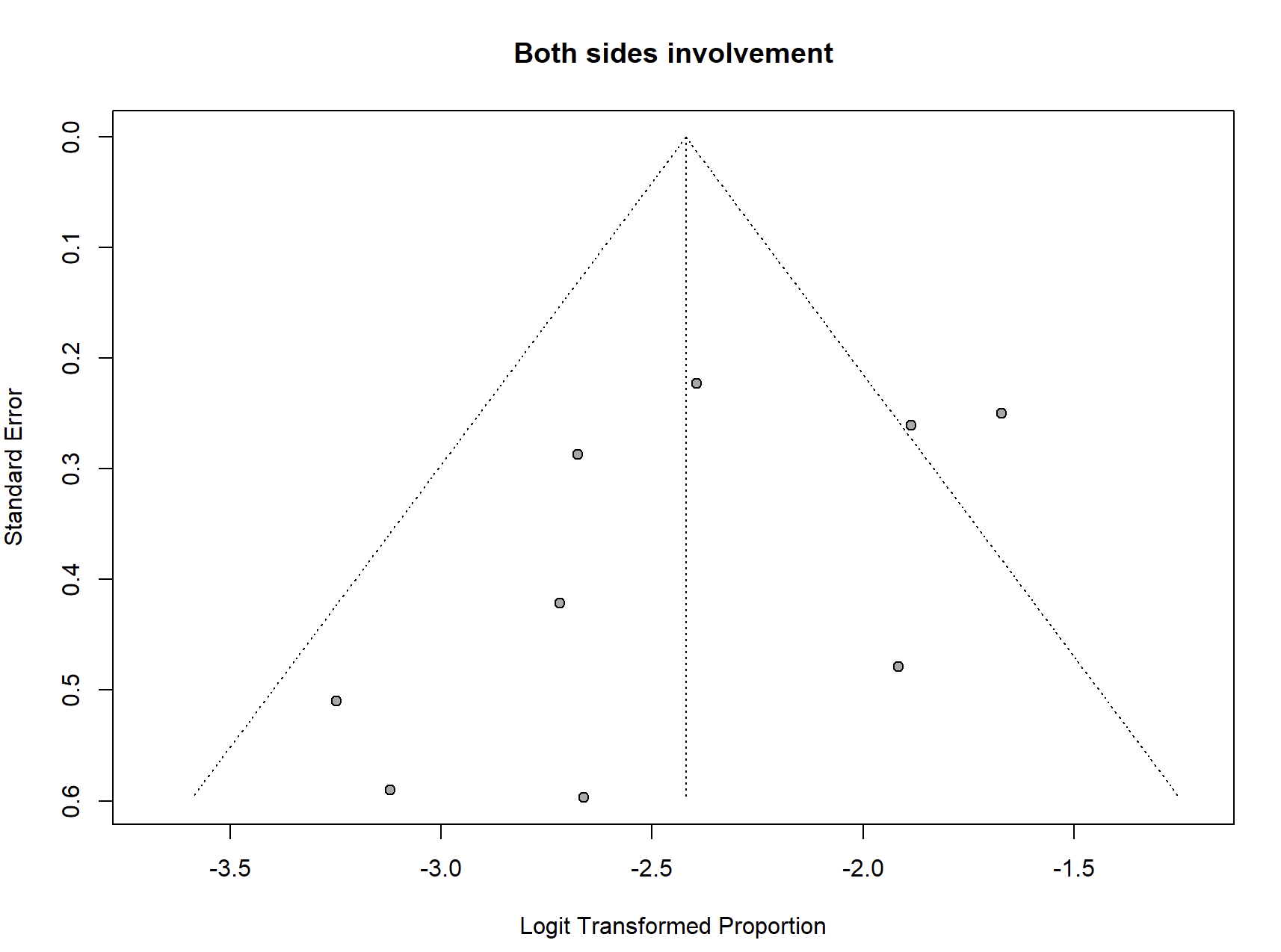
*

**Figure 17.** Funnel plot of logit transformed proportions for both sides involvement

**Right sides risk**


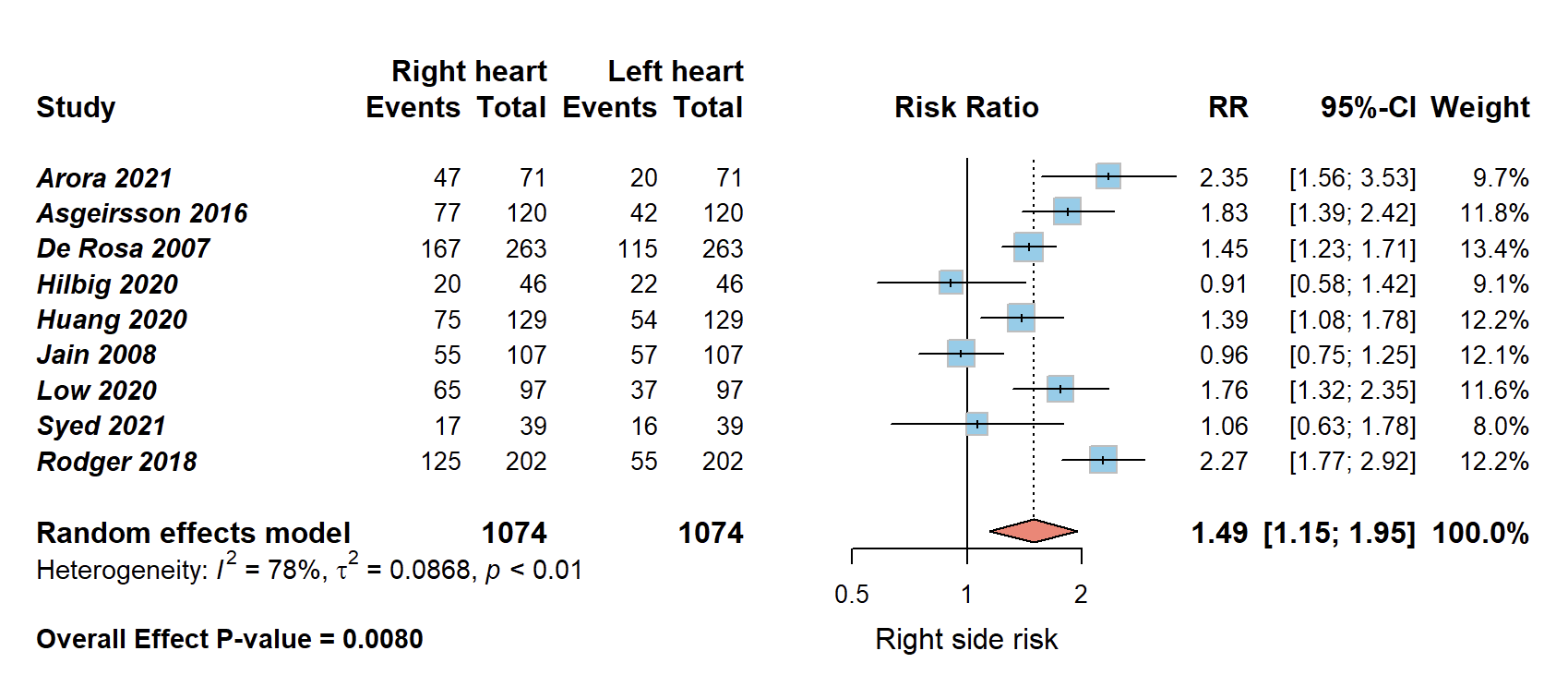


**Figure 18.** Forest plot: Relative risk of right-sided IE in IVDUs vs. left-sided IE


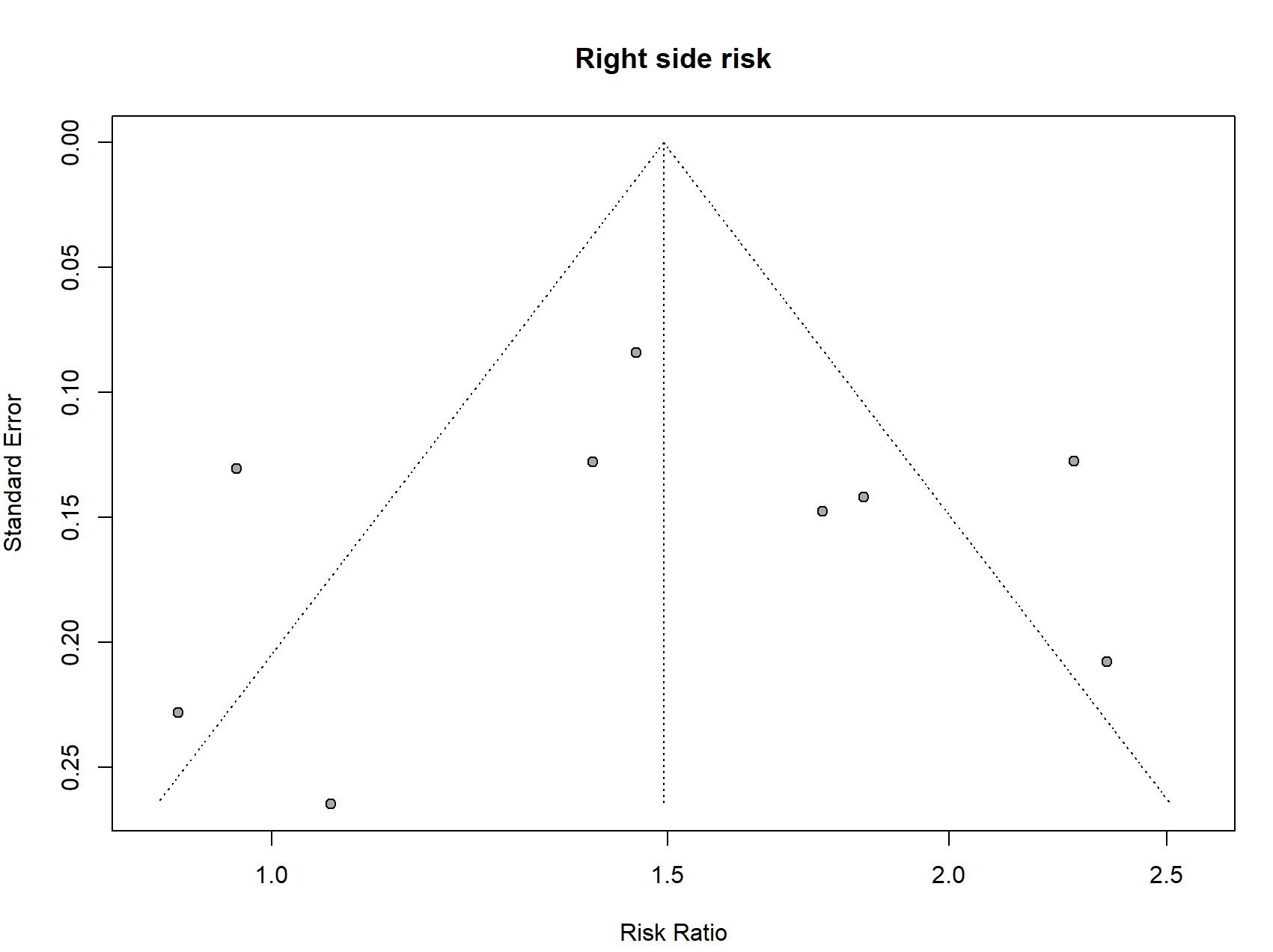


**Figure 19.** Funnel plot of logit-transformed proportions for right side risk

**Staphylococcus aureus positive**


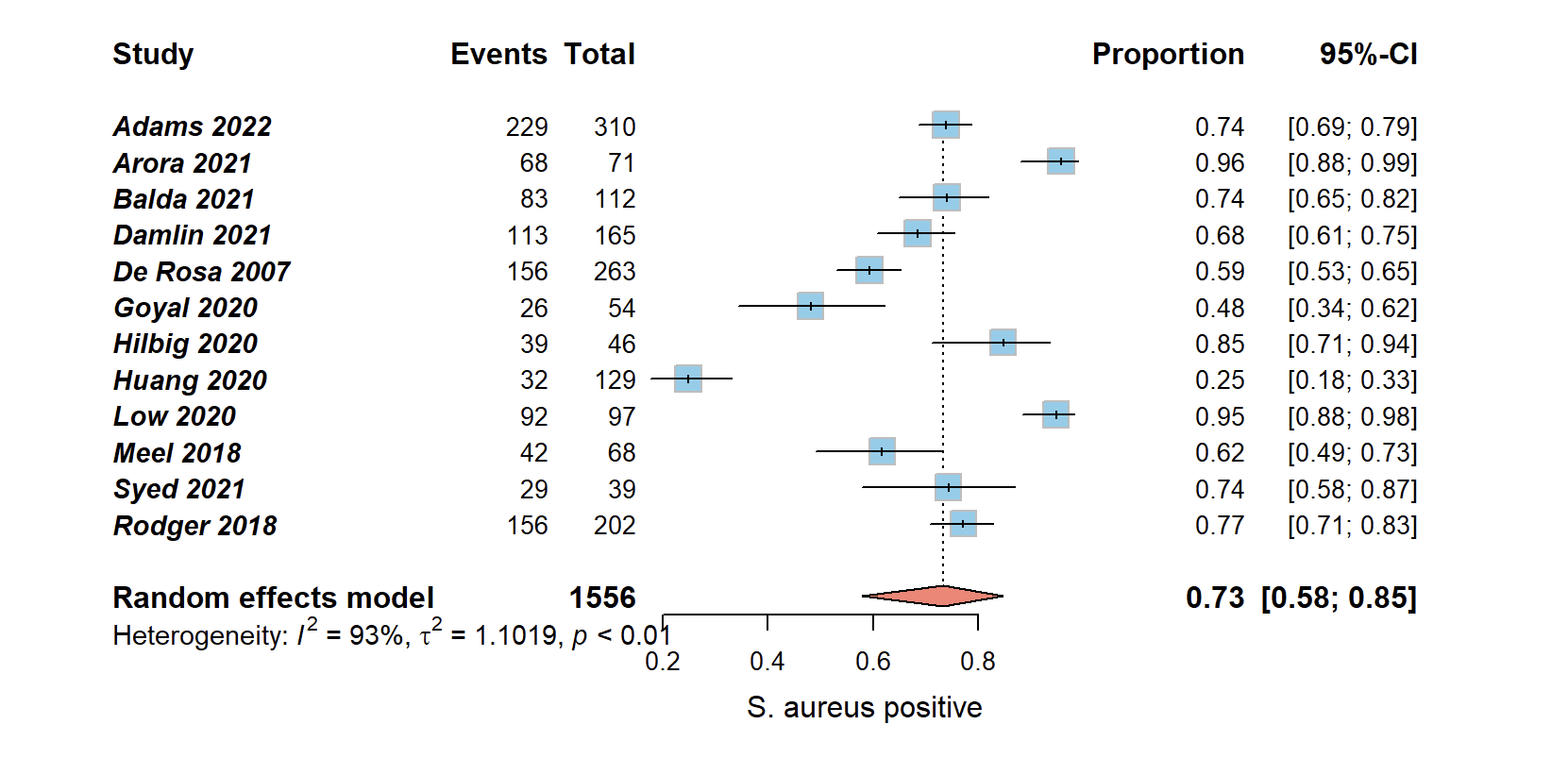


**Figure 20.** Forest plot: Proportion of S. aureus positive IVDU-associated IE cases


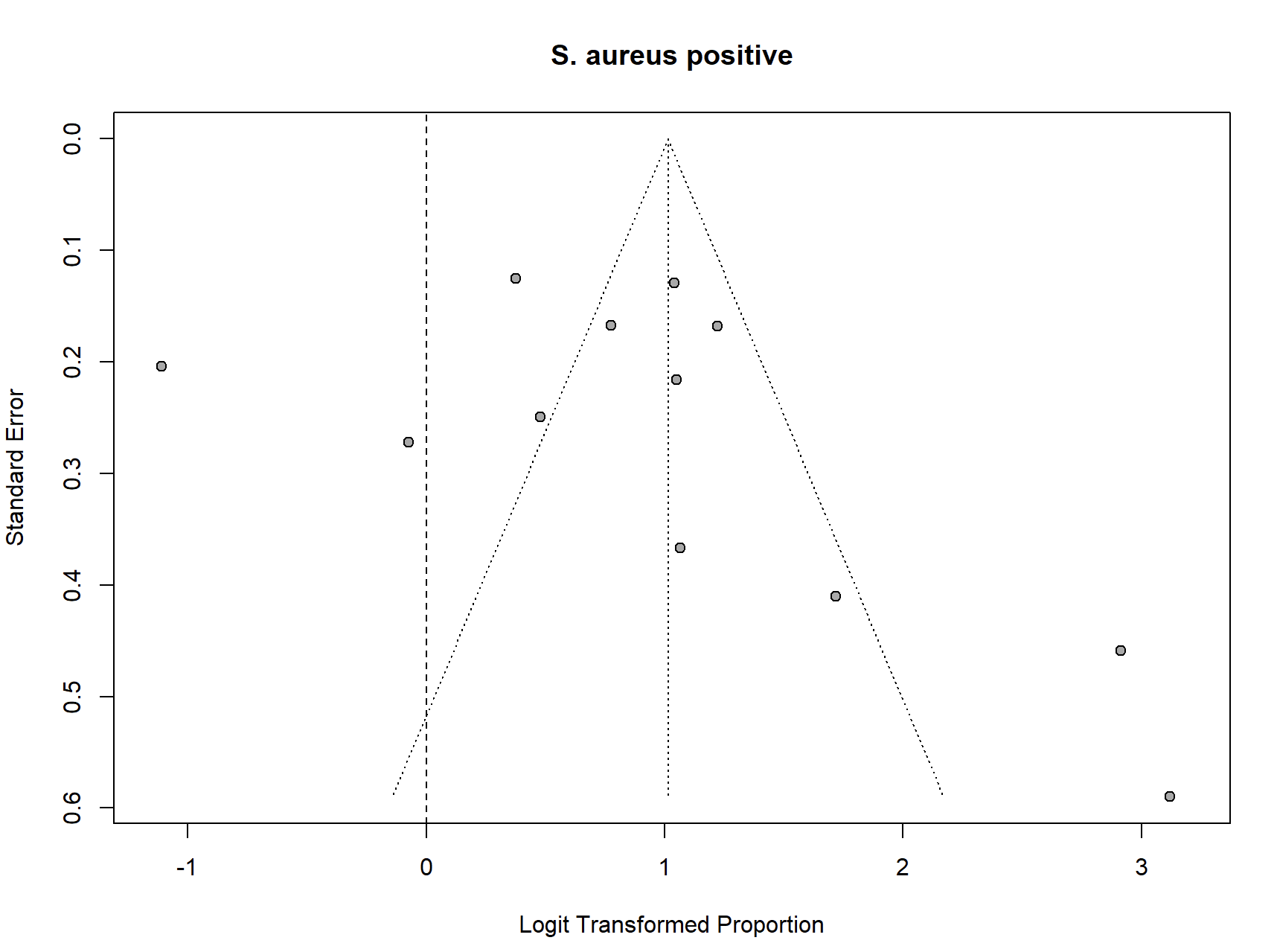


**Figure 21.** Funnel plot of logit-transformed proportions for S. aureus positive

**MRSA positive**


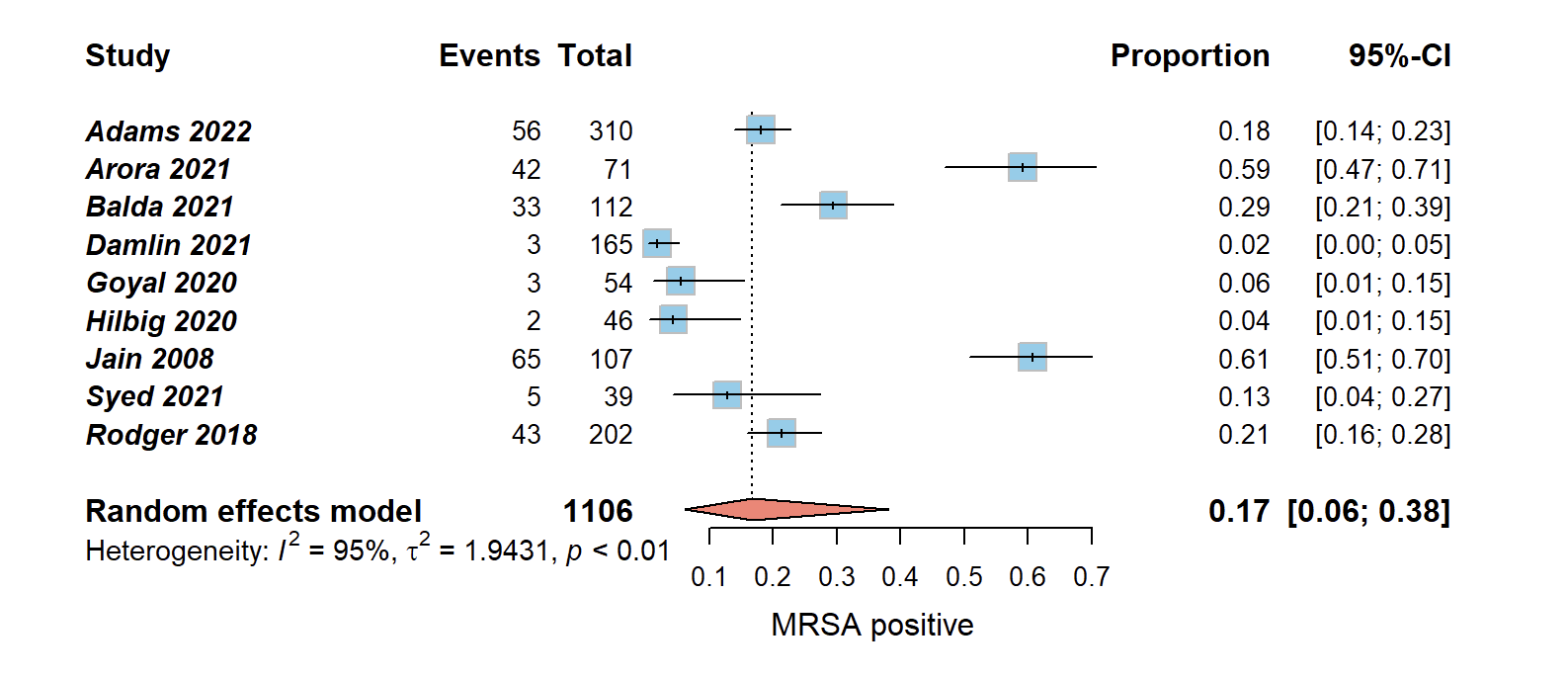


**Figure 22.** Forest plot: Prevalence of MRSA in IVDU-associated IE


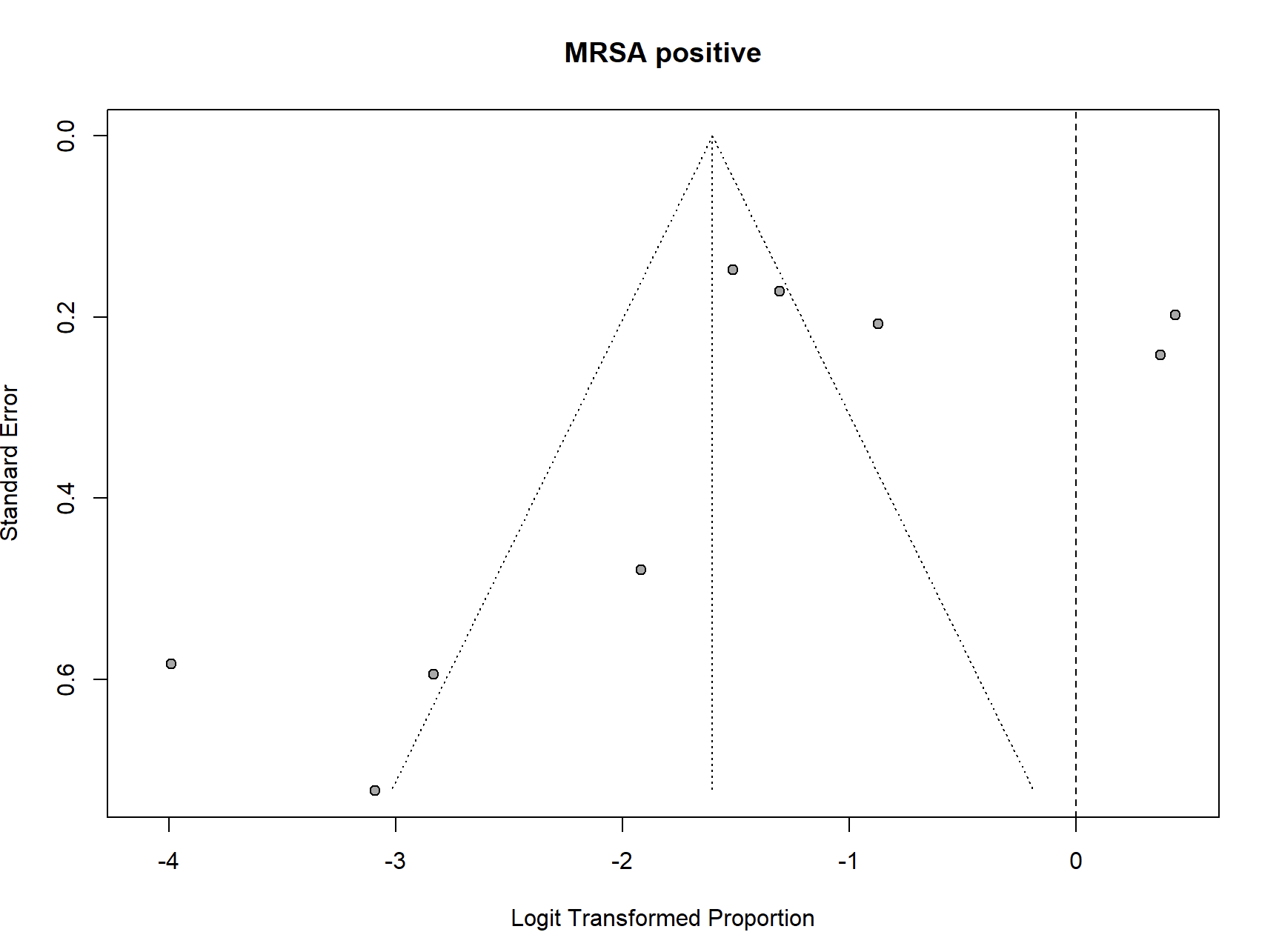


**Figure 23.** Funnel plot: MRSA positive

**MSSA positive**


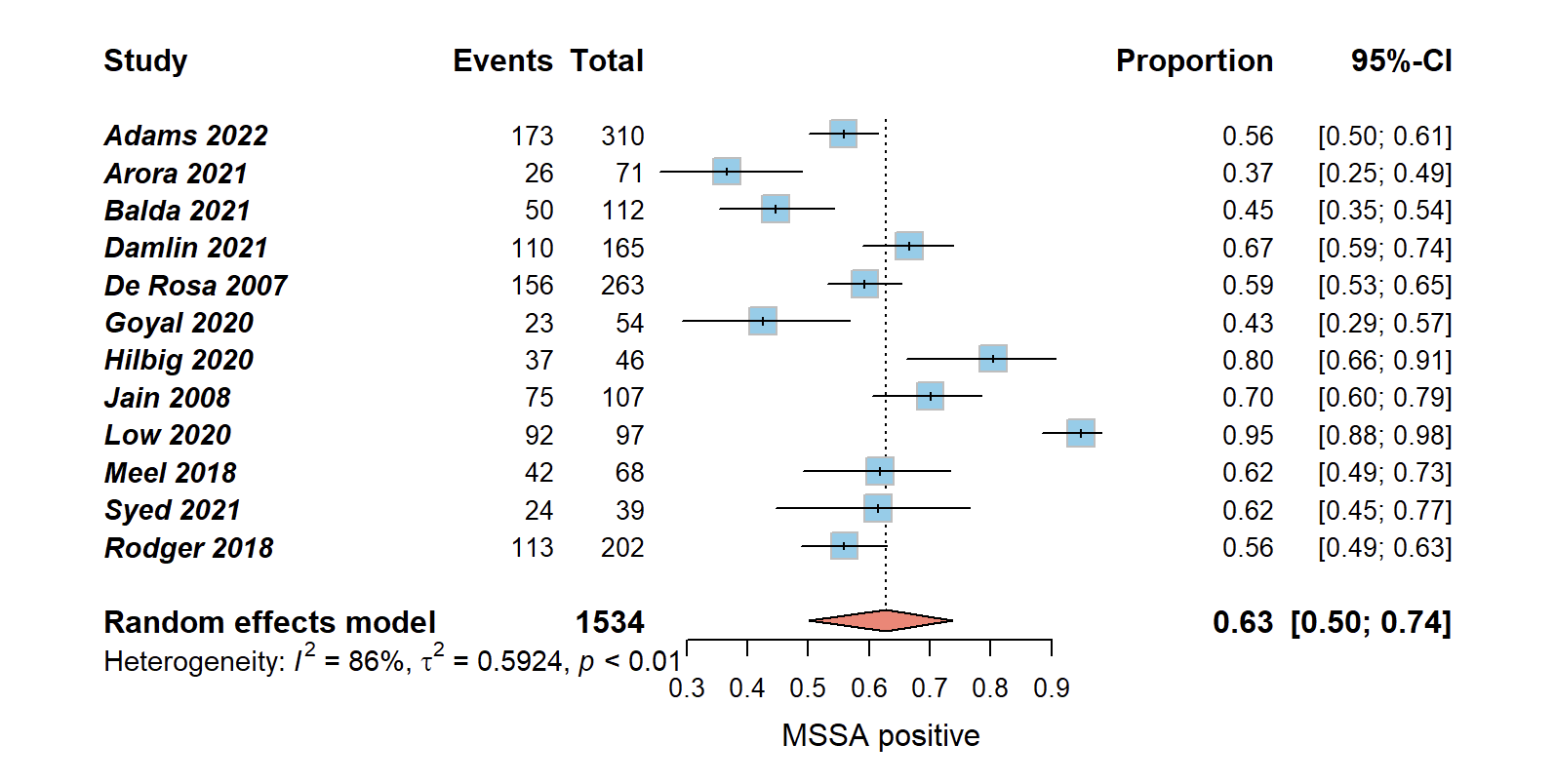


**Figure 24.** Forest plot: Prevalence of MSSA in IVDU-associated IE


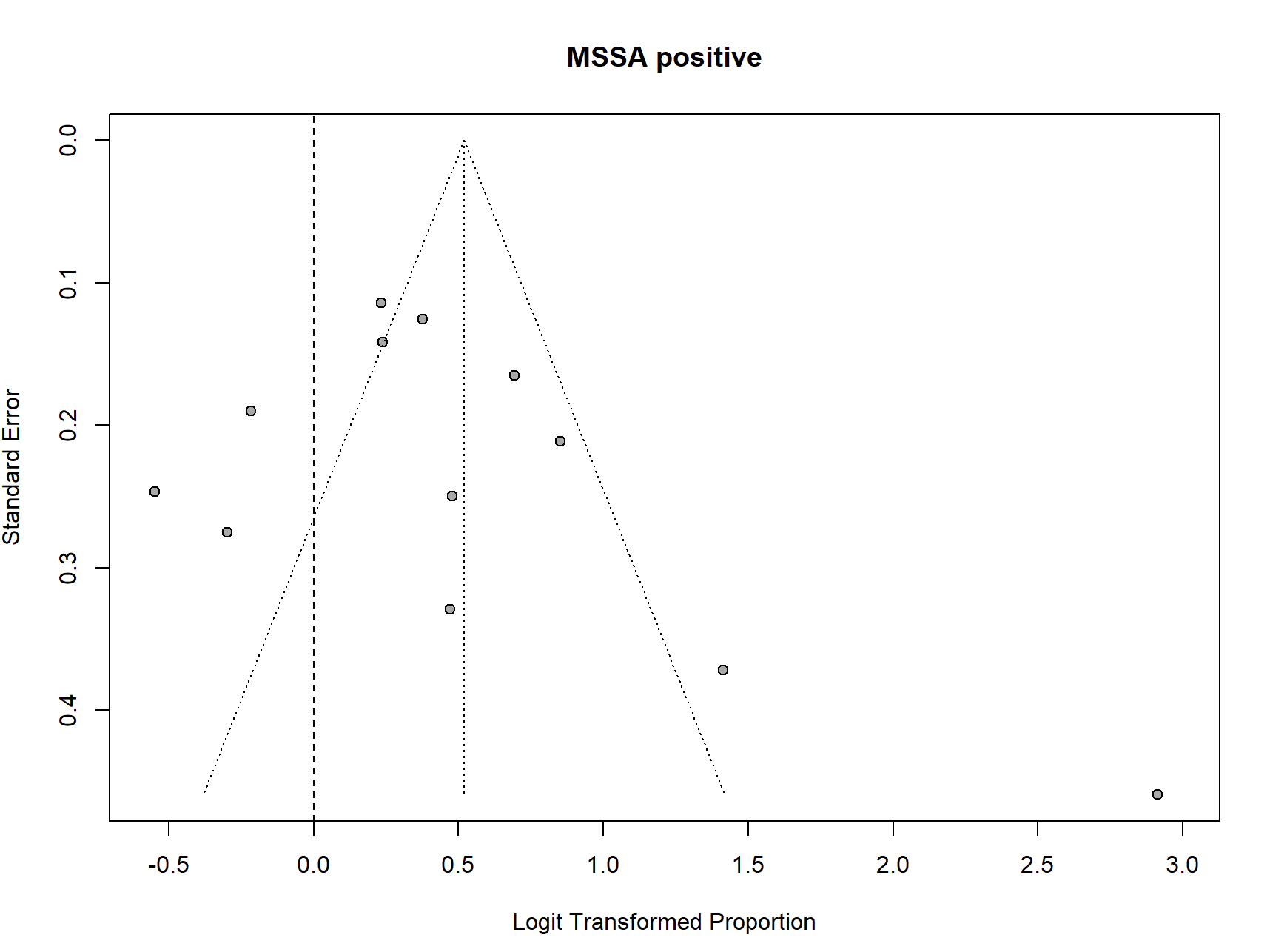


**Figure 25.** Funnel plot: MSSA positive

**Non-viridans Streptococci positivity**


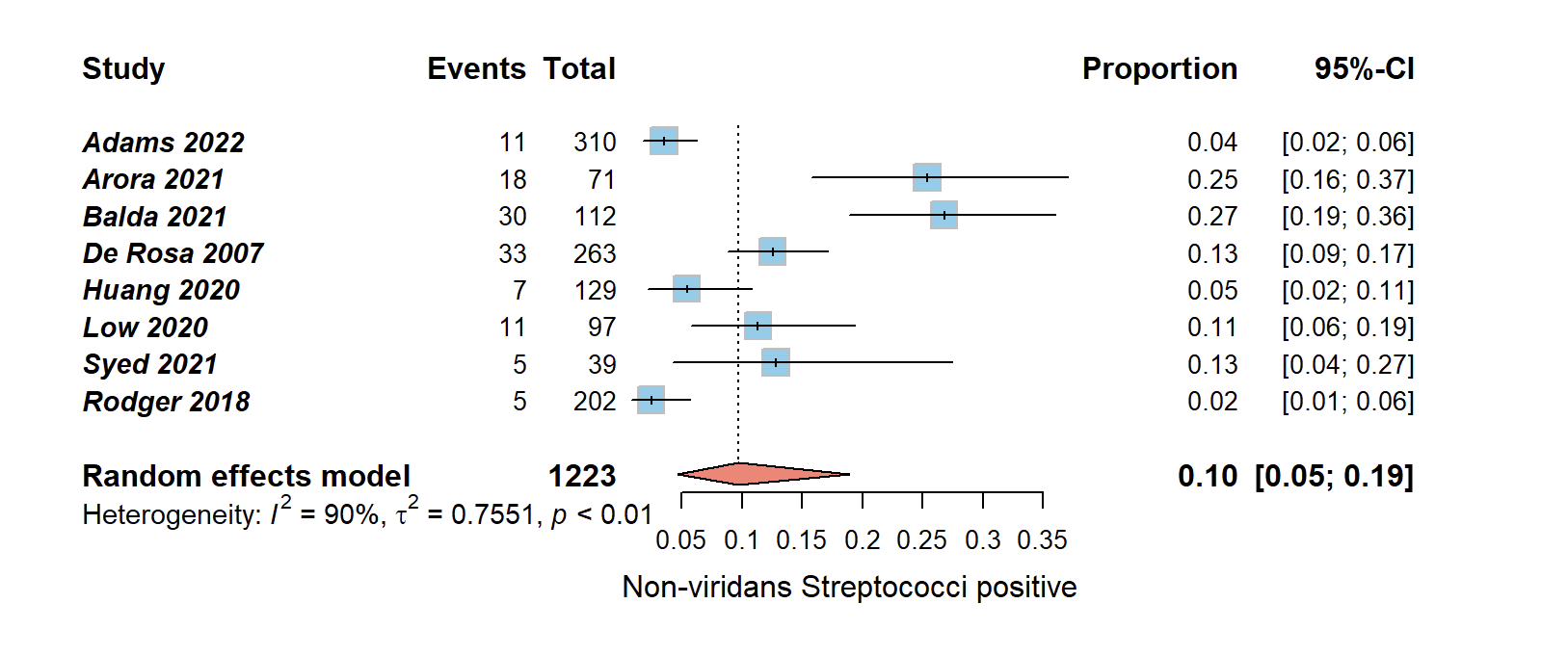


**Figure 26.** Forest plot: Prevalence of non-viridans Streptococci in IVDU-associated IE


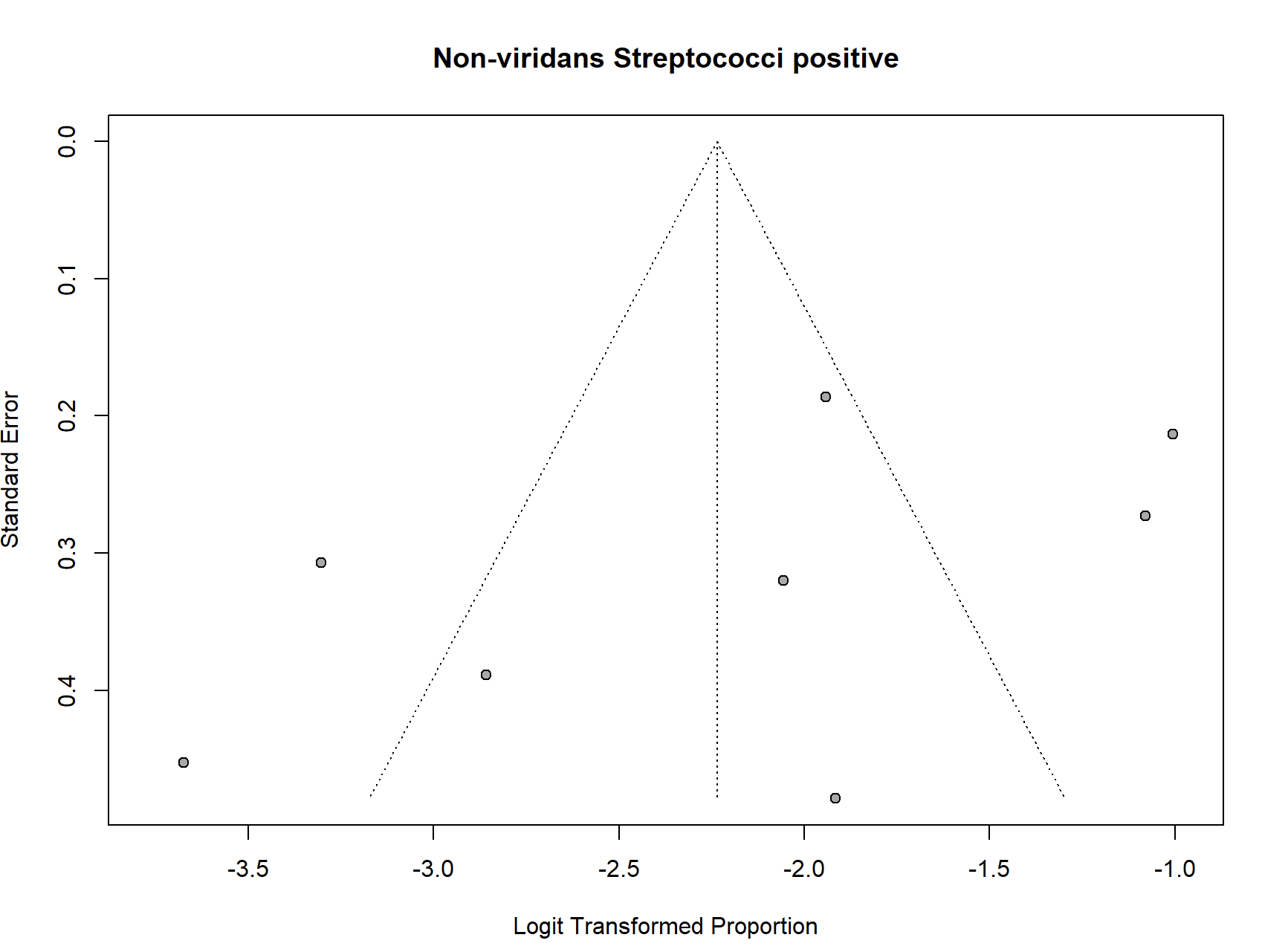


**Figure 27.** Funnel plot: Non-viridans Streptoccocci positive

**S. viridans positivity**


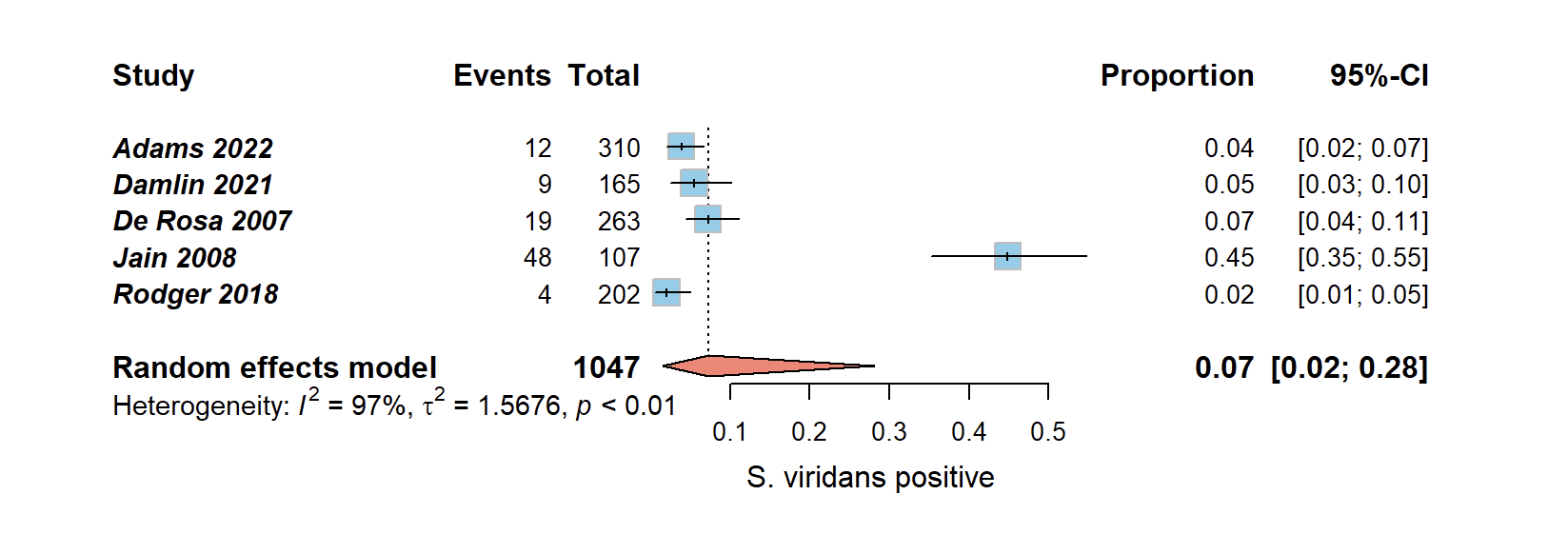


**Figure 28.** Forest plot: Prevalence of S. viridans in IVDU-associated IE


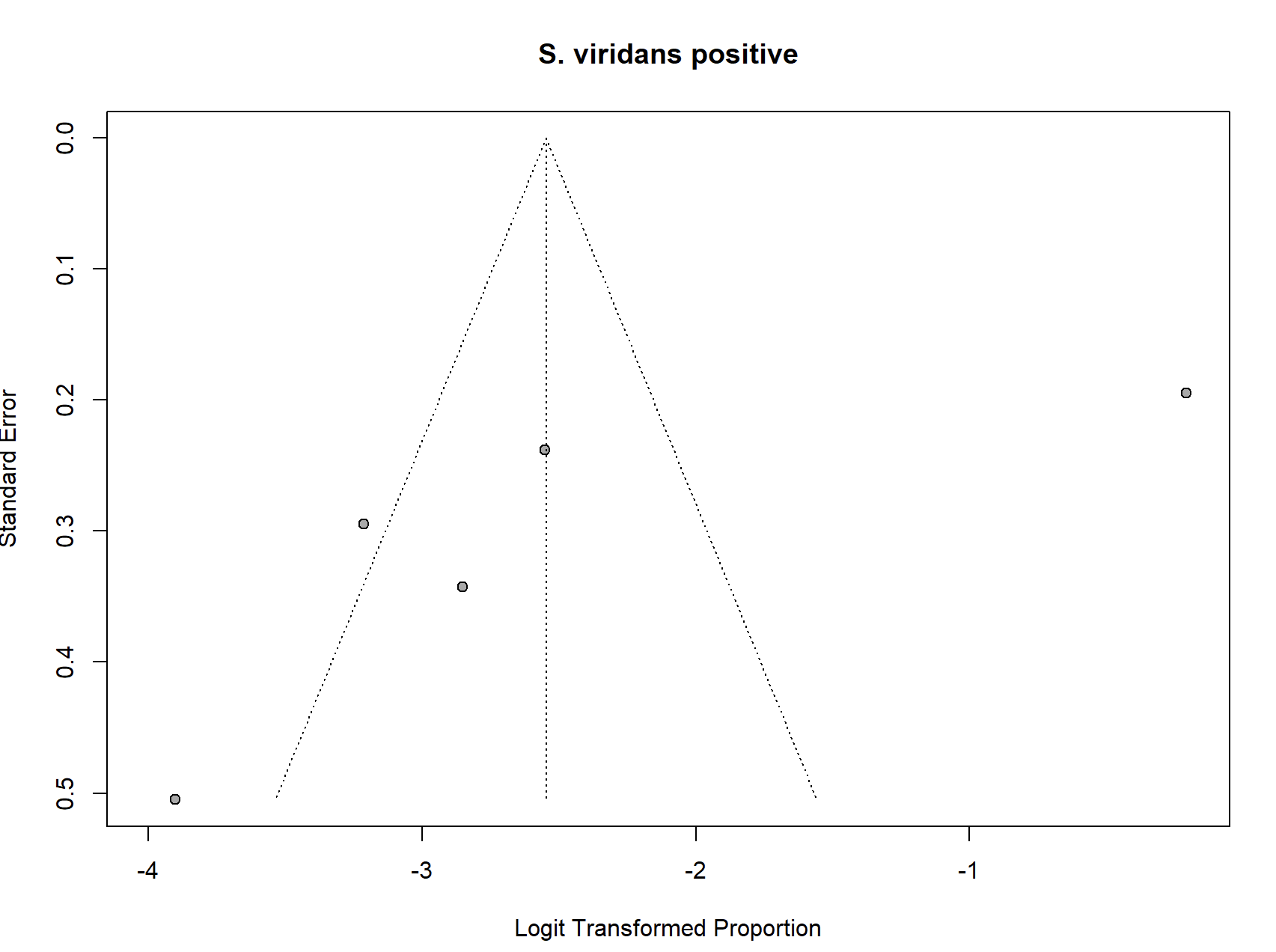


**Figure 29.** Funnel plot: S. viridans positive

**Valve surgeries**


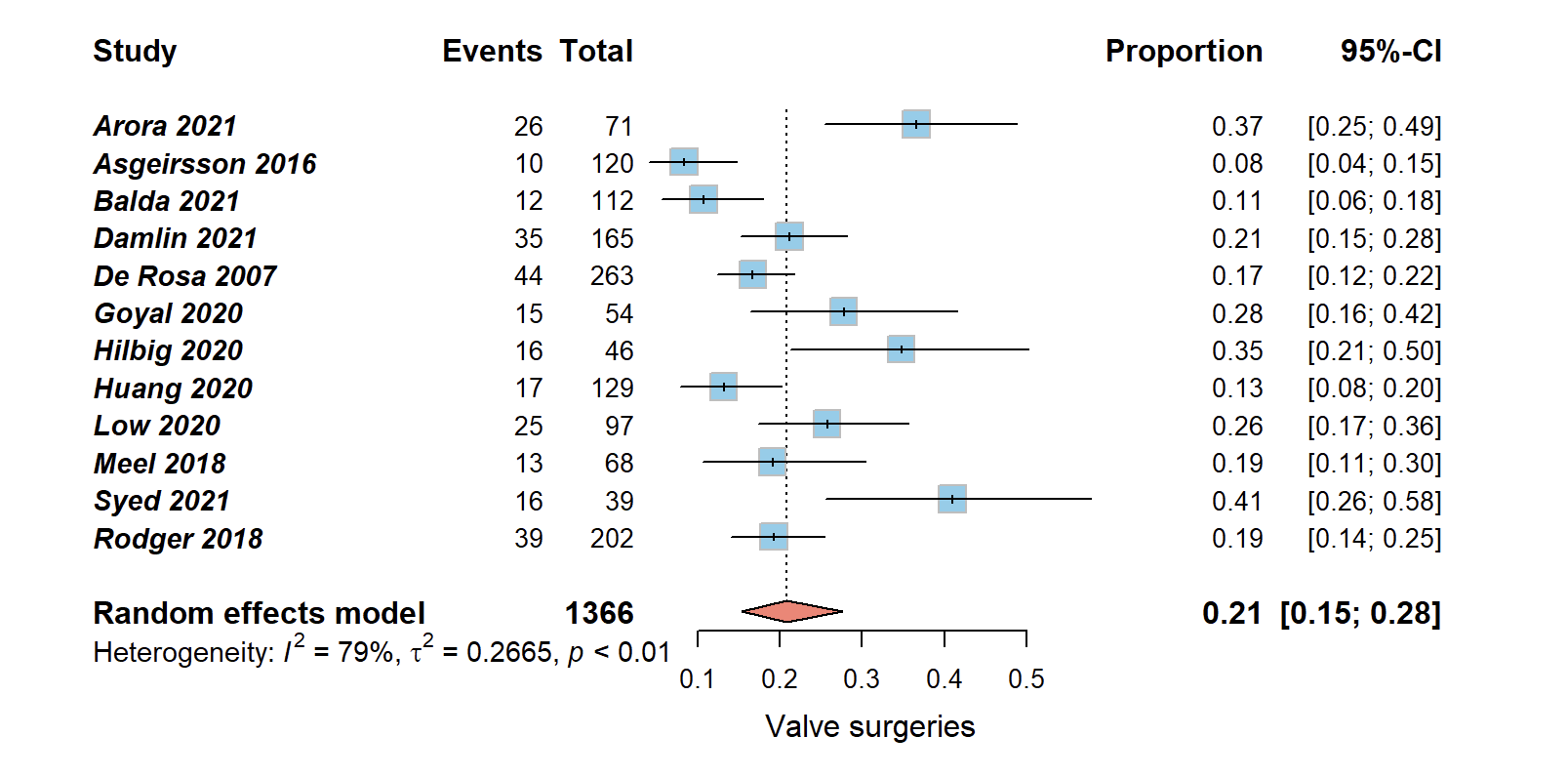


**Figure 30.** Forest plot: Pooled prevalence of valve surgeries in IVDU-associated IE


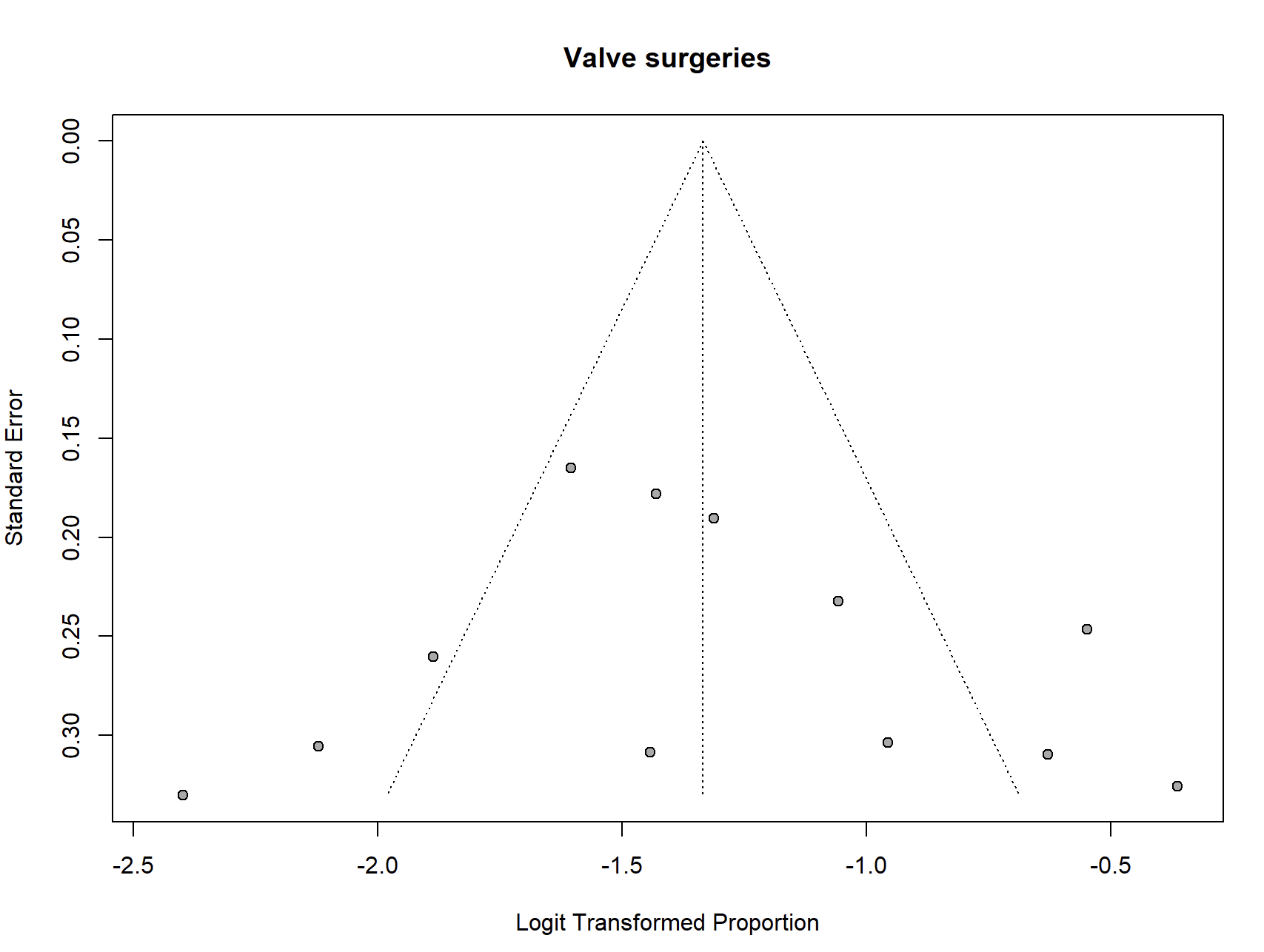


**Figure 31.** Funnel plot: Valve surgeries

**All cause mortality**


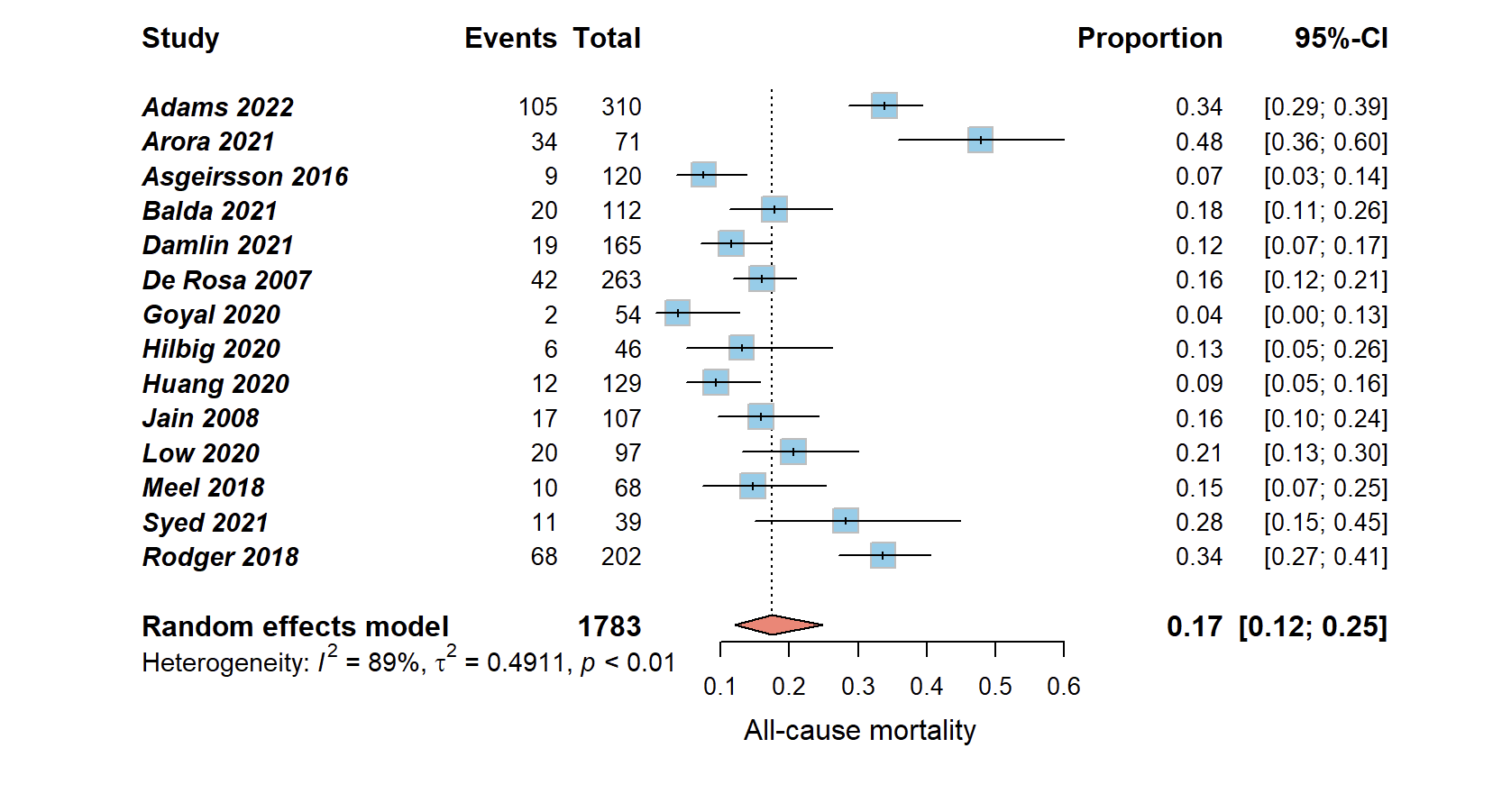


**Figure 32.** Forest plot: Pooled all-cause mortality in IVDU-associated IE


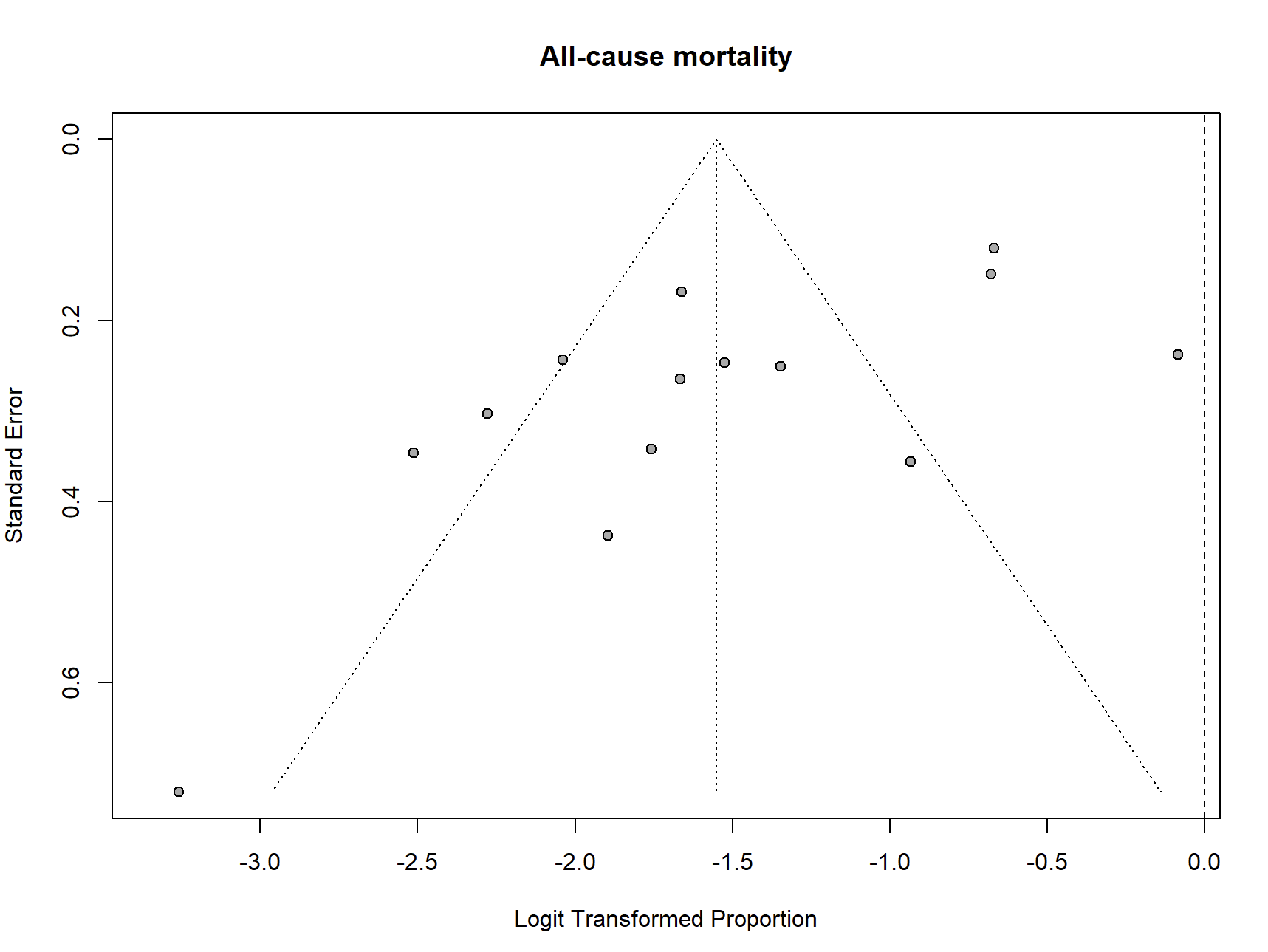


**Figure 33.** Funnel plot: All-cause mortality

# **Sensitivity analysis**

Pulmonic valve involvement remained rare among IVDU-associated IE cases, with a pooled prevalence of 2% (95% CI: 1%–3%) under a random-effects model. Estimates were consistently low across all studies, with narrow confidence intervals. This reinforces the clinical observation that pulmonic valve IE is exceedingly uncommon, even in high-risk populations.

*
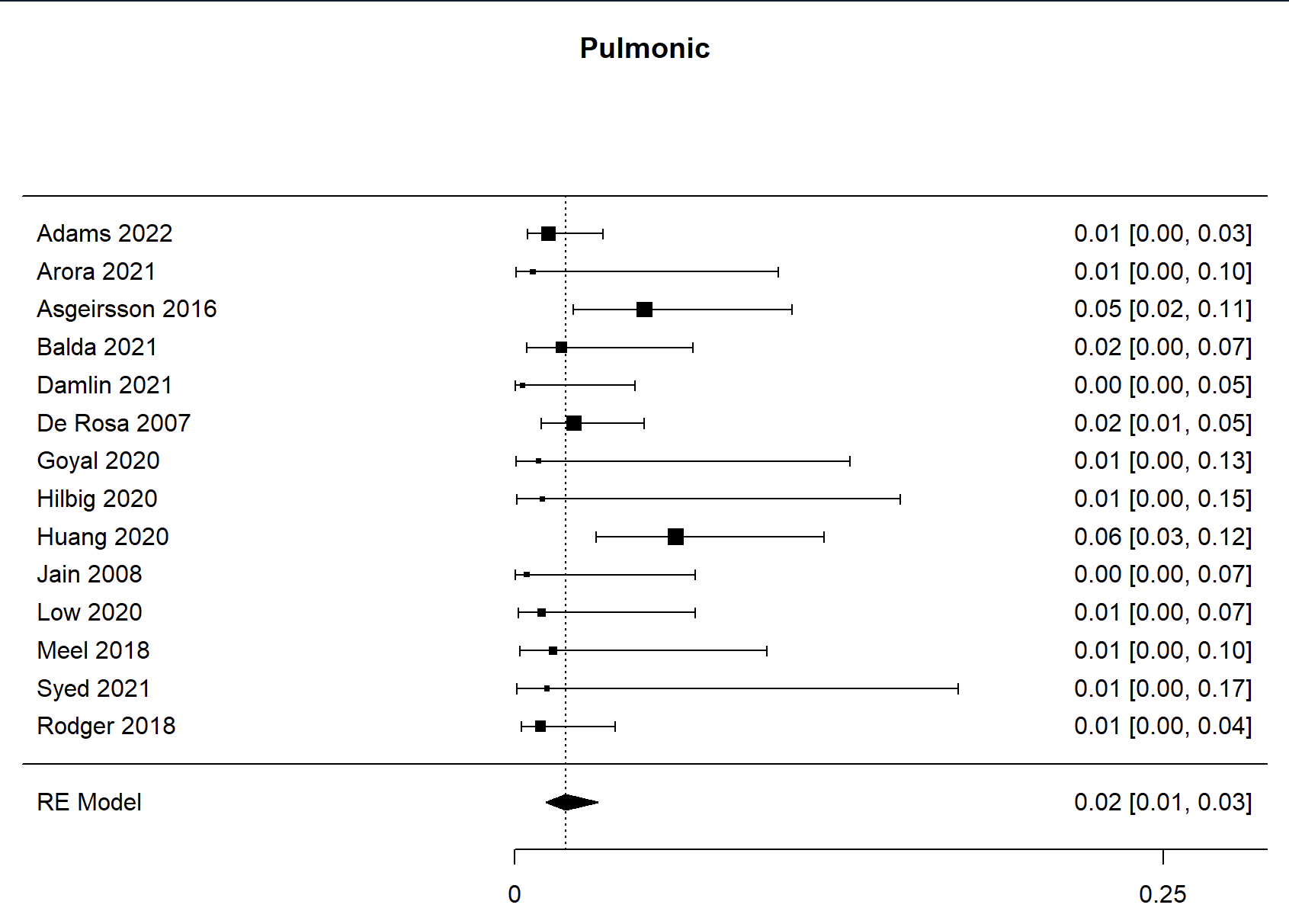
*

**Figure 34.** Sensitivity analysis: Pooled prevalence of pulmonic valve involvement

**Baujat plot: Pulmonic**

The Baujat plot for pulmonic valve involvement identified Huang 2020 and Asgeirsson 2016 as having the greatest influence on overall heterogeneity. These studies contributed disproportionately to both the Pearson residual and the influence on the overall pooled effect. Excluding them may be considered in sensitivity analyses to assess result robustness.

*
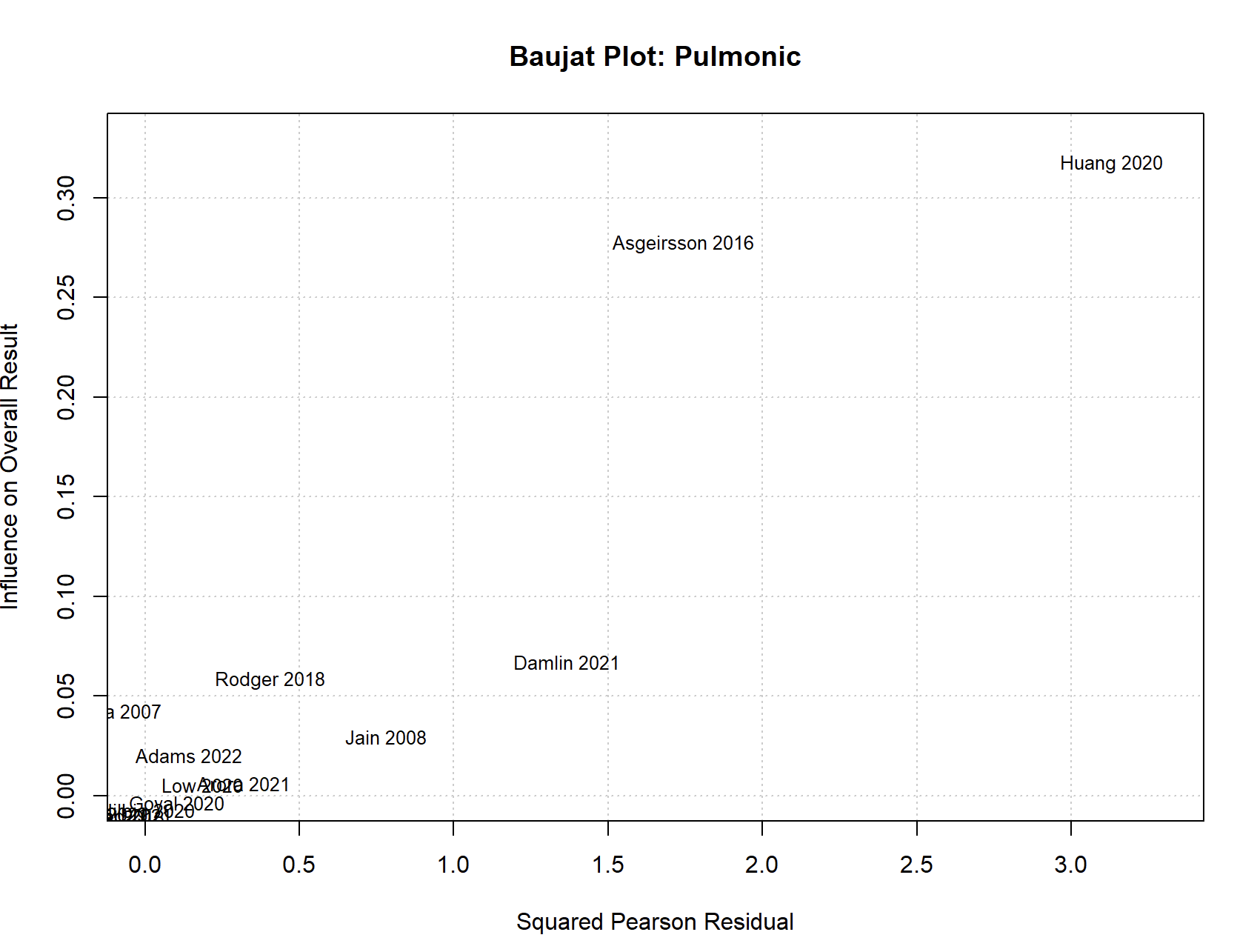
*

**Figure 35.** Baujat plot: Study contributions to heterogeneity in pulmonic valve involvement

**Influence diagnostics: Pulmonic**

Influence diagnostics for pulmonic valve involvement indicated that most studies had minimal impact on overall model estimates. However, one or two studies (notably study 12) showed elevated values across multiple indices (e.g., Cook’s distance, dffits, tau².del, and QE.del), suggesting potential outlier influence. These findings support conducting sensitivity analyses to determine the robustness of pooled results.

*
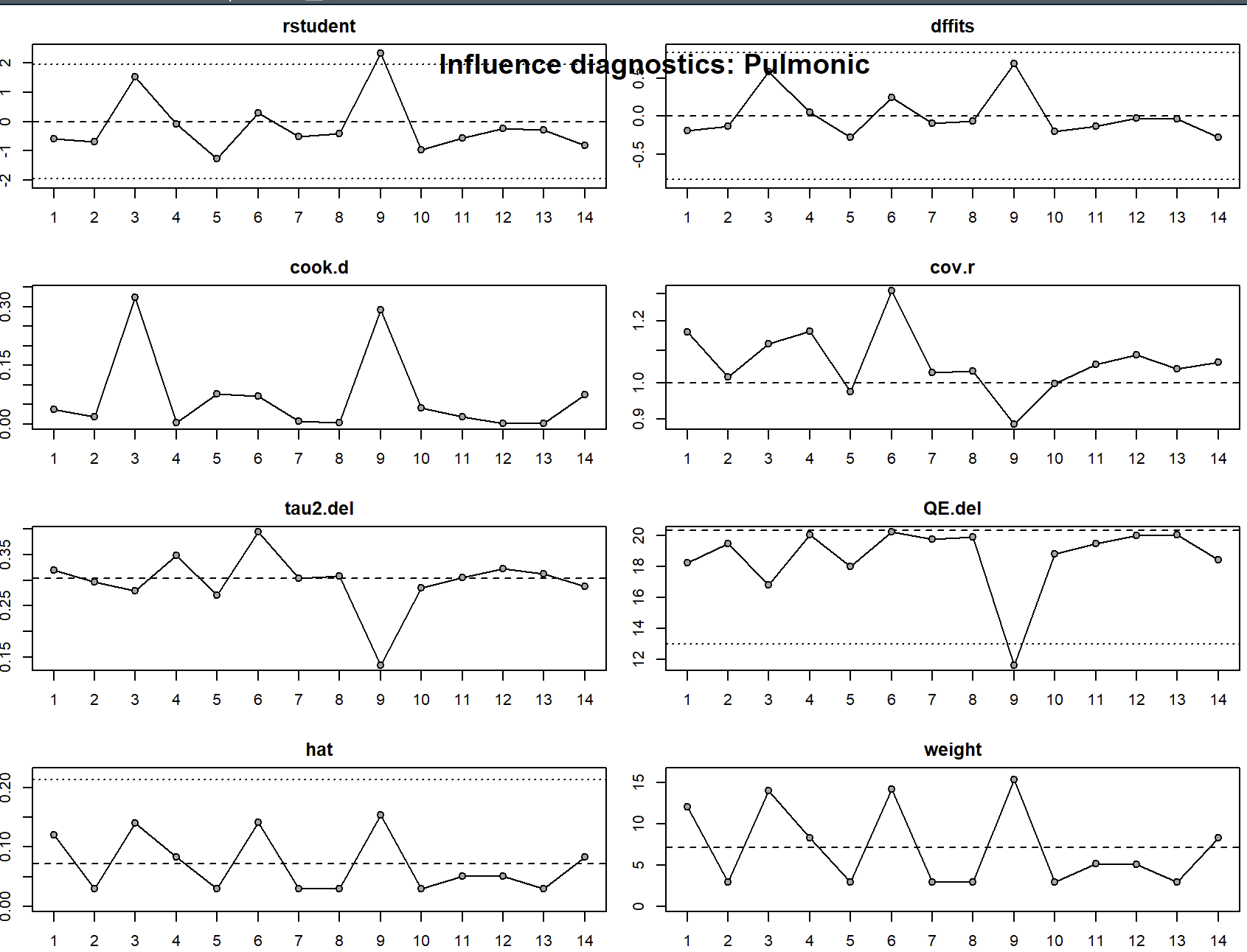
*

**Figure 36.** Influence diagnostics: Pulmonic valve involvement

**Leave-one-out sensitivity: Pulmonic**

Leave-one-out sensitivity analysis for pulmonic valve involvement demonstrated that no single study significantly altered the pooled prevalence estimate. All point estimates and confidence intervals remained stable when each study was individually excluded. This supports the robustness of the overall pooled proportion of 2%, indicating the findings are not driven by outlier studies.

*
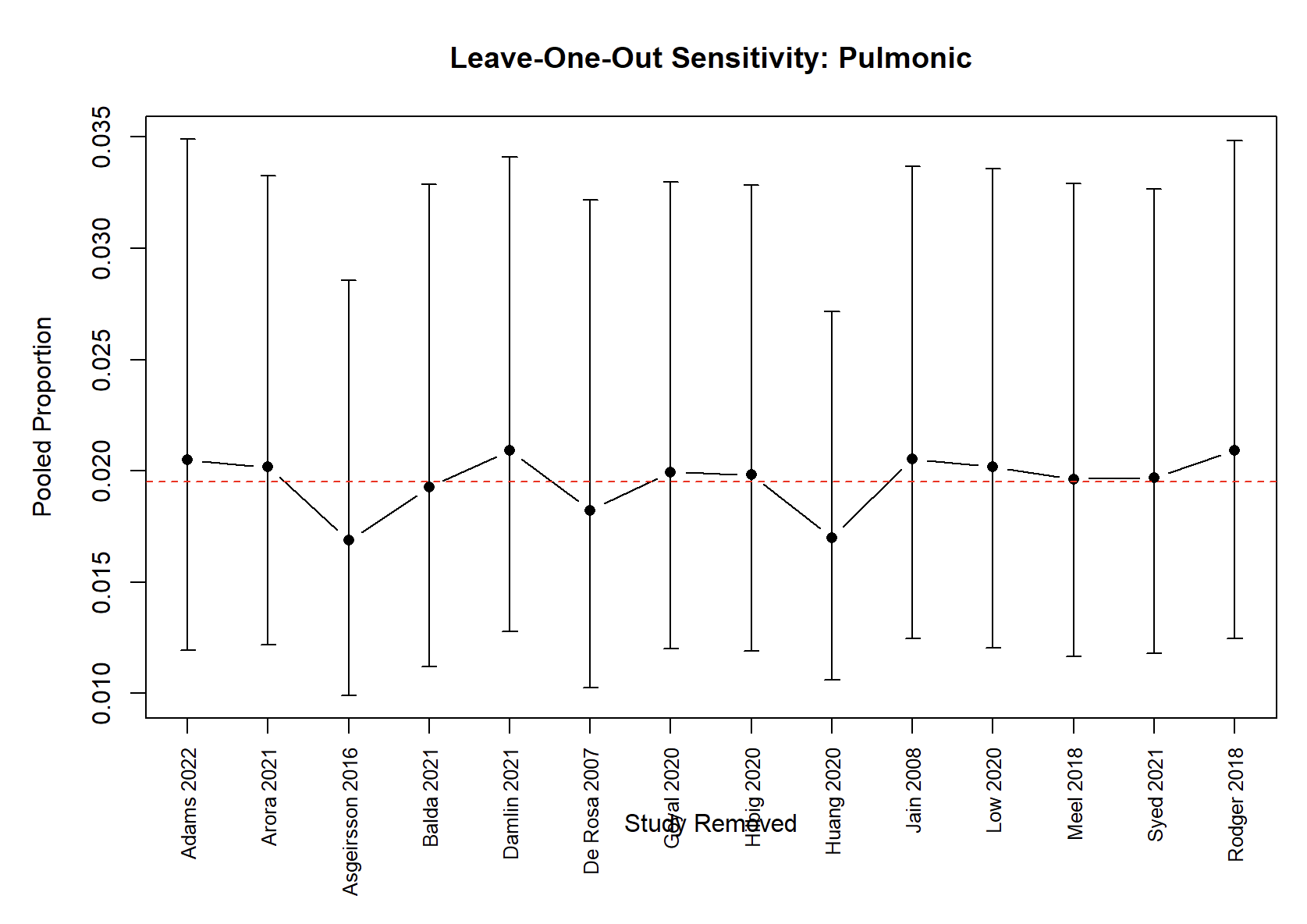
*

**Figure 37.** Leave-one-out analysis: Pulmonic valve involvement

**Tricuspid valve forest plot**

The pooled prevalence of tricuspid valve involvement in IVDU-associated IE was 59% (95% CI: 54%–63%) based on data from 13 studies. Most individual study estimates ranged from 44% to 68%, with relatively consistent results across cohorts. The narrow confidence interval around the pooled estimate and visual alignment of studies support the reliability of this finding, confirming tricuspid valve as the most commonly affected site in this population.

*
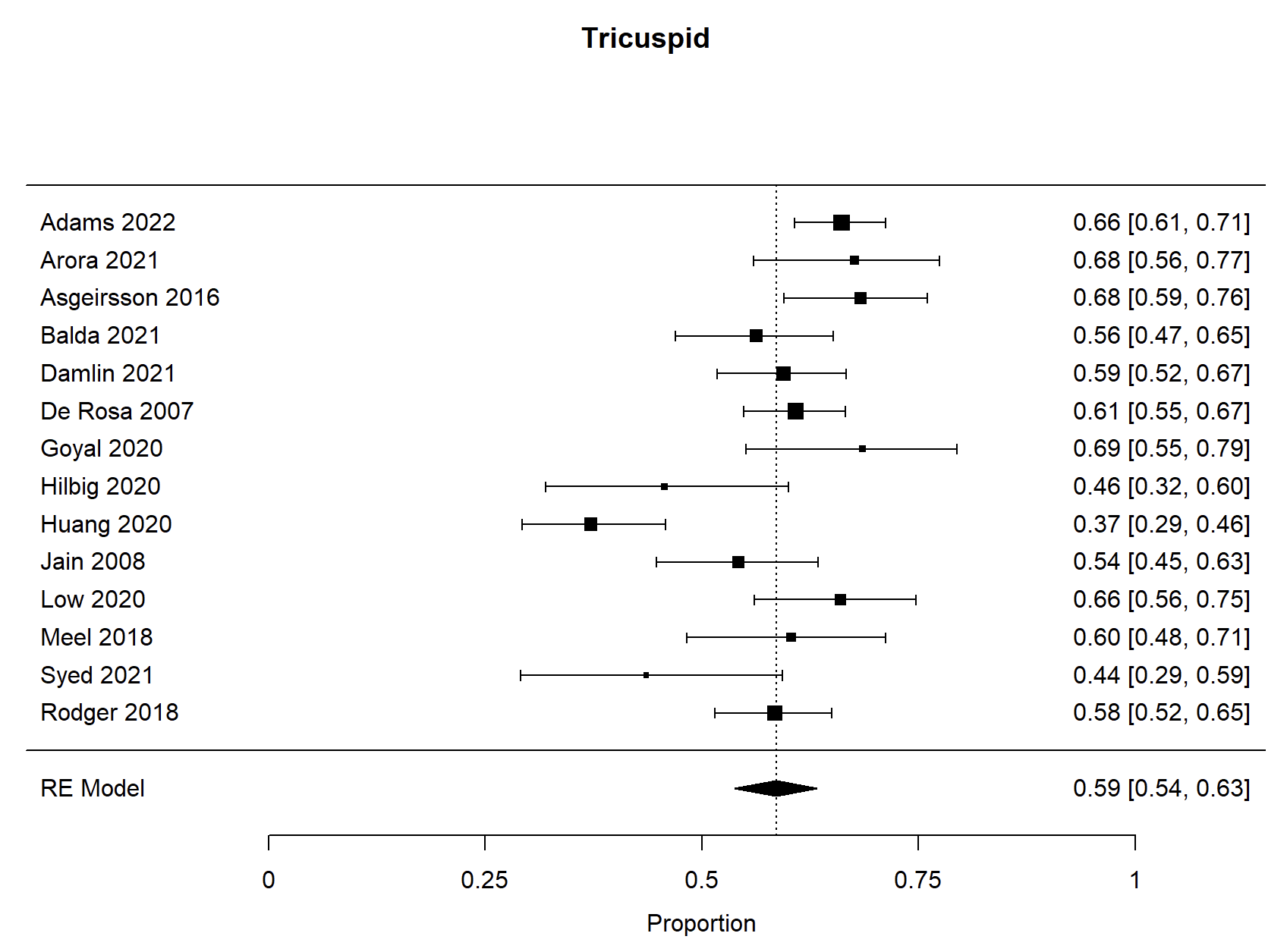
*

**Figure 38.** Forest plot: Pooled prevalence of tricuspid valve involvement

**Baujat plot: Tricuspid**

The Baujat plot for tricuspid valve involvement identified Huang 2020 as the most influential study contributing to heterogeneity, with the highest squared Pearson residual and overall impact on the pooled result. Other studies clustered closely near the origin, indicating minimal individual influence. This suggests that the pooled estimate is generally stable, though sensitivity analysis excluding Huang 2020 may be warranted.

*
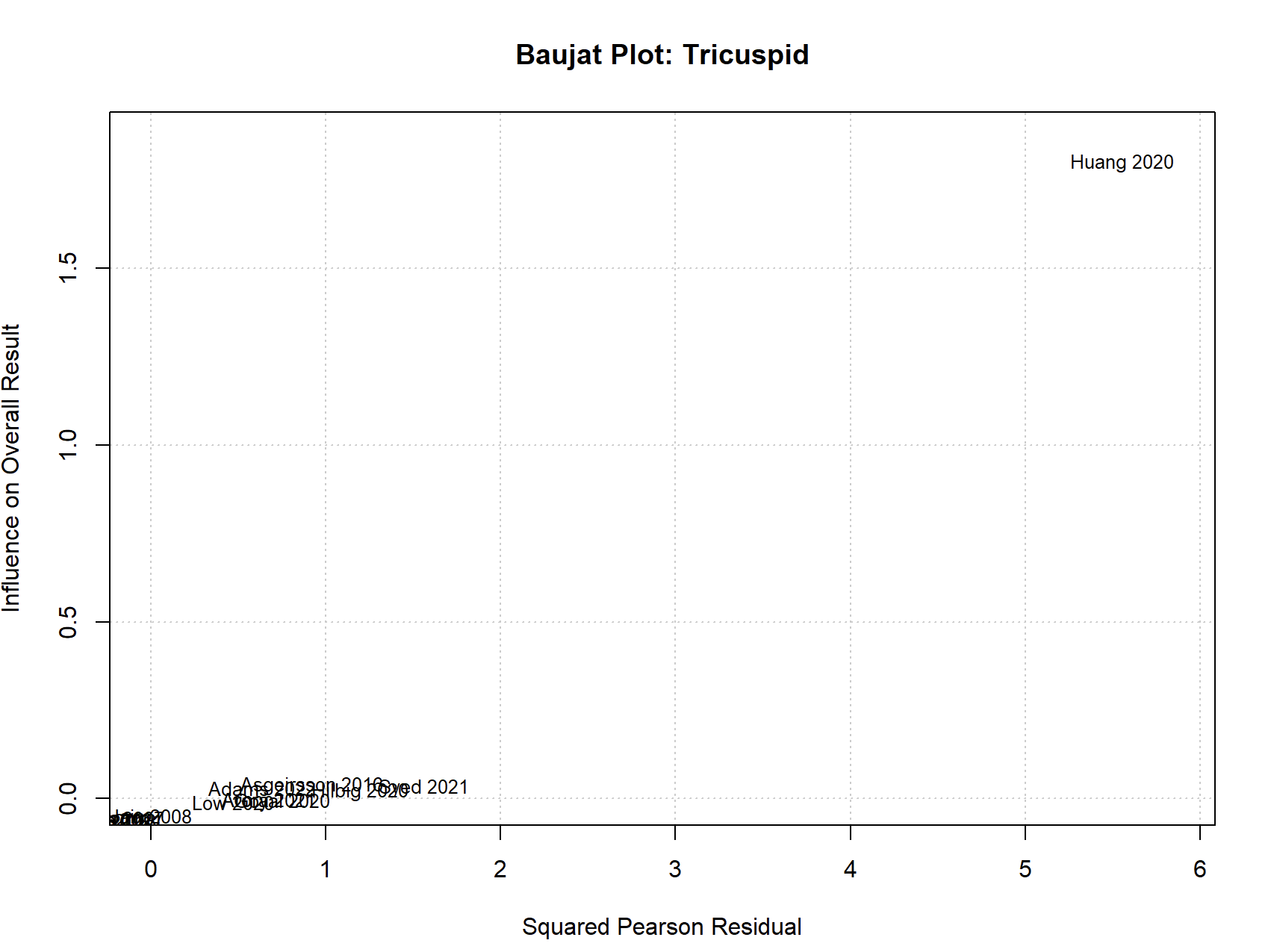
*

**Figure 39.** Baujat plot: Tricuspid valve heterogeneity

**Influence diagnostic: Tricuspid**

Influence diagnostics identified study 9 as a potential outlier, showing high values across multiple metrics including Cook’s distance, rstudent, and tau².del. This study had a notable impact on between-study variance and overall model fit. Excluding it in sensitivity analysis may help evaluate the robustness of the tricuspid valve prevalence estimate.

*
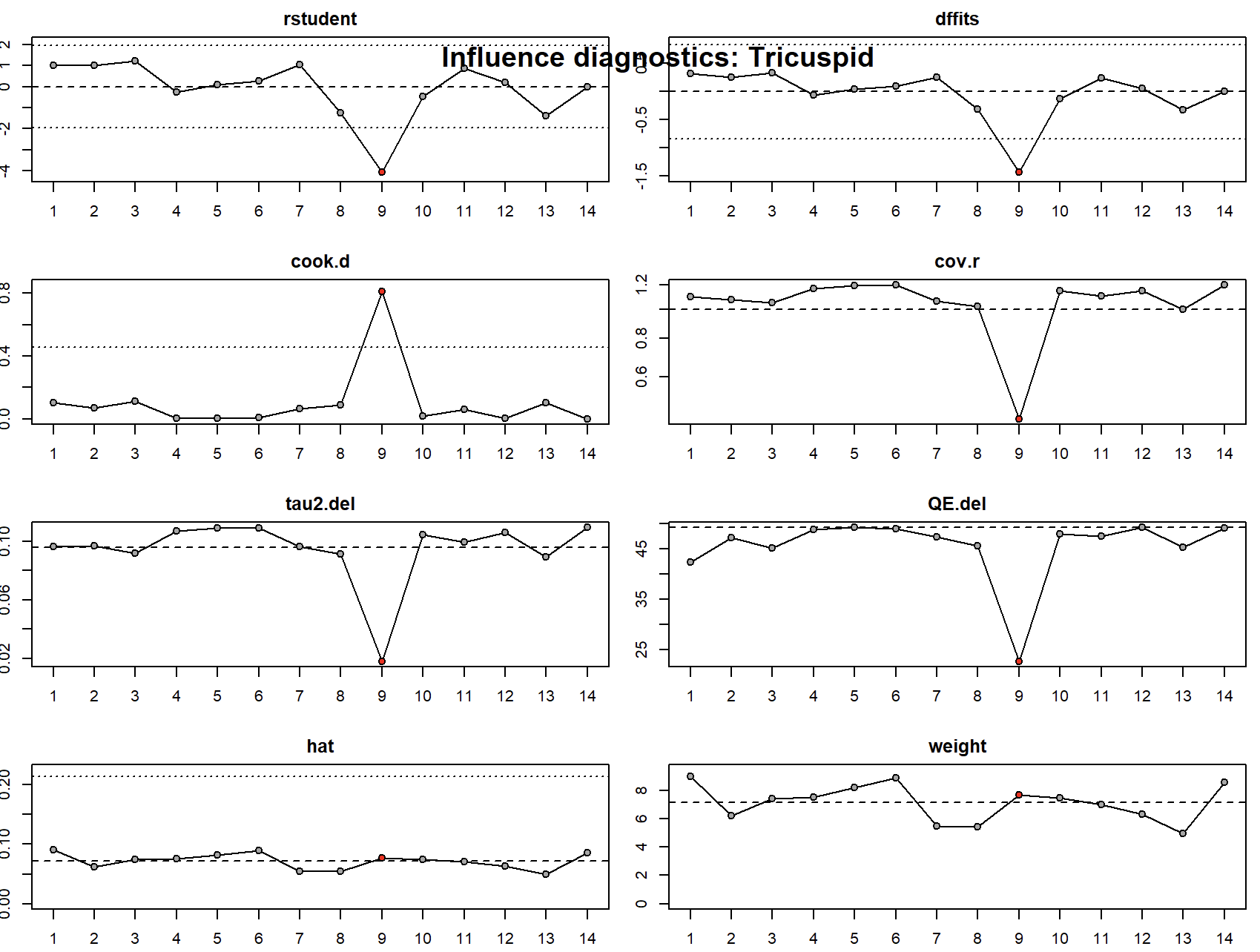
*

**Figure 40.** Influence diagnostics: Tricuspid valve

**Leave-one-out sensitivity – tricuspid valve**

Leave-one-out analysis showed that exclusion of any single study did not substantially alter the pooled prevalence of tricuspid valve involvement. While removal of Huang 2020 slightly increased the estimate, all confidence intervals overlapped and remained within a narrow range. This supports the robustness of the 59% pooled proportion.

*
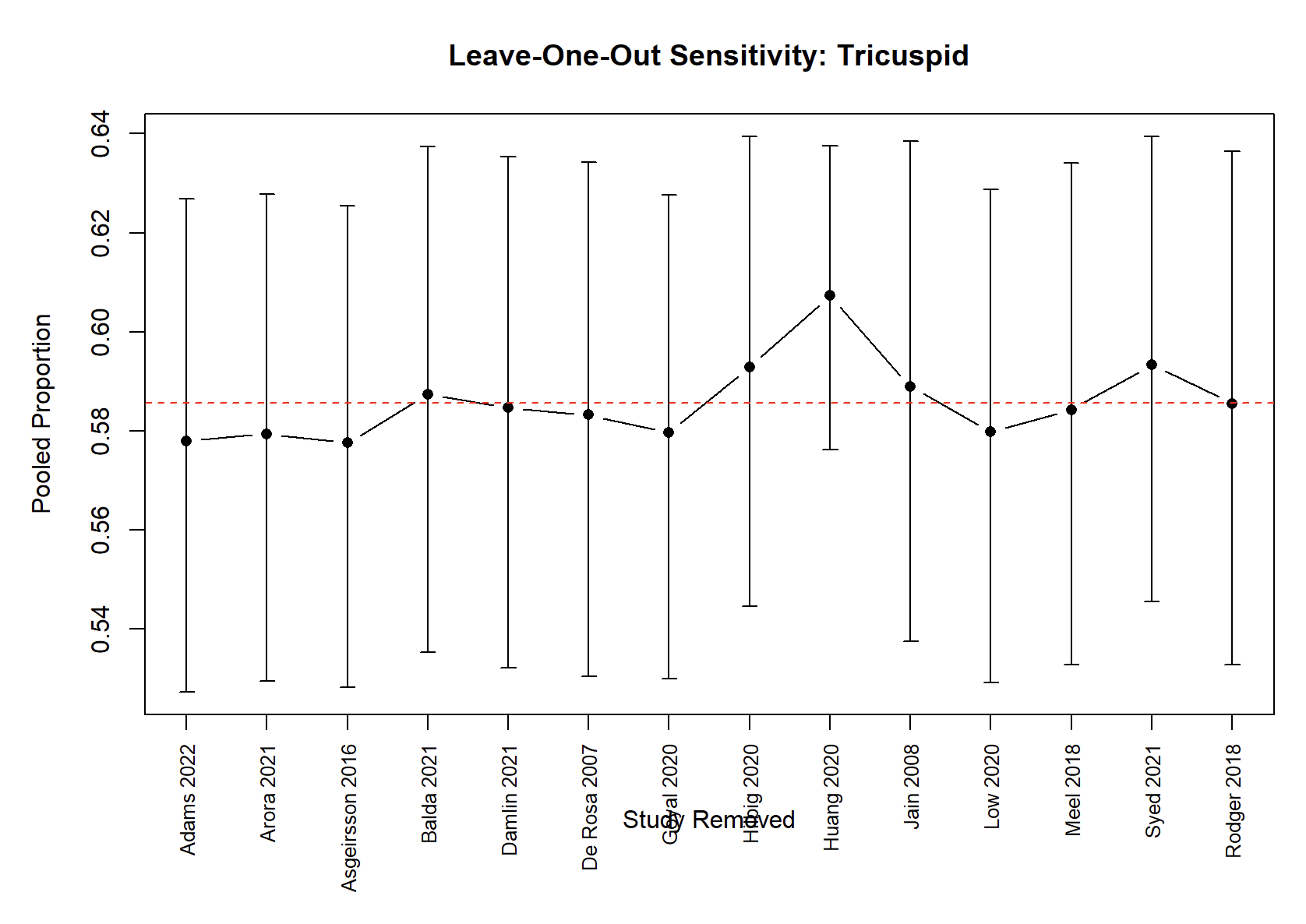
*

**Figure 41.** Leave-one-out analysis: Tricuspid valve

**Mitral valve forest plot**

The pooled prevalence of mitral valve involvement was 22% (95% CI: 19%–25%) across 14 studies. Estimates varied modestly, ranging from 10% to 34%, with overlapping confidence intervals indicating consistency. These findings confirm that mitral valve disease represents a significant portion of left-sided IE among IVDU patients, warranting routine left-heart imaging.

*
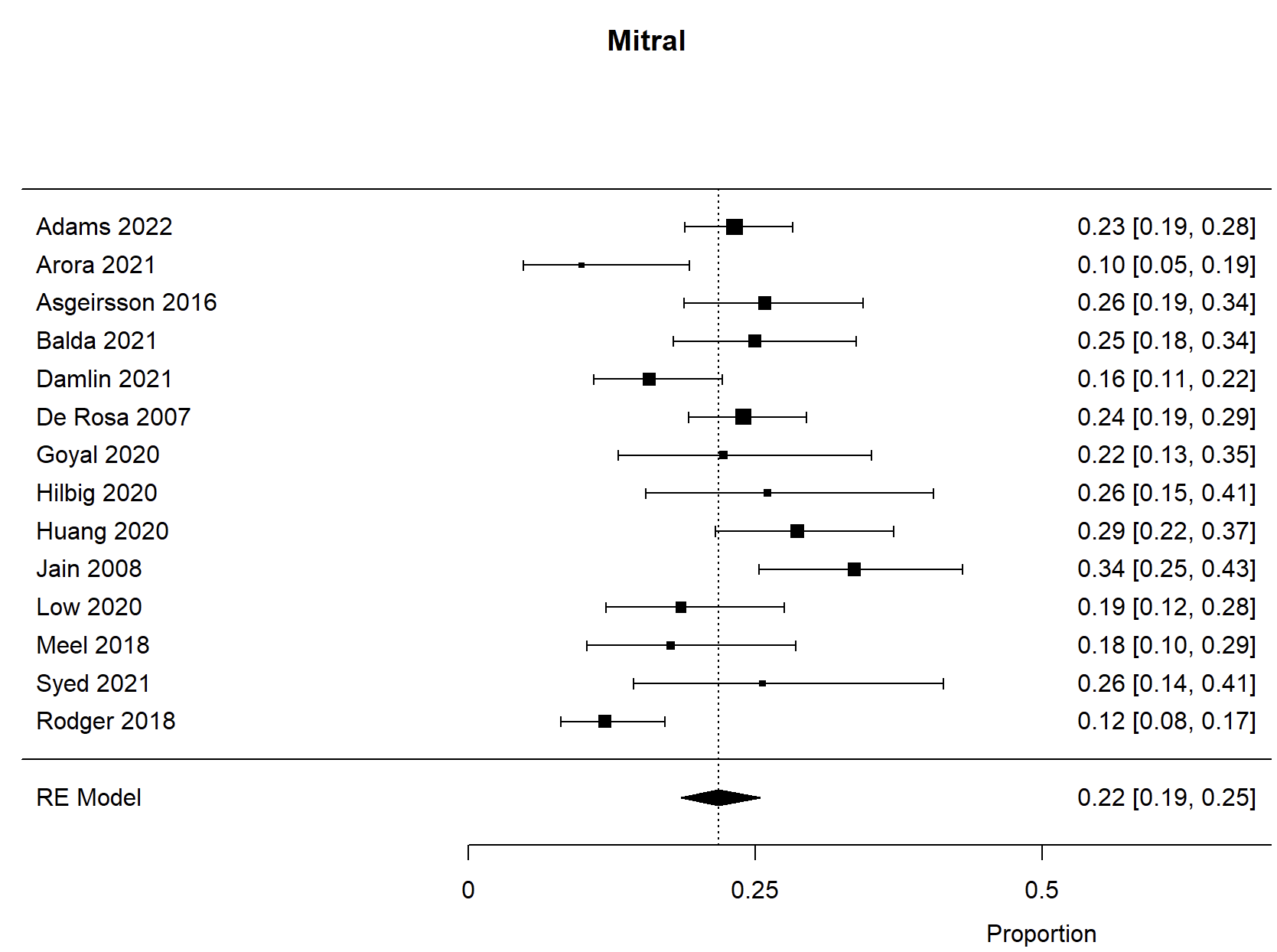
*

**Figure 42.** Forest plot: Mitral valve involvement after excluding influential study

**Baujat plot: Mitral**

The Baujat plot for mitral valve involvement identified Rodger 2018 as the most influential study contributing to heterogeneity. Jain 2008 and Arora 2021 also showed moderate influence, suggesting these studies may warrant closer examination in sensitivity analyses.

*
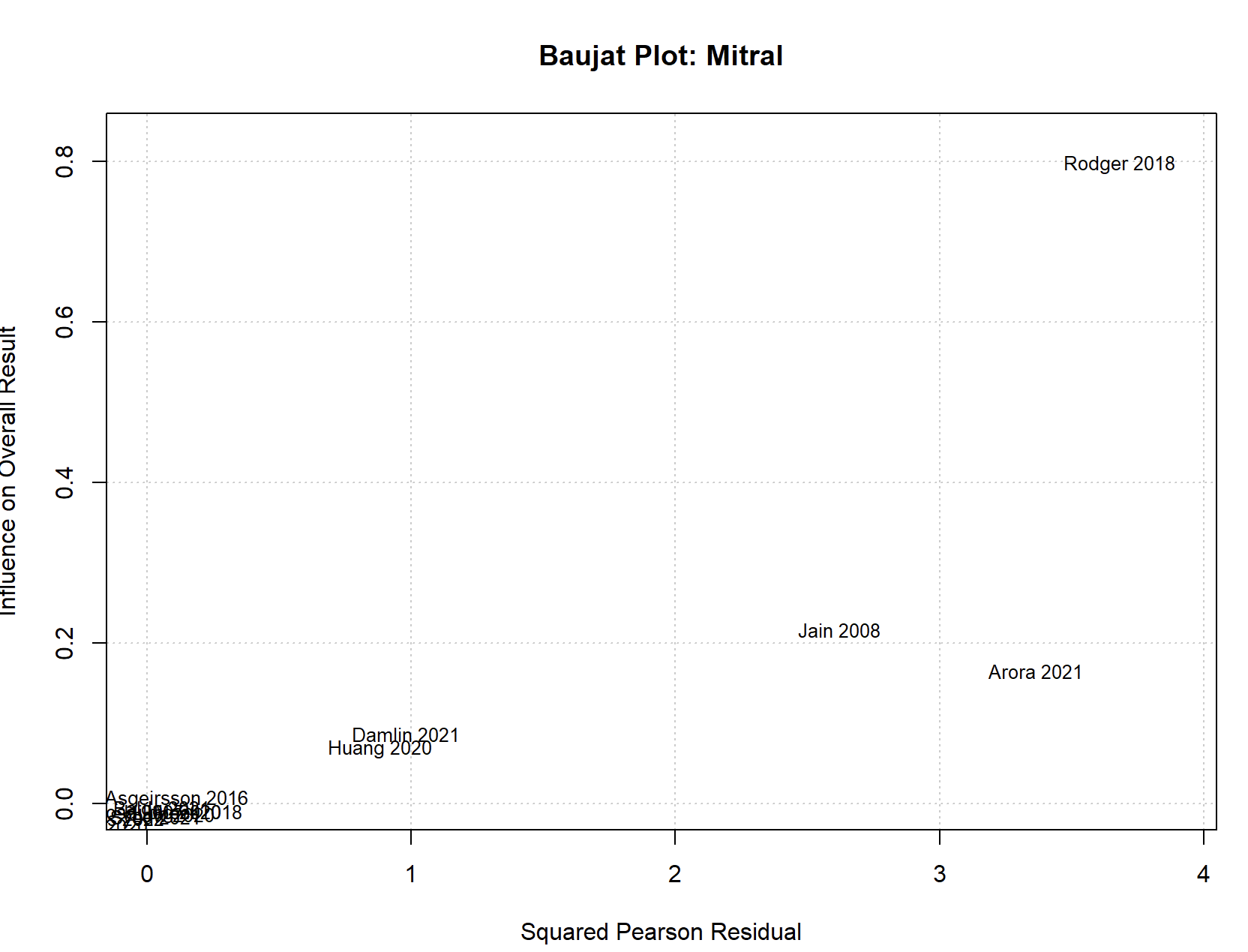
*

**Figure 43.** Baujat plot: Mitral valve heterogeneity

**Influence diagnostics: Mitral**

Influence diagnostics revealed that study 14 (Rodger 2018) had the greatest impact on model estimates across multiple metrics, including Cook’s distance, rstudent, dffits, and tau².del. This suggests it is a potential outlier and may warrant exclusion in sensitivity analyses.

*
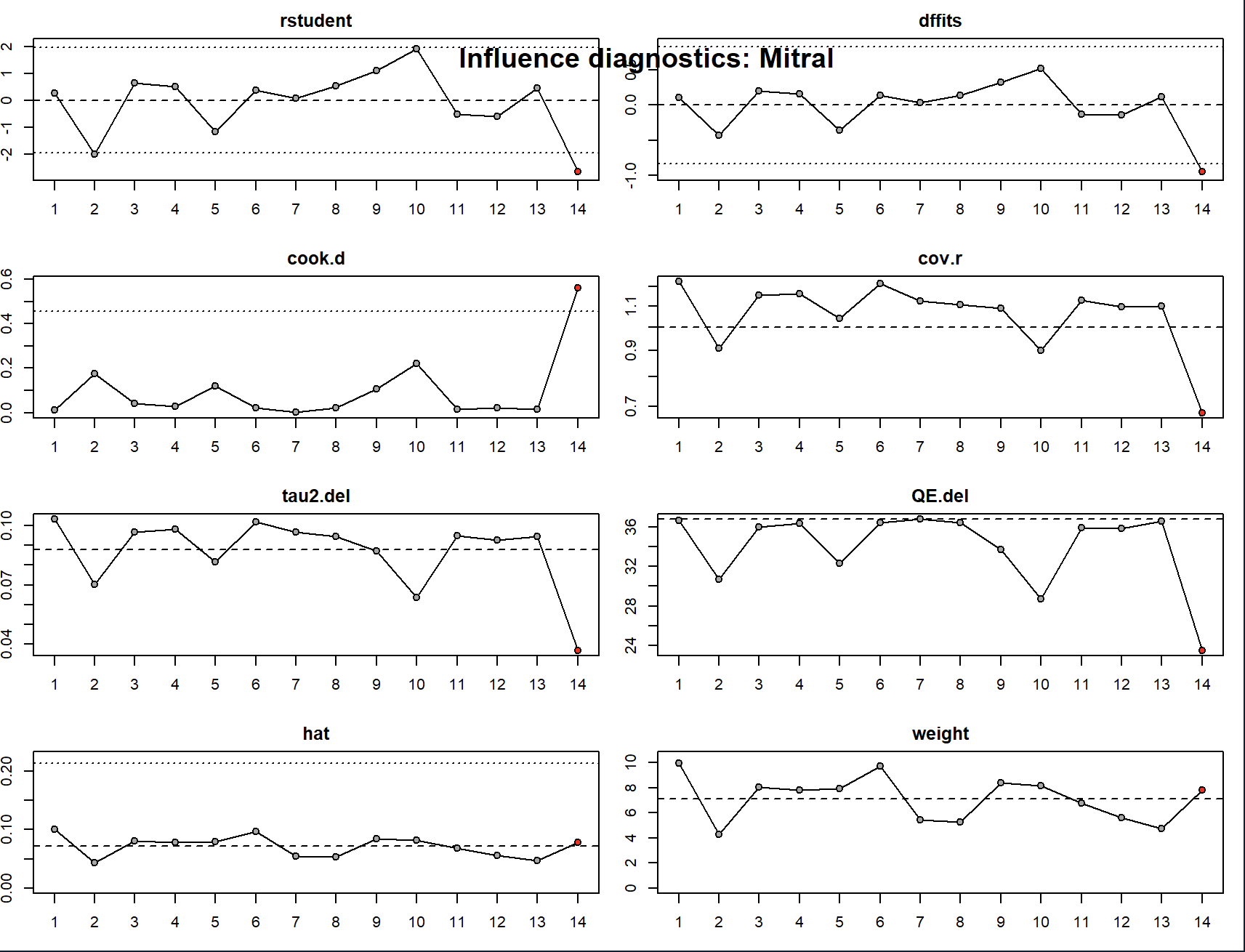
*

**Figure 44.** Influence diagnostics: Mitral valve

**Leave-One-Out Sensitivity: Mitral**

Leave-one-out analysis showed that exclusion of individual studies, including the influential Rodger 2018, did not substantially alter the pooled estimate for mitral valve involvement. All adjusted proportions remained within the 95% CI of the original model, supporting the stability of the findings.

*
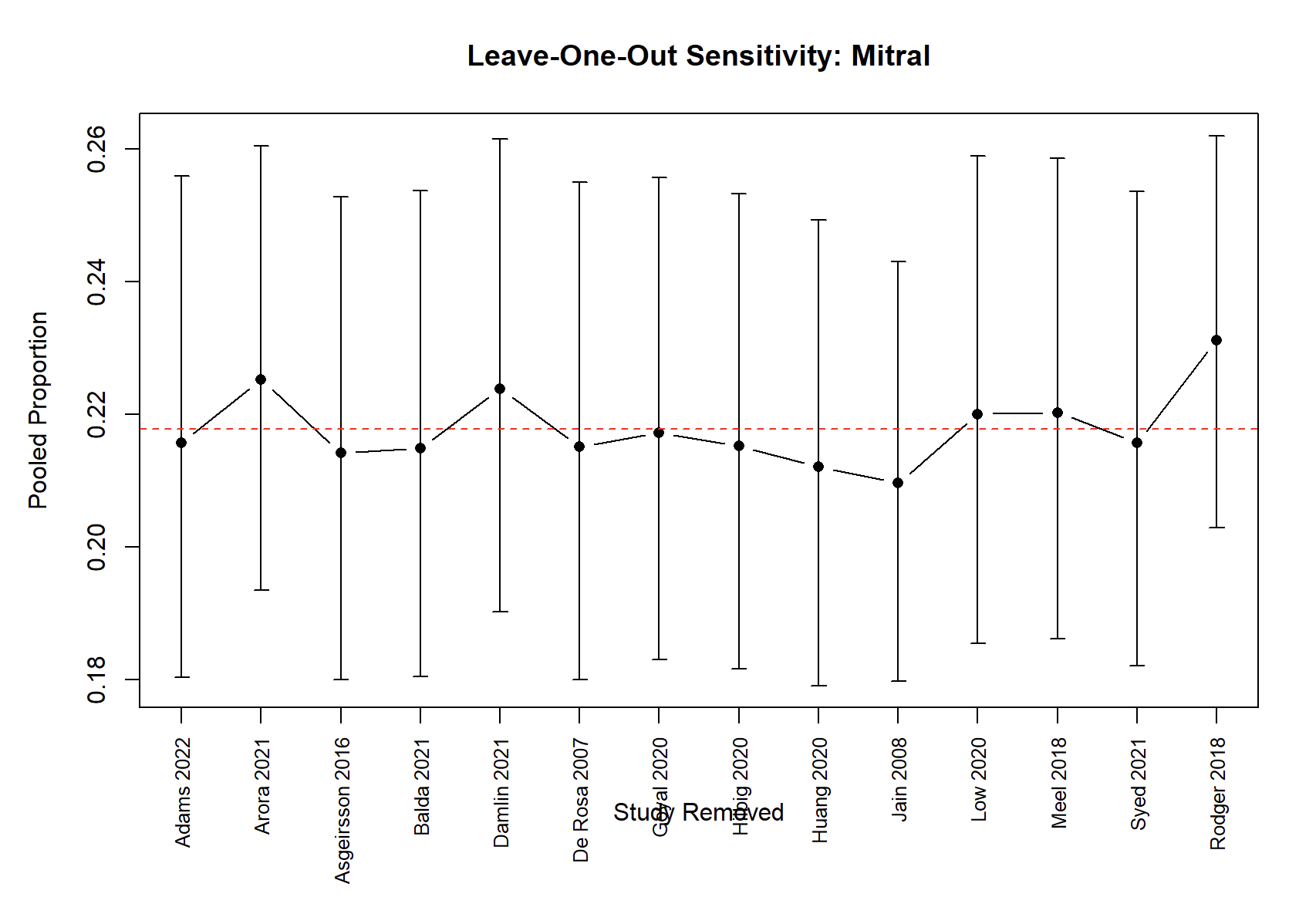
*

**Figure 45.** Leave-one-out analysis: Mitral valve

**Aortic**

The pooled prevalence of aortic valve involvement in IVDU-associated IE was 17% (95% CI: 14%–20%). Study-level estimates ranged from 3% to 29%, reflecting moderate variability. These findings confirm that while less frequent than tricuspid or mitral involvement, aortic valve IE is a clinically relevant concern and should be evaluated in all suspected cases.

*
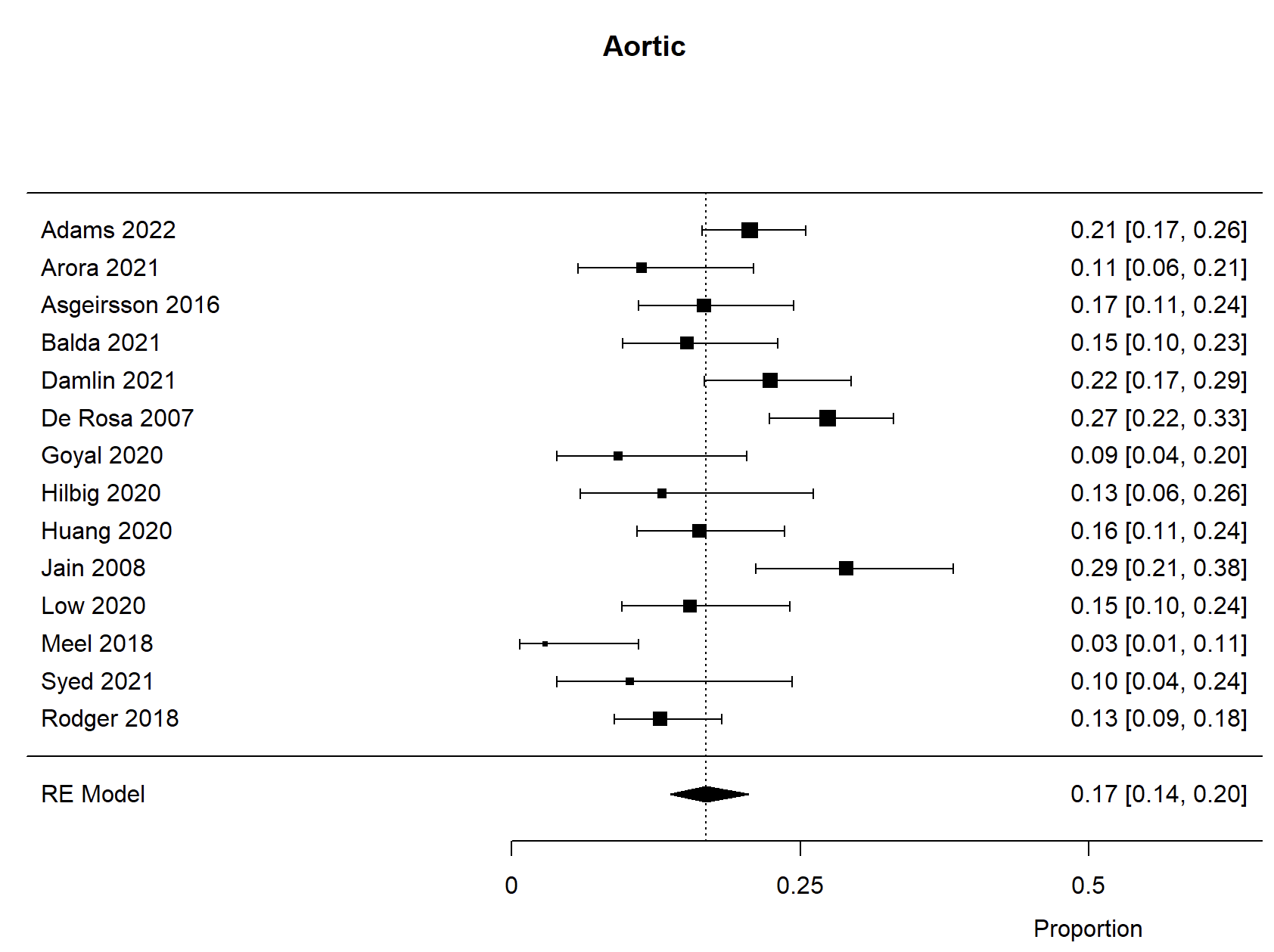
*

**Figure 46.** Forest plot: Aortic valve involvement after sensitivity adjustment

**Baujat plot: Aortic**

The Baujat plot identified Meel 2018 as the most influential contributor to heterogeneity in the aortic valve meta-analysis. De Rosa 2007 and Jain 2008 also showed moderate influence. These studies may warrant consideration for exclusion or subgroup analysis in sensitivity testing.

*
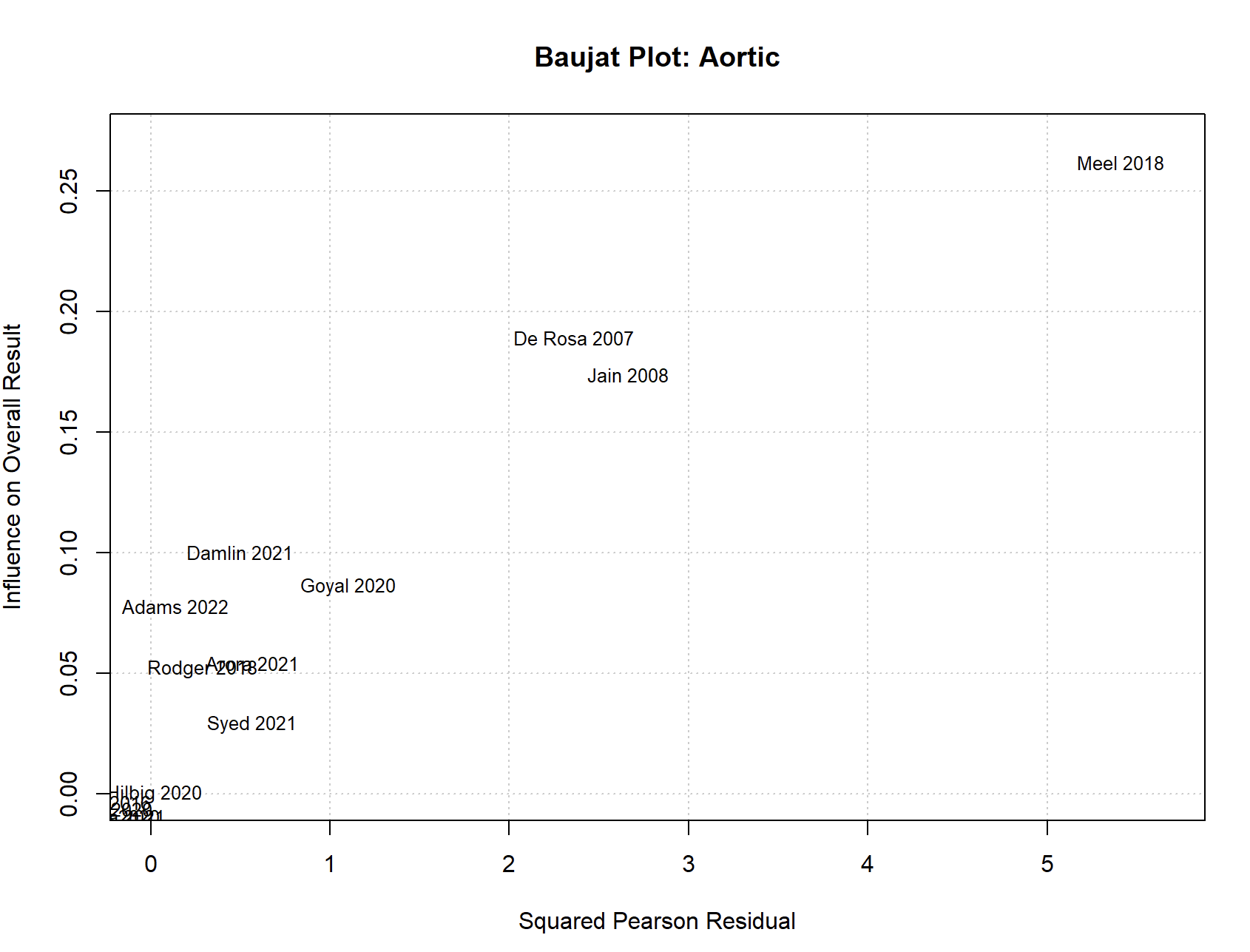
*

**Figure 47**. Baujat plot: Aortic valve heterogeneity

**Influence diagnostics: Aortic**

Influence diagnostics revealed that no single study exerted disproportionate influence across all metrics. However, moderate deviations were observed for Meel 2018, especially in *tau².del* and *QE.del*, suggesting it contributed to between-study heterogeneity. These results support including Meel 2018 in sensitivity analyses.

*
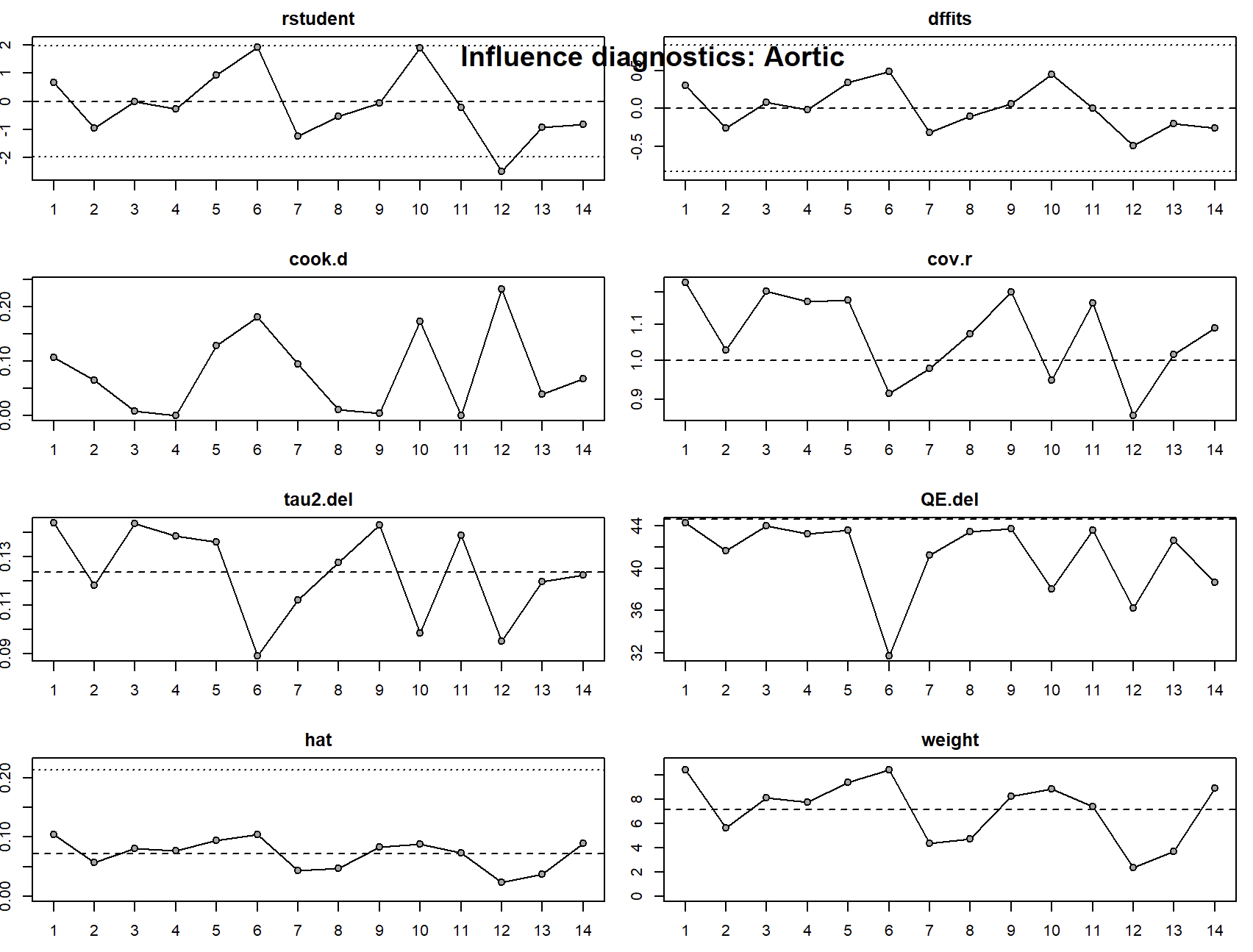
*

**Figure 48.** Influence diagnostics: Aortic valve

**Leave-One-Out Sensitivity: Aortic**

Leave-one-out sensitivity analysis for aortic valve involvement showed that no individual study significantly altered the pooled prevalence estimate. The pooled proportion remained stable, with overlapping confidence intervals throughout, supporting the robustness of the meta-analytic result.

*
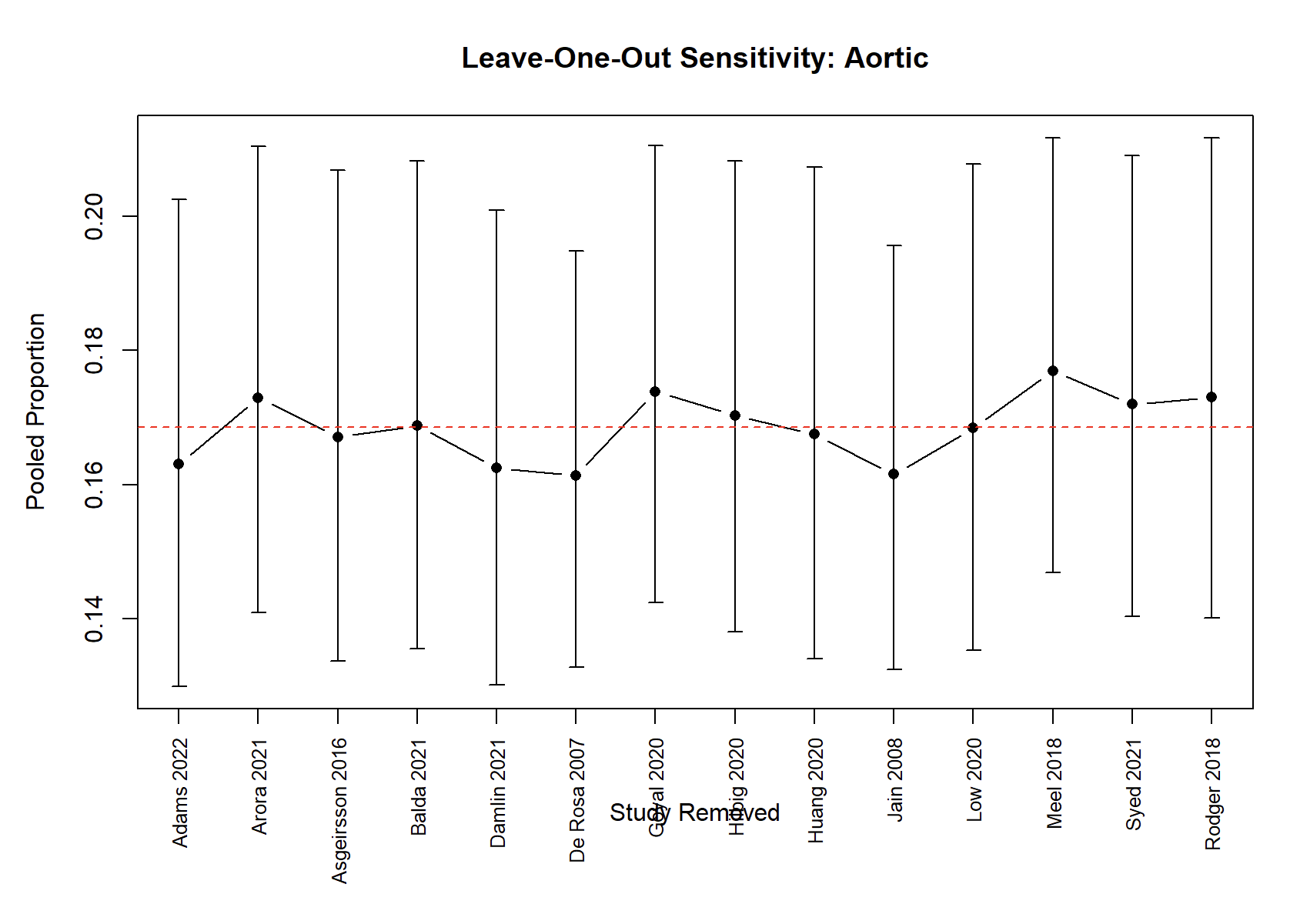
*

**Figure 49.** Leave-one-out analysis: Aortic valve

**Left**

The pooled prevalence of left-sided valve involvement in IVDU, associated IE was 39% (95% CI: 34%–45%), based on 8 studies. Individual study estimates ranged from 27% to 53%, reflecting moderate variability but consistent clinical significance. These findings emphasize that left heart involvement is not rare and warrants routine assessment in all IVDU-IE cases.

*
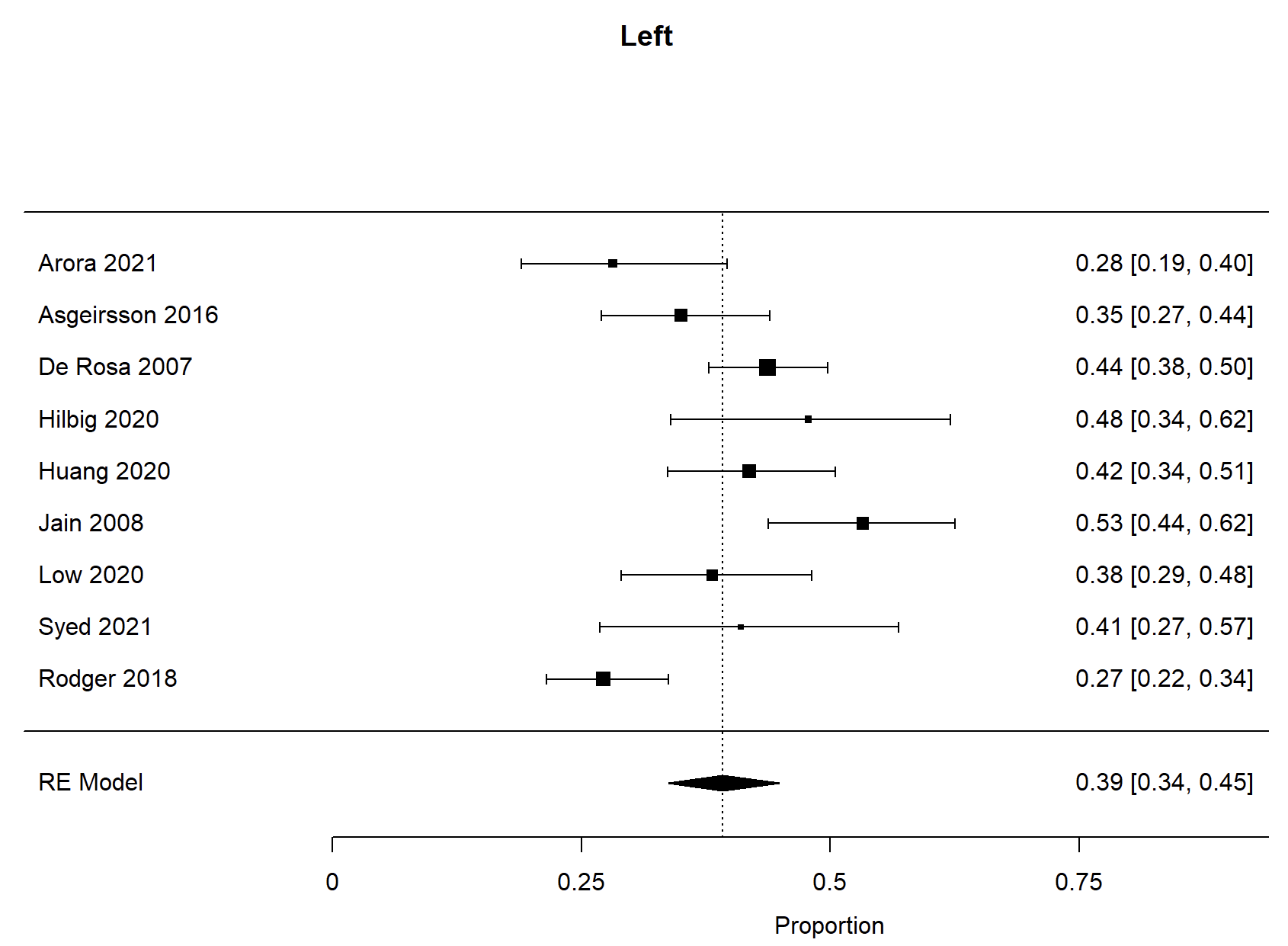
*

**Figure 50.** Forest plot: Left-sided IE after excluding outlier study

**Baujat plot: Left**

The Baujat plot shows Rodger 2018 and Jain 2008 contributed most to heterogeneity in left heart involvement. Arora 2021 had moderate influence; other studies showed minimal impact.

*
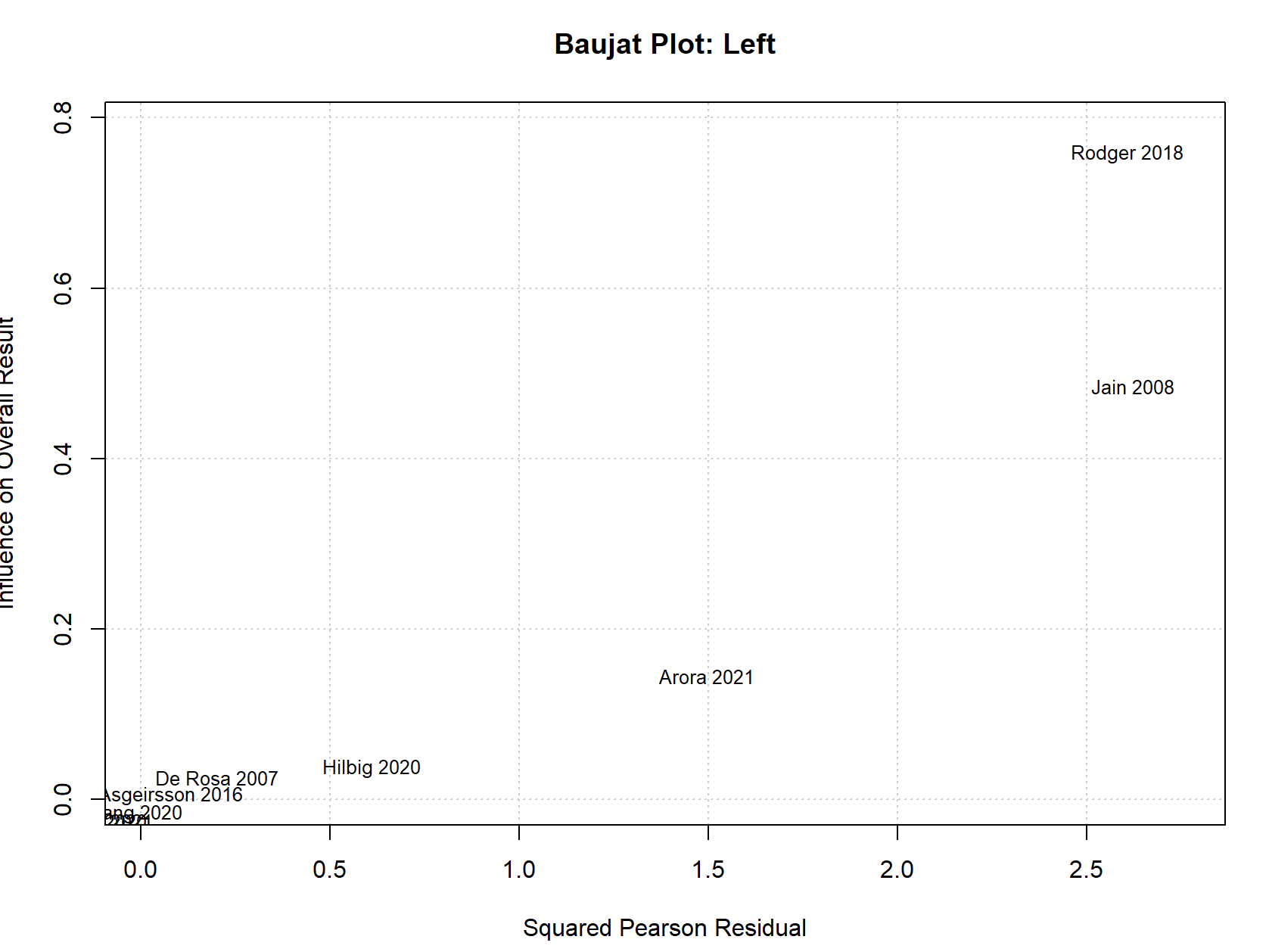
*

**Figure 51.** Baujat plot: Left-sided IE heterogeneity

**Influence diagnostics: Left**

Influence diagnostics indicated that study 9 (Rodger 2018) had the strongest impact on model estimates across multiple measures (e.g., cook.d, rstudent, QE.del), suggesting it as a potential outlier.

*
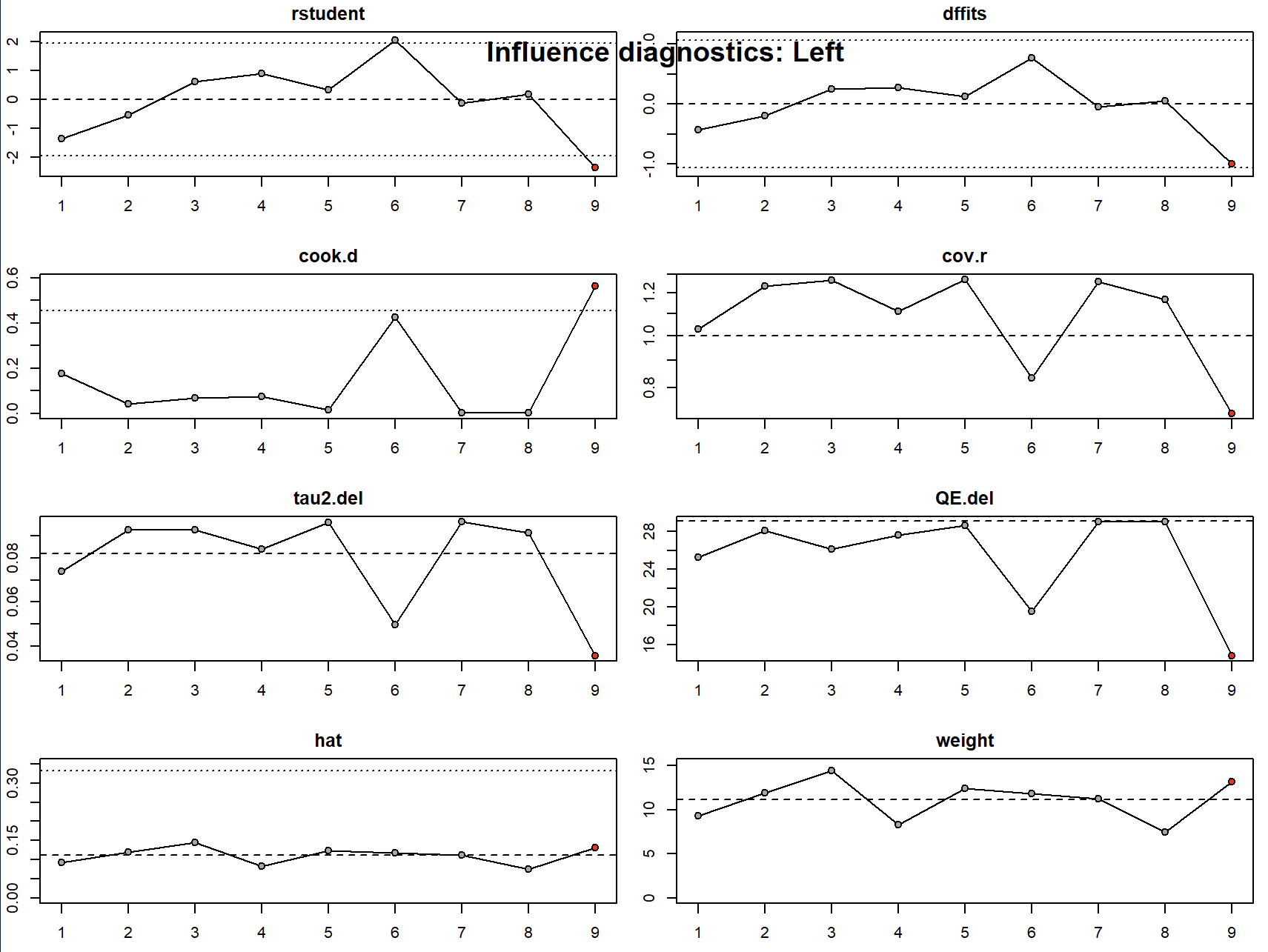
*

**Figure 52.** Influence diagnostics: Left-sided IE

**Leave-One-Out Sensitivity: Left**

Leave-one-out analysis showed that removing Rodger 2018 slightly increased the pooled estimate for left-sided involvement, while Jain 2008 reduced it. However, overall results remained stable, supporting the robustness of the pooled proportion.

*
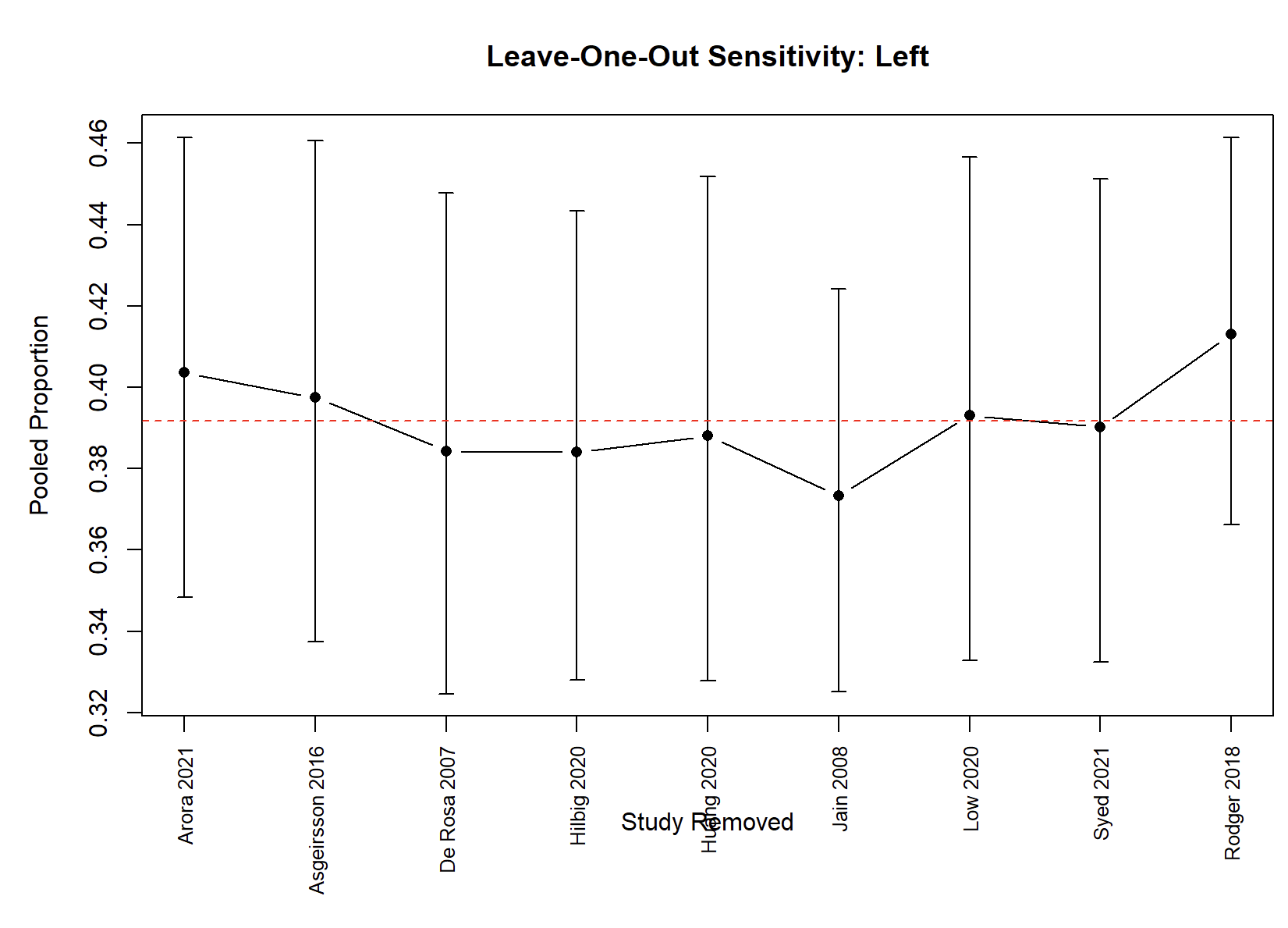
*

**Figure 53**. Leave-one-out analysis: Left-sided IE

**Right**

The pooled prevalence of right-sided valve involvement in IVDU, associated IE was 59% (95% CI: 55%–64%) across 9 studies. Most estimates ranged between 58% and 67%, with consistent confidence intervals. These results confirm the dominant involvement of the right heart, particularly the tricuspid valve in this population.

*
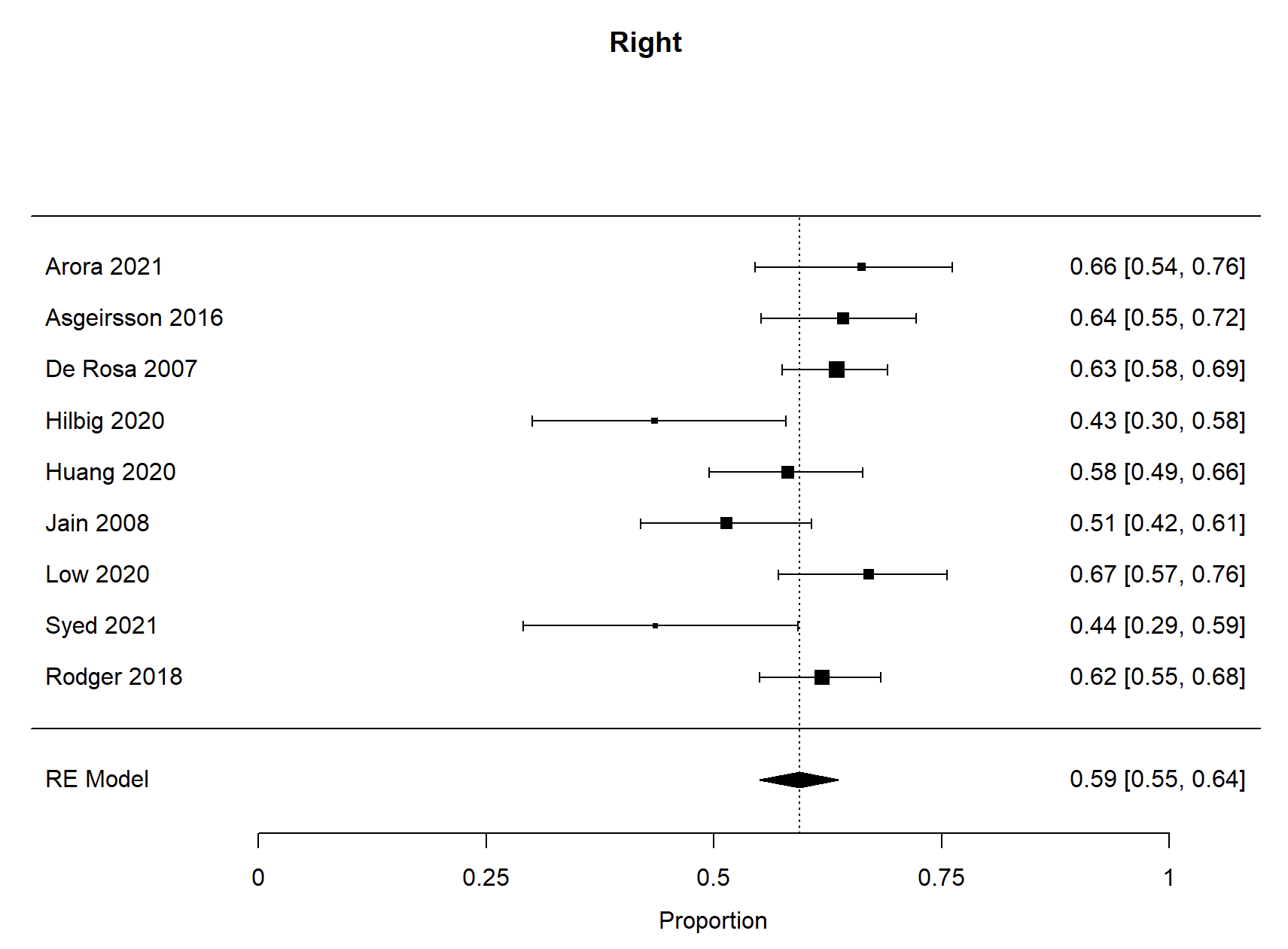
*

**Figure 54**. Forest plot: Right-sided IE after excluding influential study

**Baujat plot: Right**

The Baujat plot for right-sided involvement identified Hilbig 2020, Syed 2021, and Jain 2008 as the main contributors to heterogeneity. These studies may warrant further examination in sensitivity analyses.

*
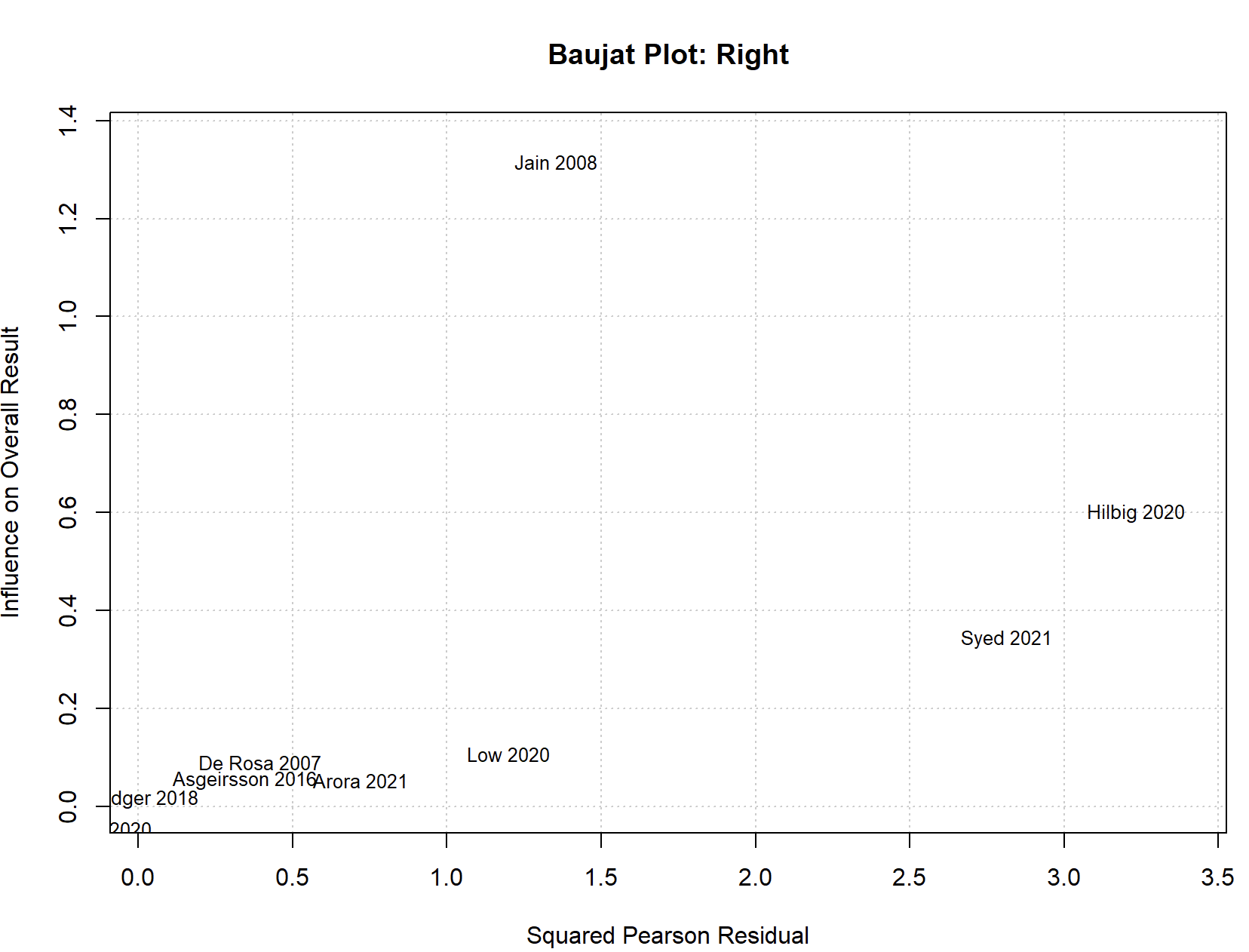
*

**Figure 55.** Baujat plot: Right-sided IE heterogeneity

**Influence diagnostic: Right**

Influence diagnostics identified study 6 as having the greatest influence across multiple metrics, including Cook’s distance, dffits, and QE.del. This suggests it may partially account for heterogeneity in right-sided valve estimates and should be explored in sensitivity analyses.

*
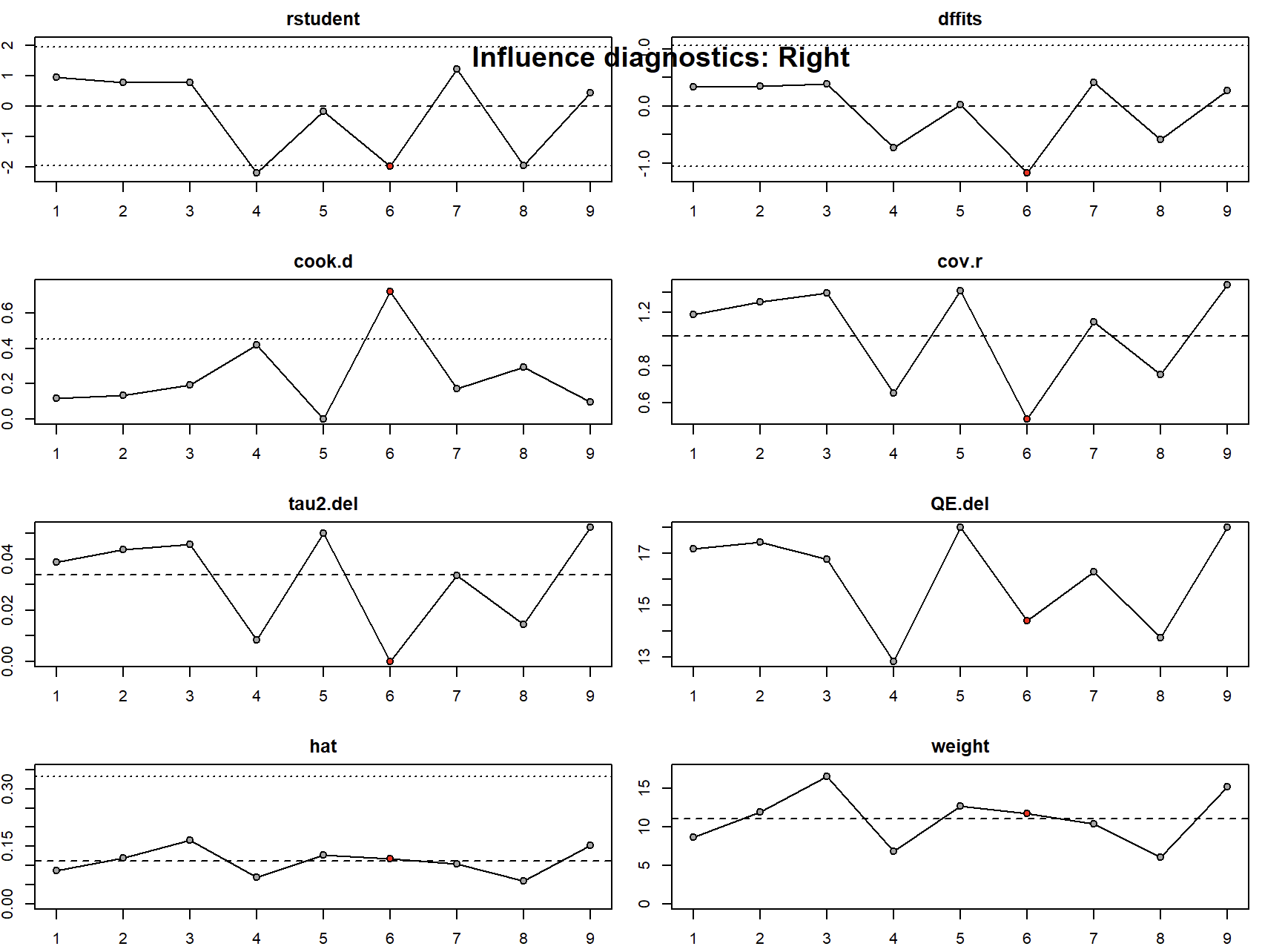
*

**Figure 56.** Influence diagnostics: Right-sided IE

**Leave-One-Out Sensitivity: Right**

Leave-one-out analysis for right-sided valve involvement showed that removal of individual studies, including Hilbig 2020 and Syed 2021, caused minor variation in the pooled proportion. However, all estimates remained within overlapping confidence intervals, confirming the robustness of the overall result.

*
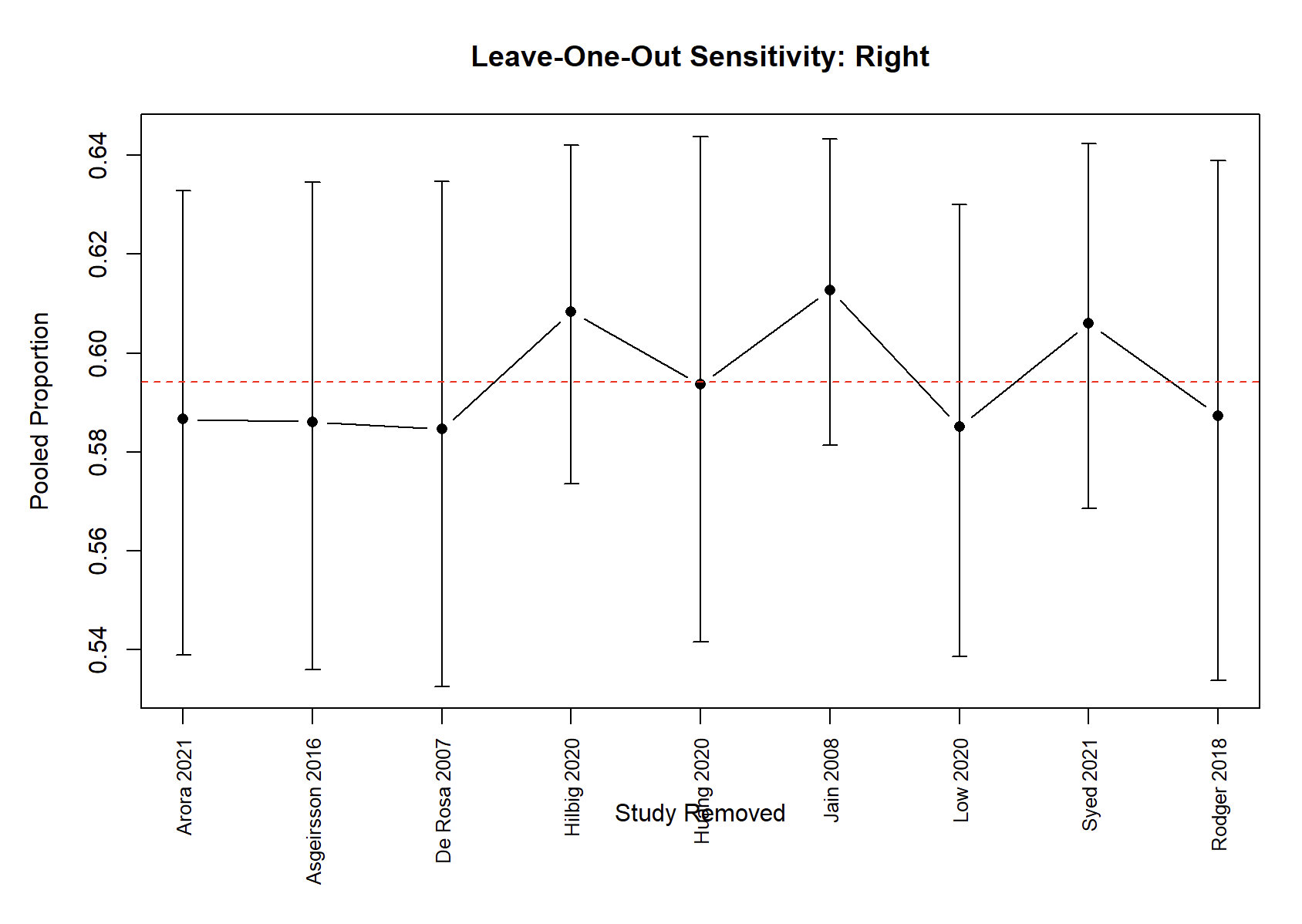
*

**Figure 57**. Leave-one-out analysis: Right-sided IE

**Both**

The pooled prevalence of both-sided valve involvement was 9% (95% CI: 6%–12%) across 9 studies. Although most estimates were low, a few reported higher rates (e.g., Asgeirsson 2016, Huang 2020). This confirms its rarity but clinical importance in select cases.

*
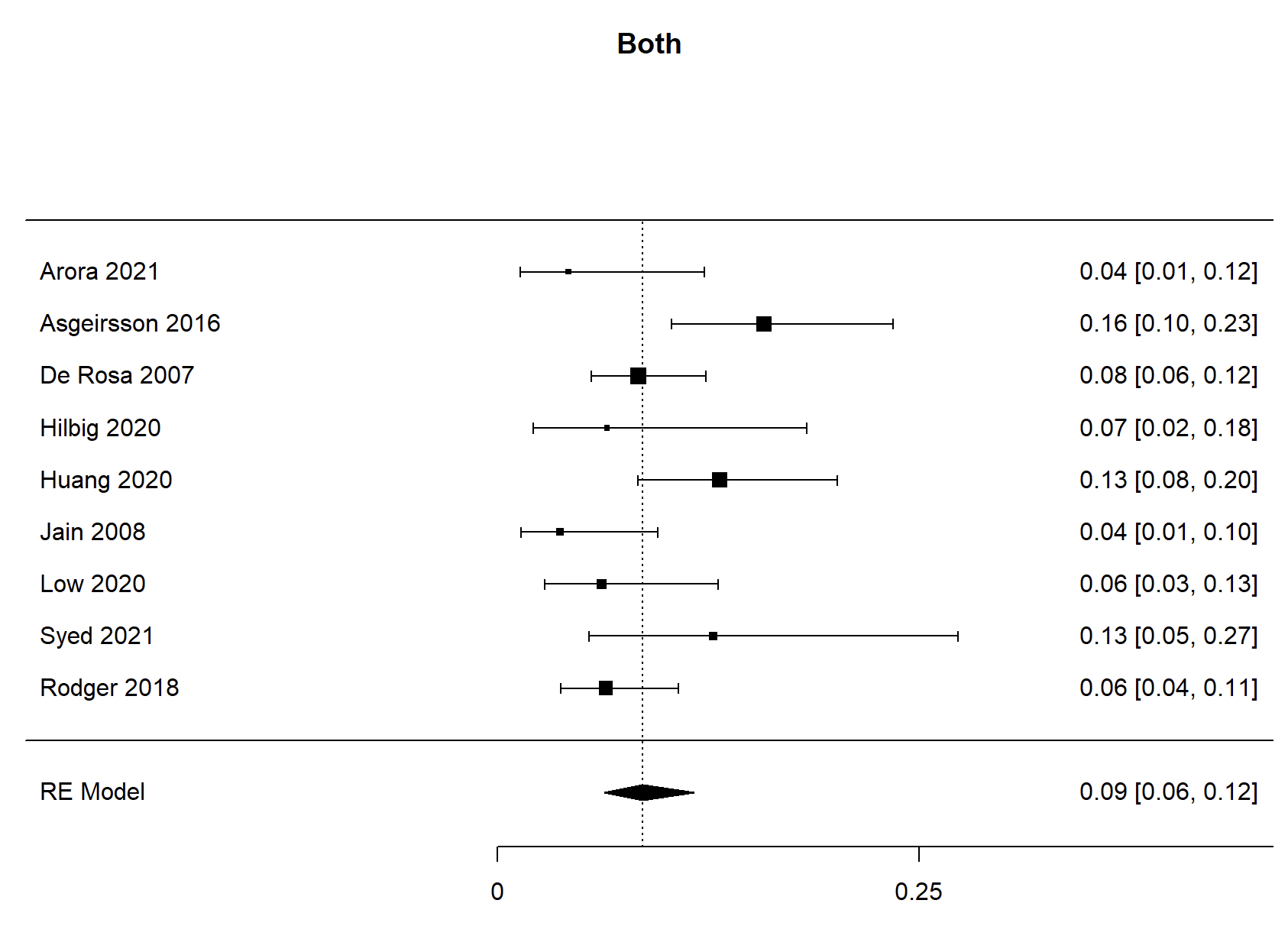
*

**Figure 58.** Forest plot: Both-sided IE after sensitivity adjustment

**Baujat plot: both**

The Baujat plot shows Asgeirsson 2016 and Jain 2008 as the most influential contributors to heterogeneity in both-sided valve involvement. Most other studies showed minimal influence.

*
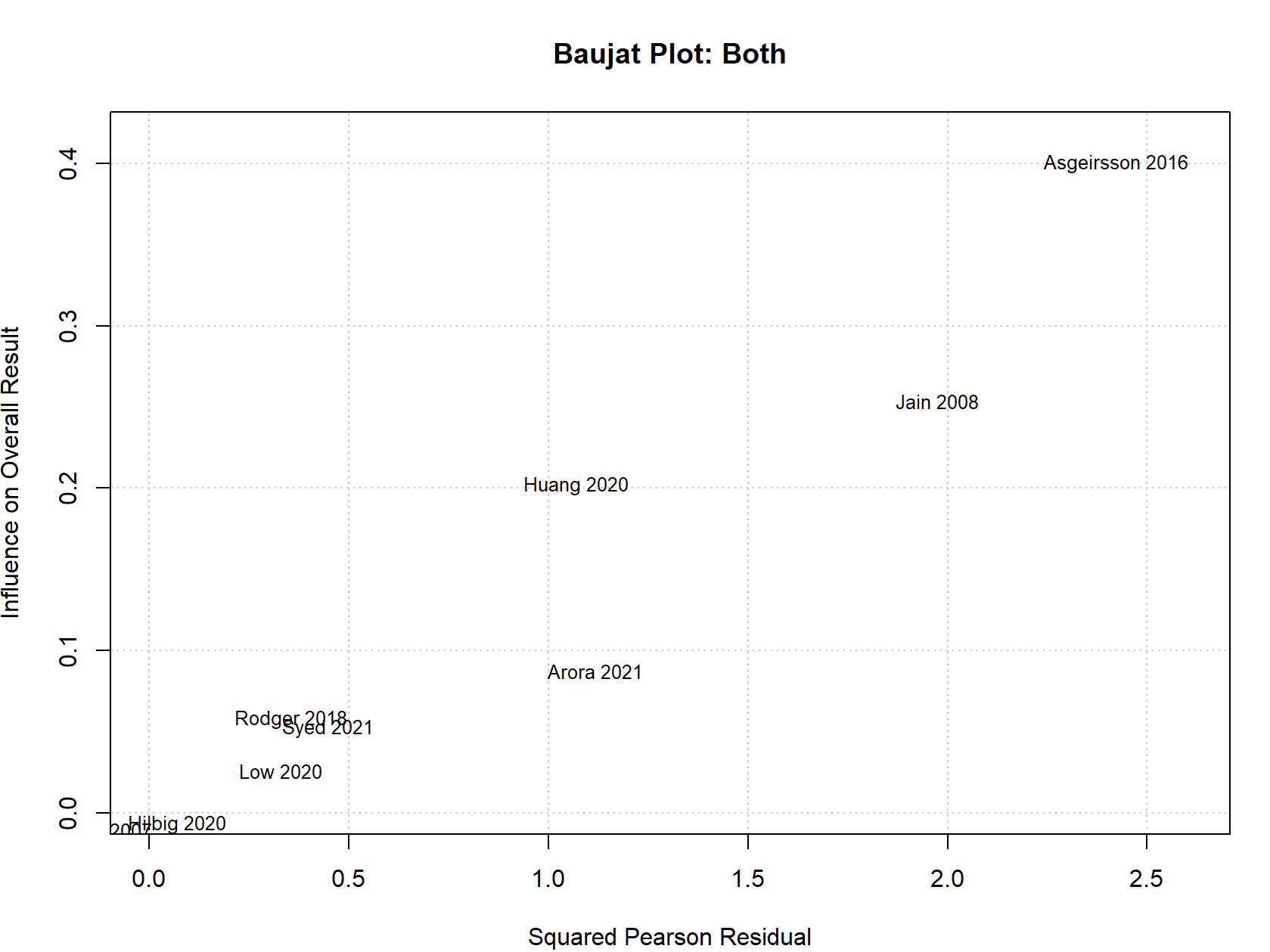
*

**Figure 59.** Baujat plot: Both-sided IE heterogeneity

**Influence diagnostics: Both**

Influence diagnostics for both-sided valve involvement identified study 3 as having elevated values across multiple metrics (e.g., rstudent, dffits, cook.d, tau2.del, QE.del), suggesting it may be an influential outlier. Other studies showed moderate to low influence, indicating overall model stability.

*
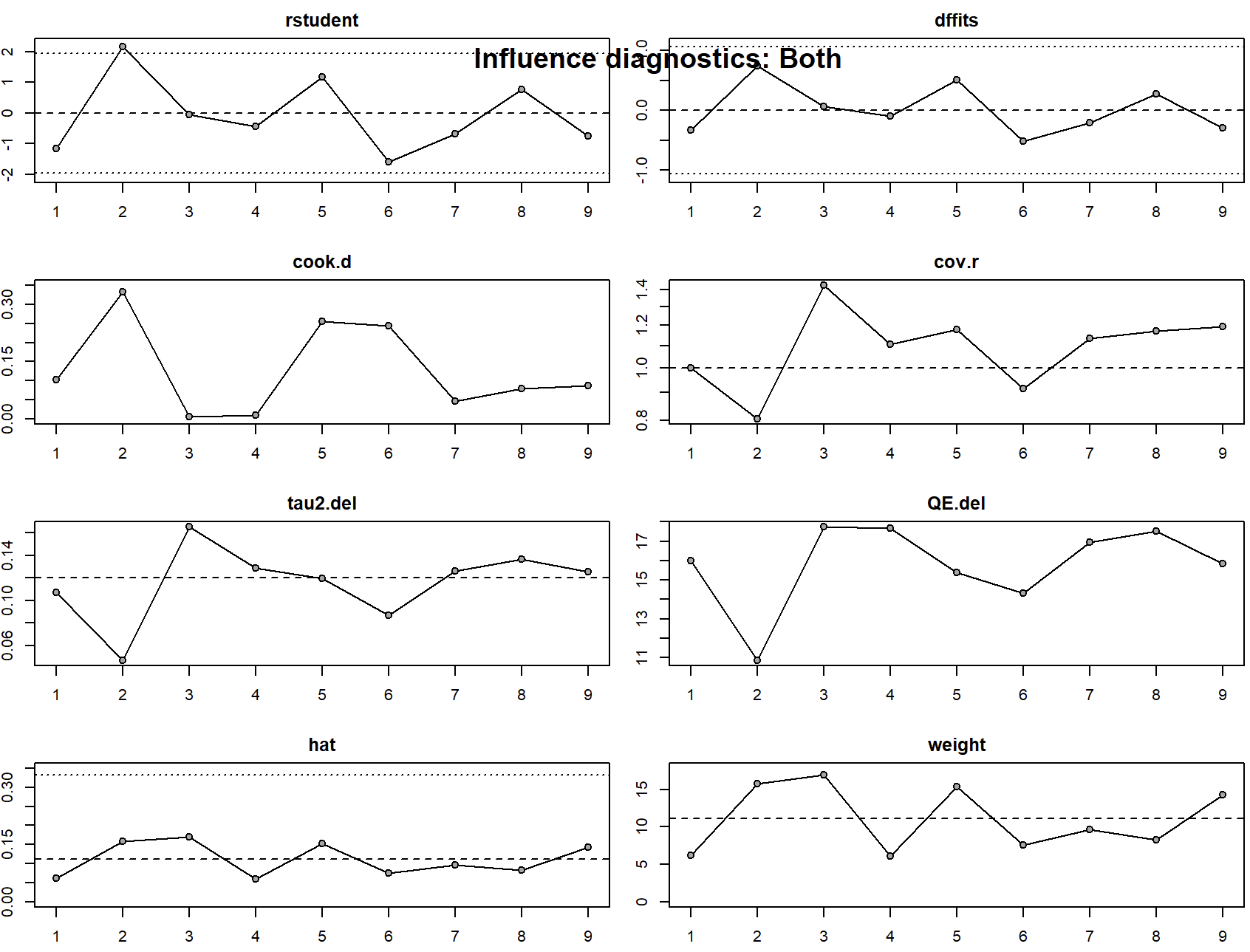
*

**Figure 60.** Influence diagnostics: Both-sided IE

**Leave-One-Out Sensitivity: Both**

Leave-one-out sensitivity analysis for both-sided valve involvement showed that no single study significantly altered the pooled estimate. The pooled proportions remained stable with overlapping confidence intervals, confirming the robustness of the overall result.

*
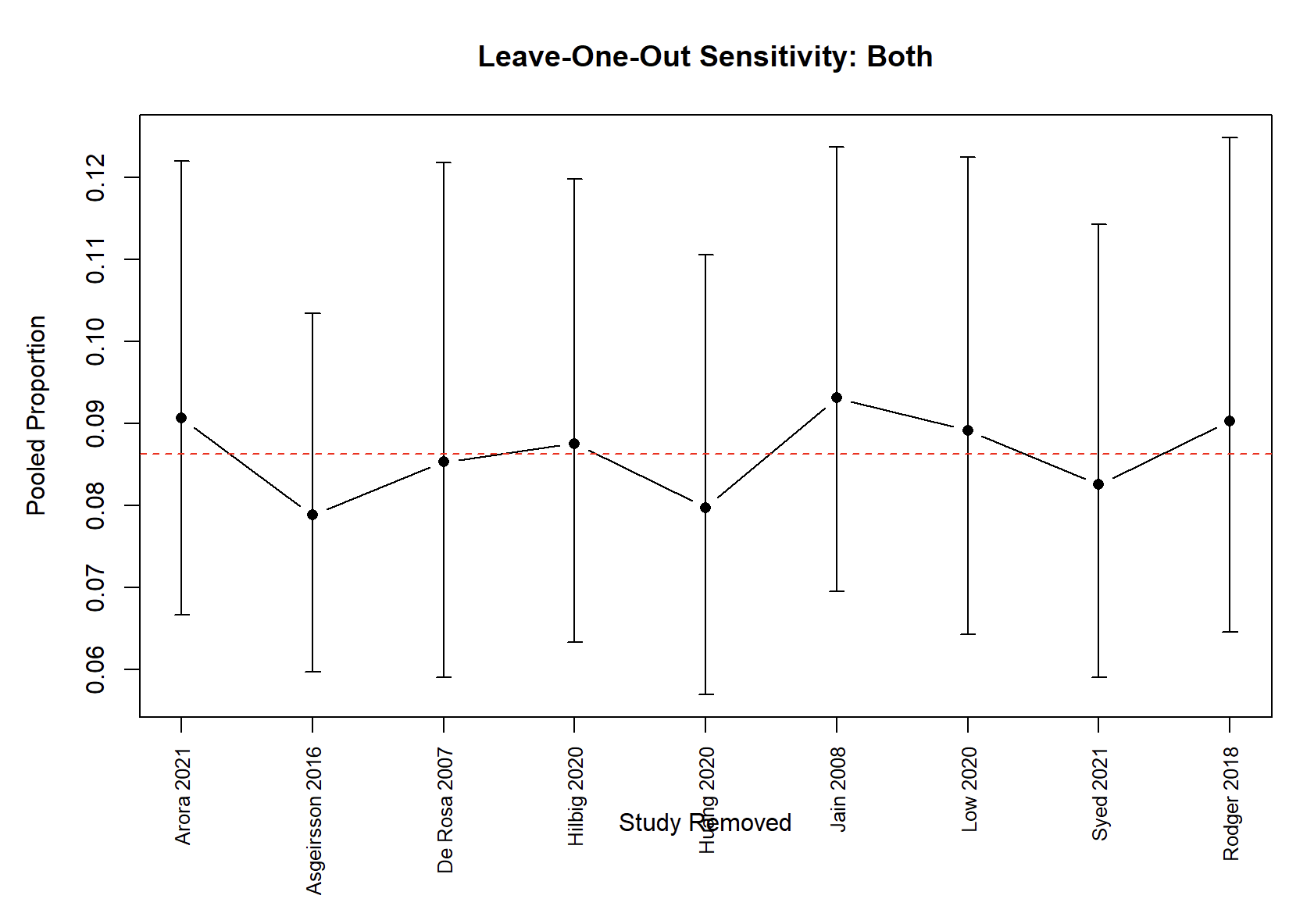
*

**Figure 61.** Leave-one-out analysis: Both-sided IE

# **Post-sensitivity analysis**

| **Variable (MA)** | **Effect size** | **Heterogeneity** | | | **Funnel**  **deviation** | **Egger’s**  **p-val** | **F-test**  **p-val** |
| --- | --- | --- | --- | --- | --- | --- | --- |
|  |  | **I²** | **tau²** | **H²** |  |  |  |
| Tricuspid valve (%)  *Excluded: Huang 2020* | 0.61 [0.57 – 0.64] | 47% | 0.0188 | 1.40 | Slight | 0.008 | 0.261 |
| Pulmonic valve (%)  *Excluded: None* | 0.01 [0.00 – 0.03] | 7% | 0.762 | 1.07 | Slight | 0.007 | 0.006 |
| Mitral valve (%)  *Excluded: Rodger 2018* | 0.23 [0.19 – 0.26] | 49% | 0.498 | 1.66 | Slight | 0.011 | 0.113 |
| Aortic valve (%)  *Excluded: Meel 2018* | 0.17 [0.14 – 0.21] | 67% | 0.101 | 1.49 | Skewed | 0.006 | 0.005 |
| Left heart (%)  *Excluded: Rodger 2018* | 0.41 [0.35 – 0.47] | 53% | 0.039 | 1.75 | None | 0.705 | 0.553 |
| Right heart (%)  *Excluded: Hilbig 2020* | 0.61 [0.57 – 0.65] | 45% | 0.008 | 1.00 | Slight | 0.021 | 0.227 |
| Both sides (%)  *Excluded: Asgeirsson 2016* | 0.07 [0.05 – 0.10] | 36% | 0.051 | 1.00 | None | 0.002 | 0.127 |
| Right heart risk (RR) | 1.49 [1.15 – 1.95] | 78% | 0.087 | 5.71 | None | 0.132 | 0.524 |
| S. aureus (%) | 0.73 [0.58 – 0.85] | 93% | 1.102 | 14.06 | Slight | 0.329 | 0.009 |
| MRSA (%) | 0.17 [0.06 – 0.38] | 95% | 1.943 | 12.99 | Slight | 0.500 | 0.009 |
| MSSA (%) | 0.63 [0.50 – 0.74] | 86% | 0.592 | 10.21 | Slight | 0.171 | 0.020 |
| Non-viridans S. (%) | 0.10 [0.05 – 0.19] | 90% | 0.755 | 6.24 | None | 0.630 | 0.090 |
| S. viridans (%) | 0.07 [0.02 – 0.28] | 97% | 1.568 | 8.32 | Slight | 0.656 | 0.070 |
| Valve surgeries (%) | 0.21 [0.15 – 0.28] | 79% | 0.267 | 5.37 | None | 0.095 | 0.928 |
| Mortality (all-cause) | 0.17 [0.12 – 0.25] | 89% | 0.491 | 5.61 | Slight | 0.363 | 0.010 |

**Tricuspid valve involvement**

Tricuspid valve involvement had a pooled prevalence of 61% (95% CI: 57%–64%) with moderate heterogeneity (I² = 47%). Most studies reported consistent proportions, reinforcing tricuspid as the most affected valve in IVDU-IE. The funnel plot was symmetric, indicating low publication bias.

*
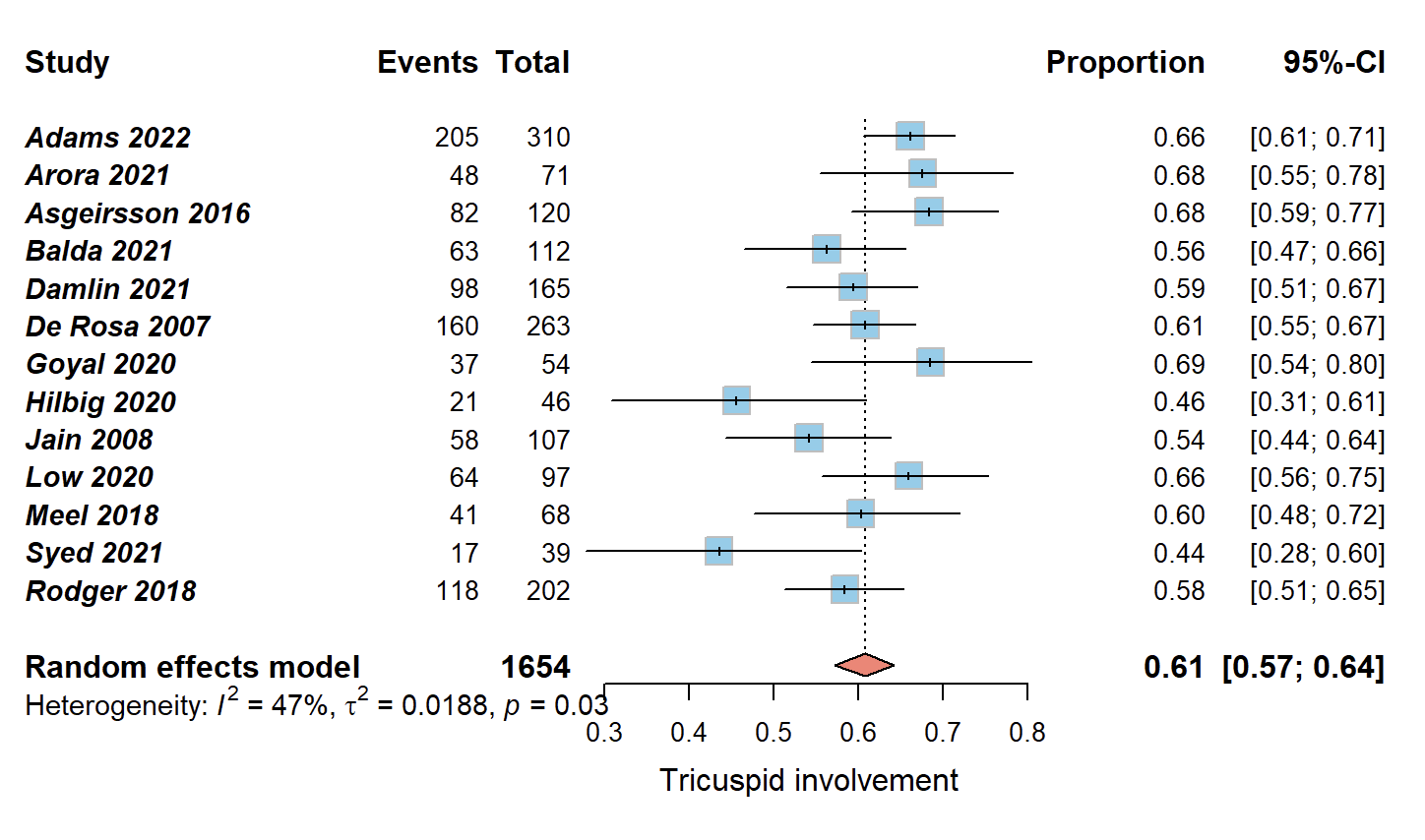
*

**Figure 62**. Forest plot: tricuspid valve involvement

*
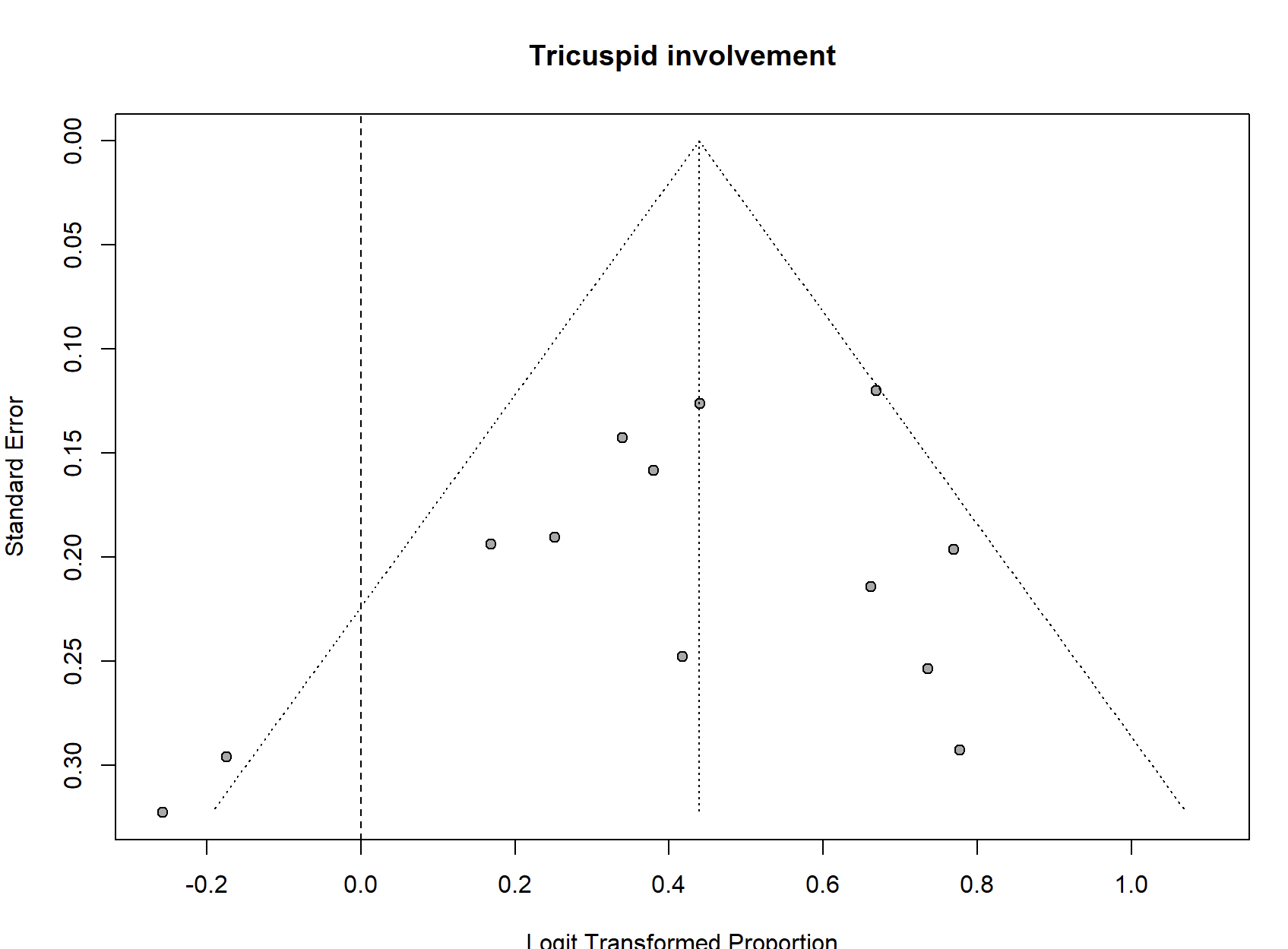
*

**Figure 63.** Funnel plot:tricuspid involvement

**Pulmonic valve involvement**

Pulmonic valve involvement was rare, with a pooled prevalence of 1% (95% CI: 0%–3%) and low heterogeneity (I² = 7%). Most studies reported no or very few cases. The funnel plot was largely symmetric, indicating minimal publication bias.

*
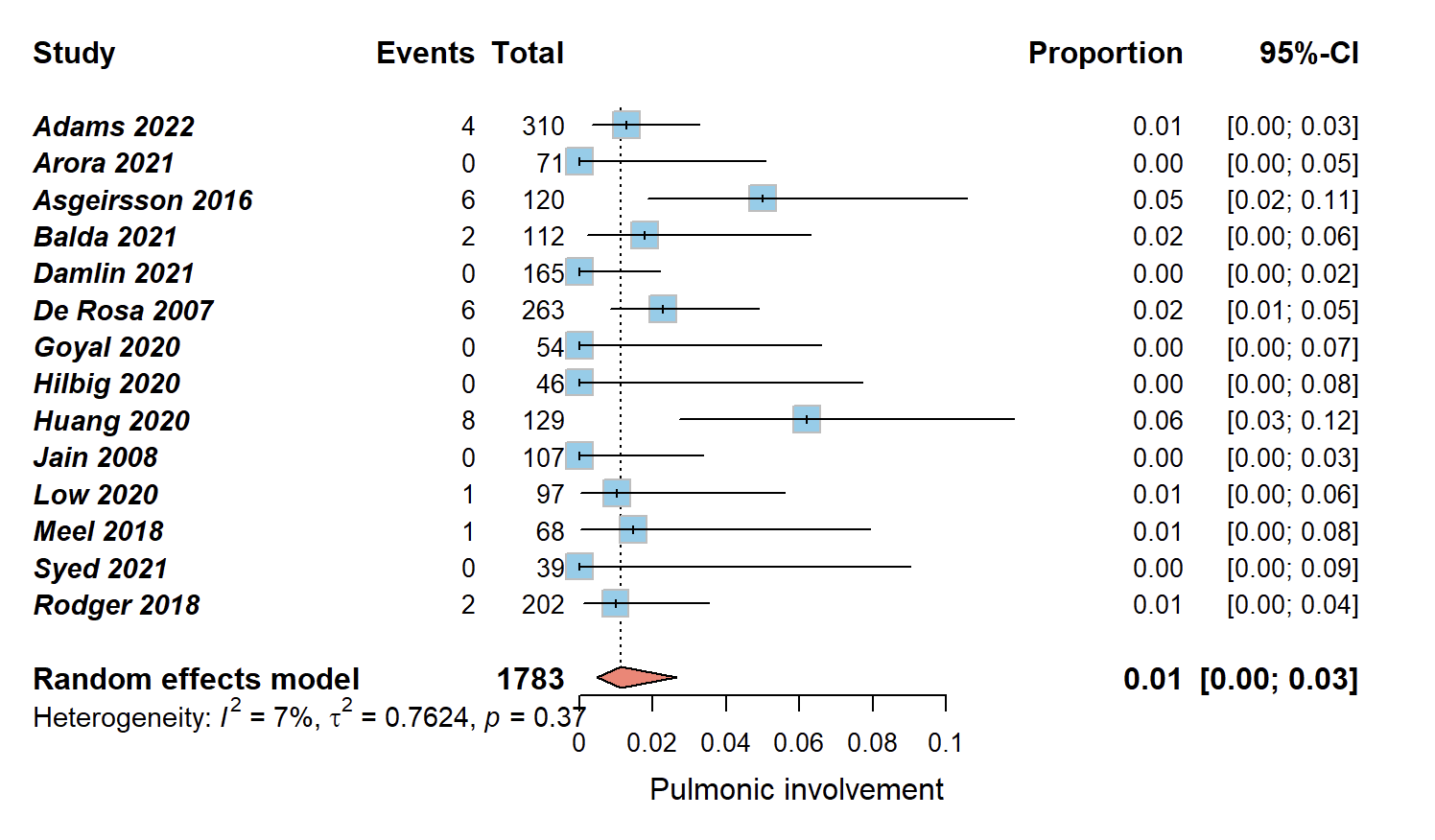
*

**Figure 64**. Forest plot: pulmonic valve involvement

*
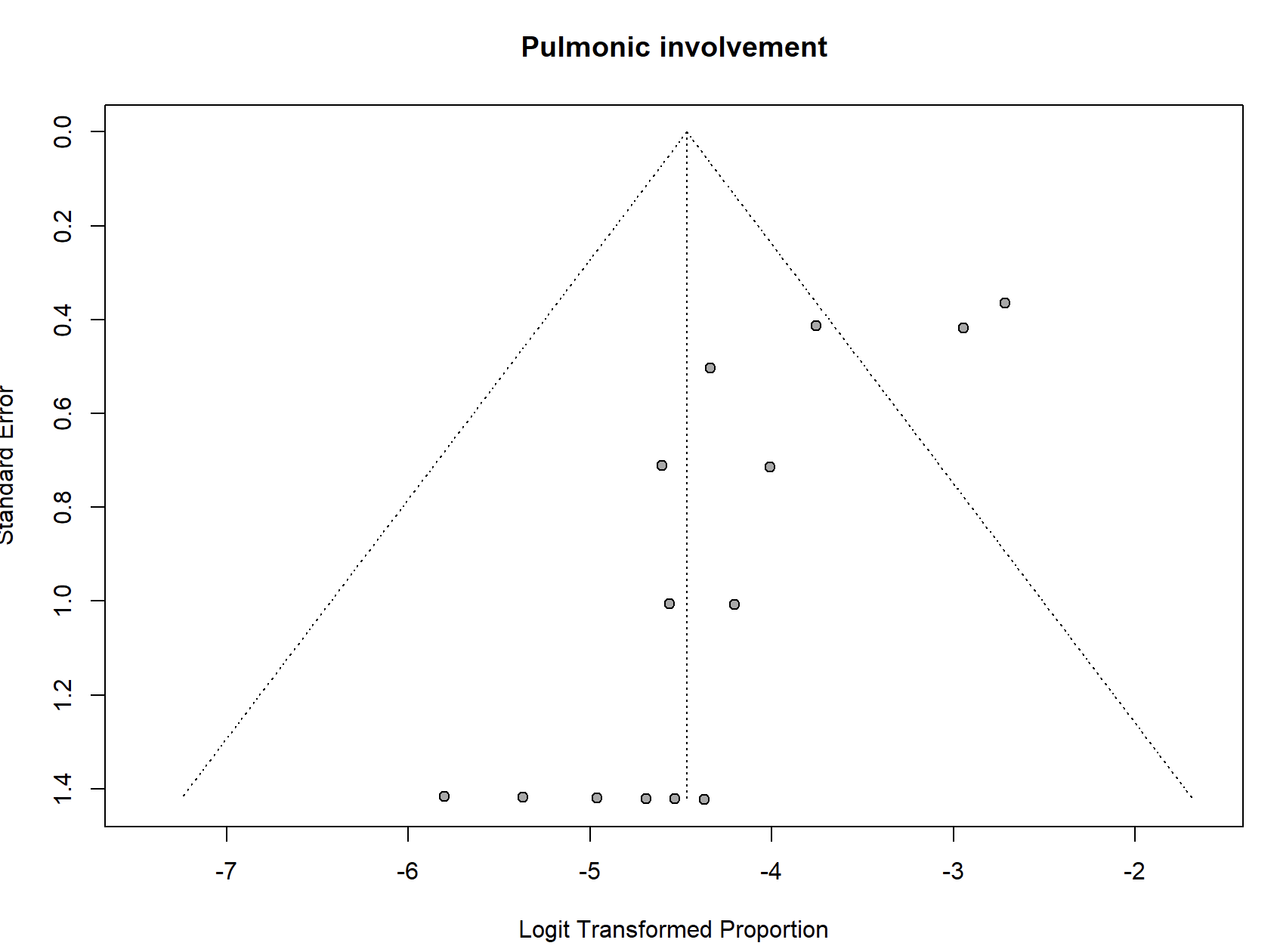
*

**Figure 65.** Funnel plot: Pulmonic involvement

**Mitral valve involvement**

Mitral valve involvement had a pooled prevalence of 23% (95% CI: 19%–26%) with moderate heterogeneity (I² = 49%). Study estimates ranged from 9% to 31%, showing some variability. The funnel plot was relatively symmetric, suggesting low risk of publication bias.

*
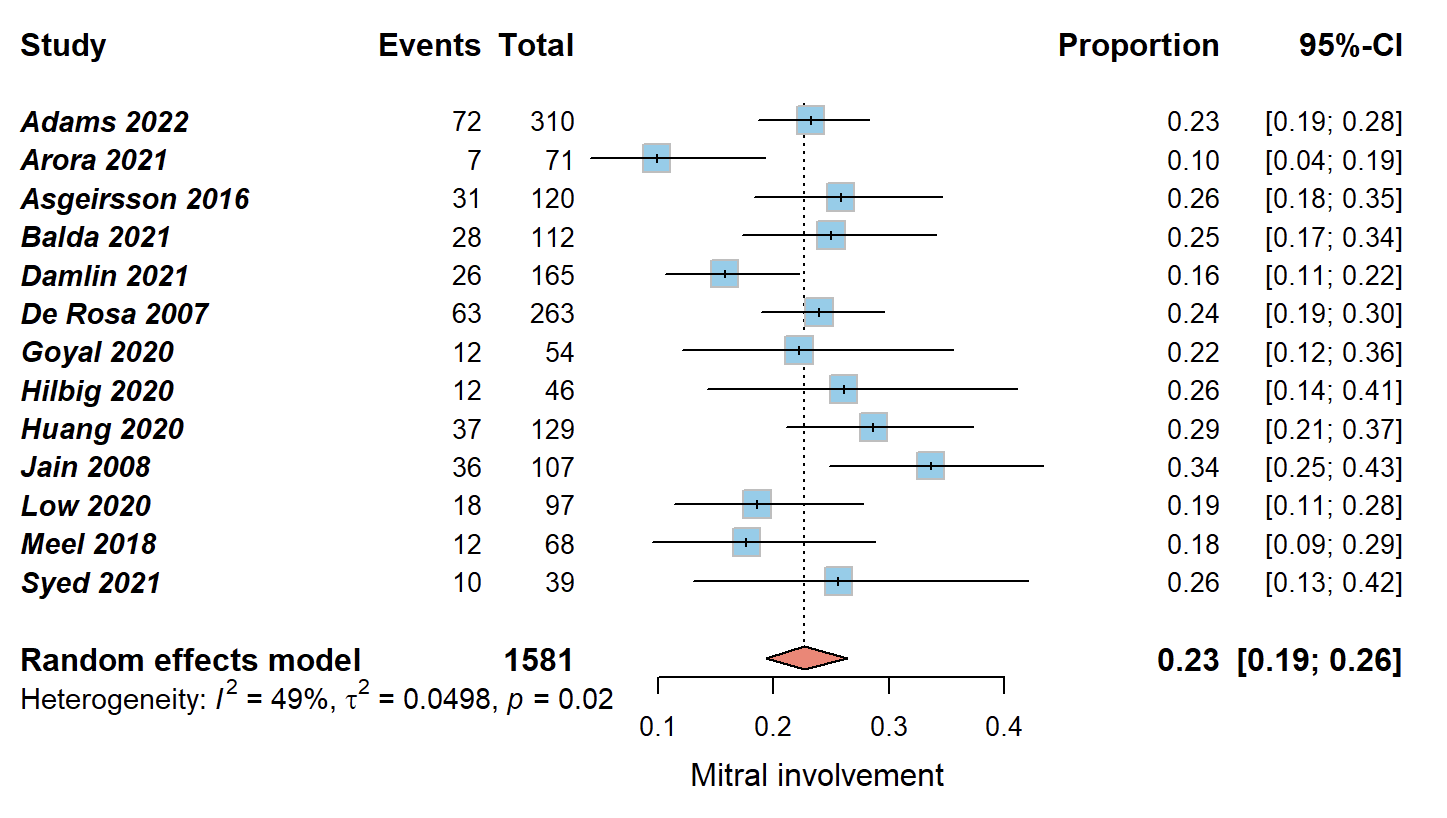
*

**Figure 66.** Forest plot: mitral valve involvement

*
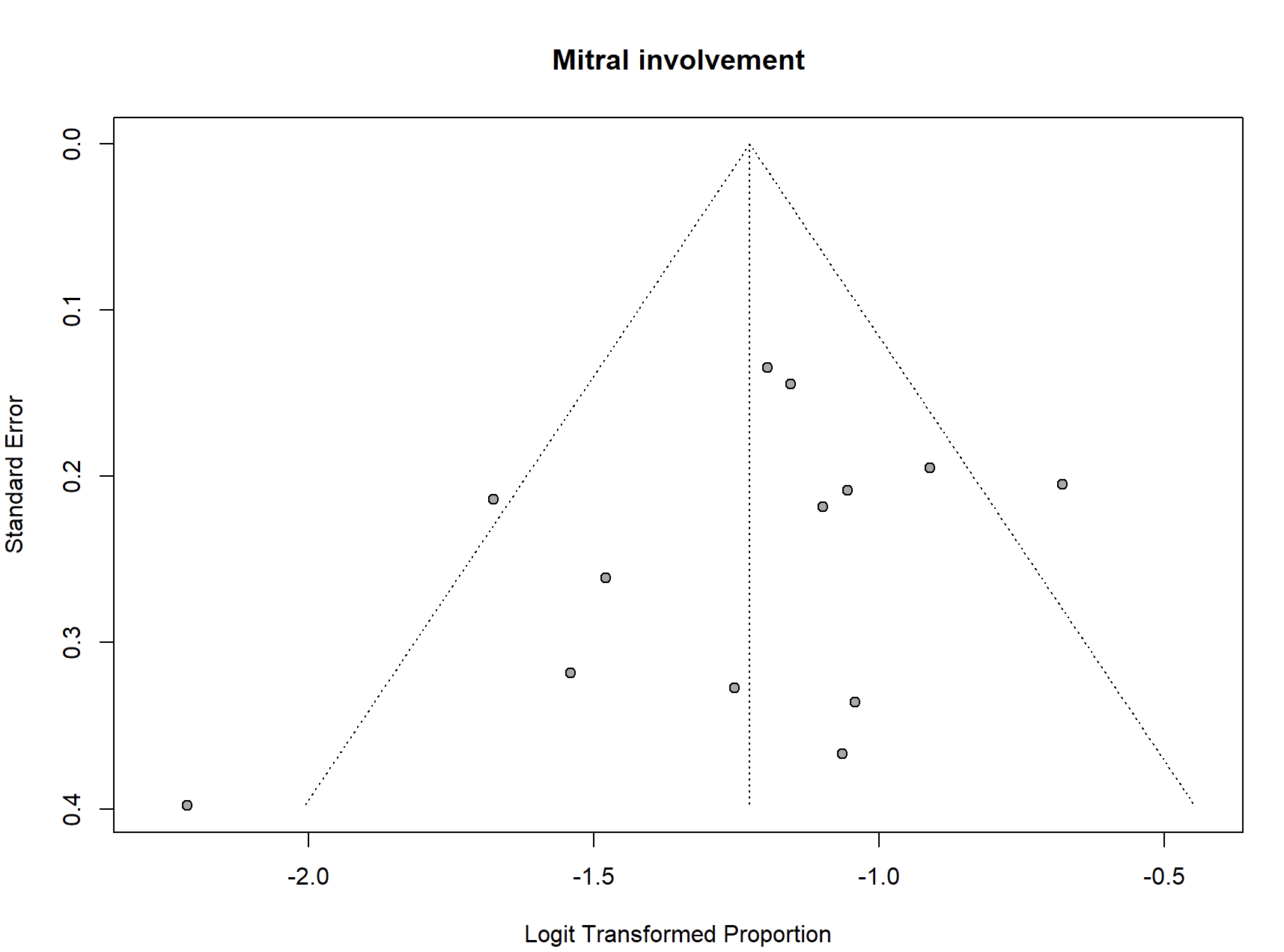
*

**Figure 67.** Funnel plot: mitral involvement

**Aortic valve involvement**

Aortic valve involvement had a pooled prevalence of 17% (95% CI: 14%–21%) with substantial heterogeneity (I² = 67%). Estimates varied across studies, ranging from 5% to 30%. The funnel plot showed slight asymmetry, suggesting possible small-study effects or heterogeneity.

*
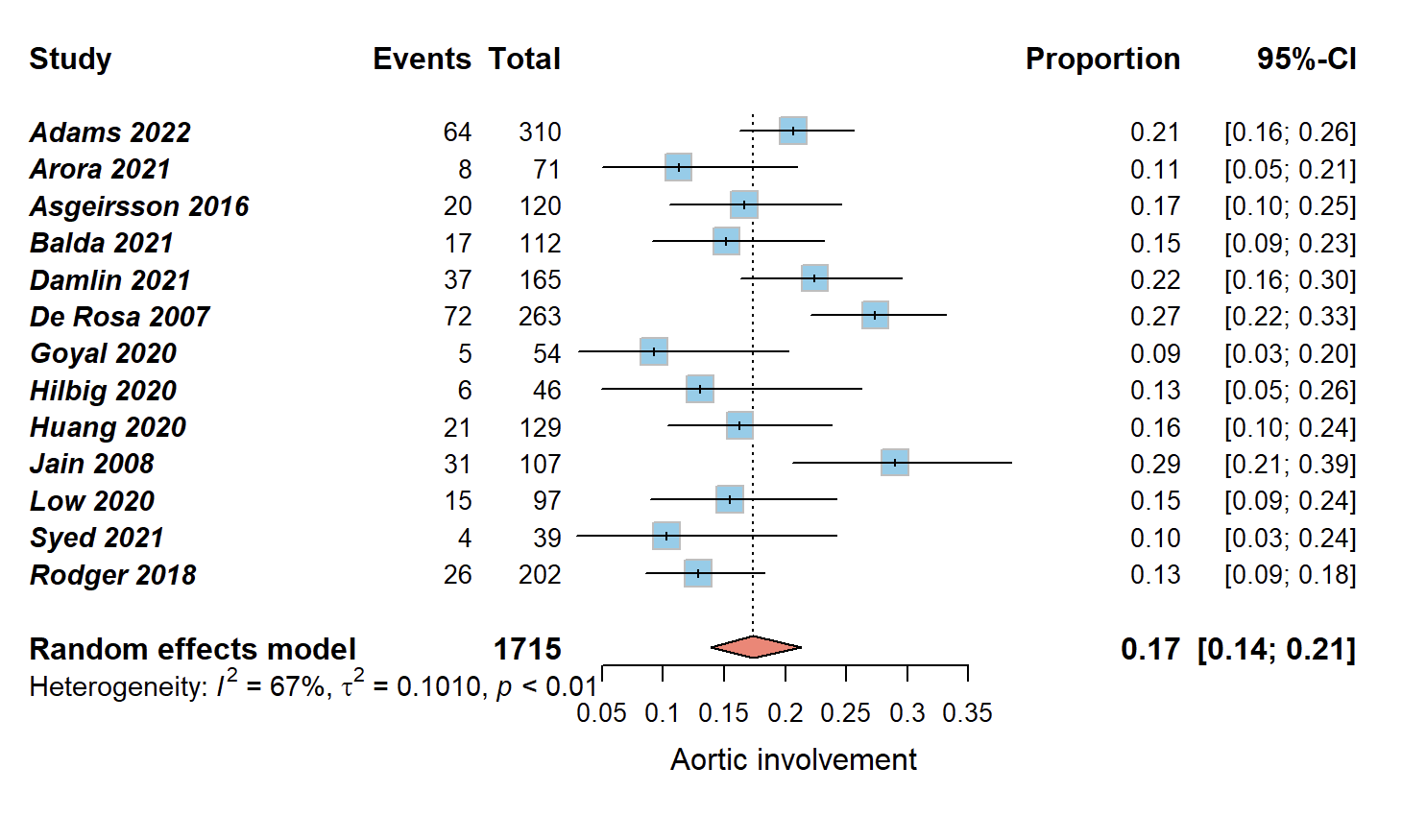
*

**Figure 68.** Forest plot: aortic valve involvement

*
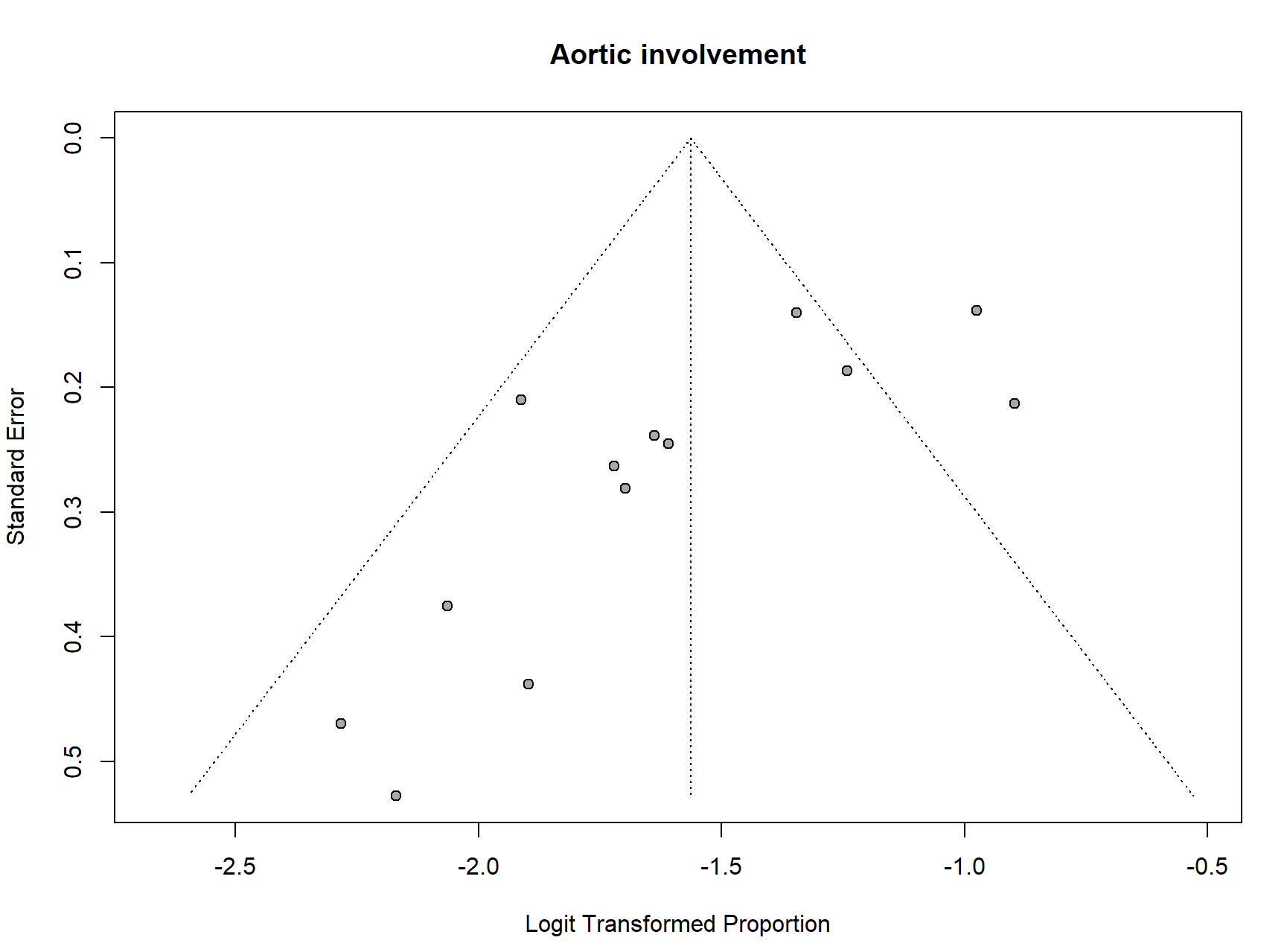
*

**Figure 69.** Funnel plot: aortic involvement

**Left-sided valve involvement**

Left-sided valve involvement showed a pooled prevalence of 41% (95% CI: 35%–47%) with moderate heterogeneity (I² = 53%). Study estimates ranged from 18% to 63%, indicating consistent findings. The funnel plot was symmetric, suggesting minimal publication bias.

*
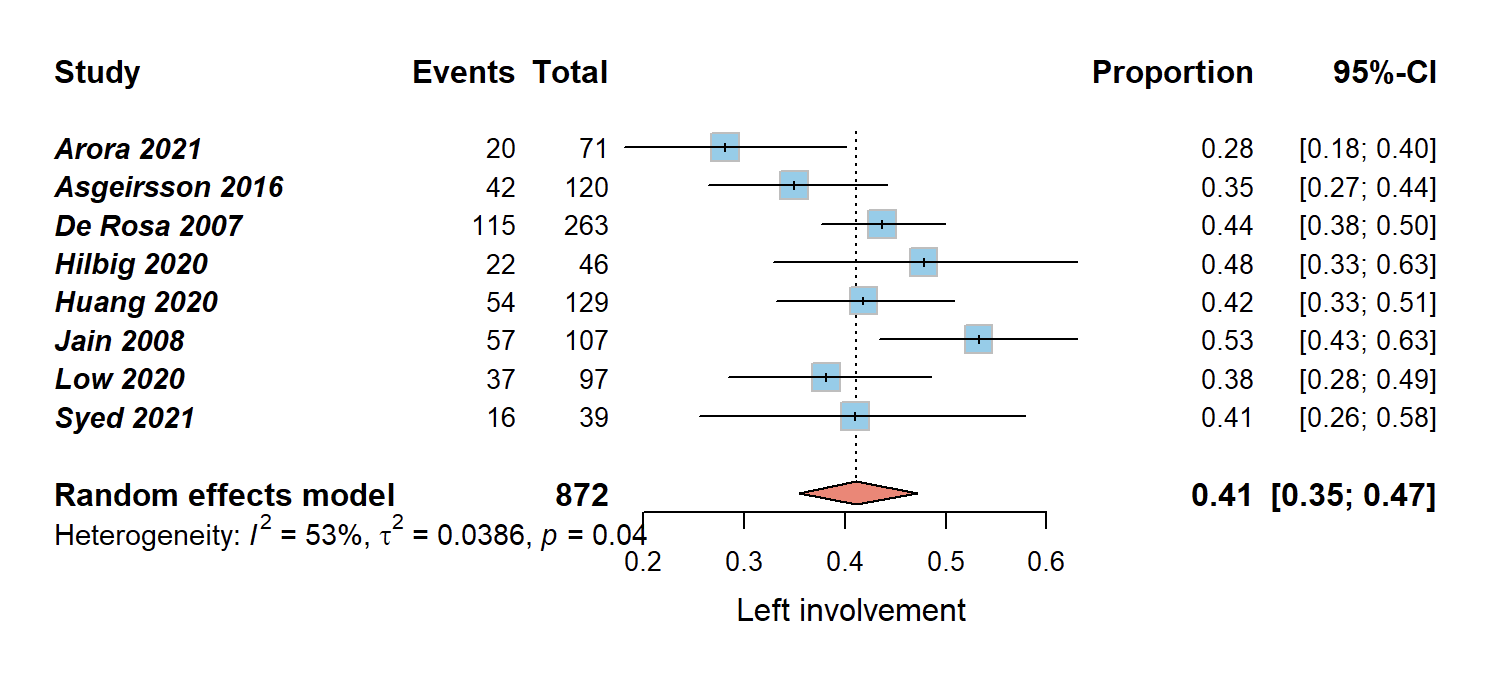
*

**Figure 70.** Forest plot: left-sided valve involvement

*
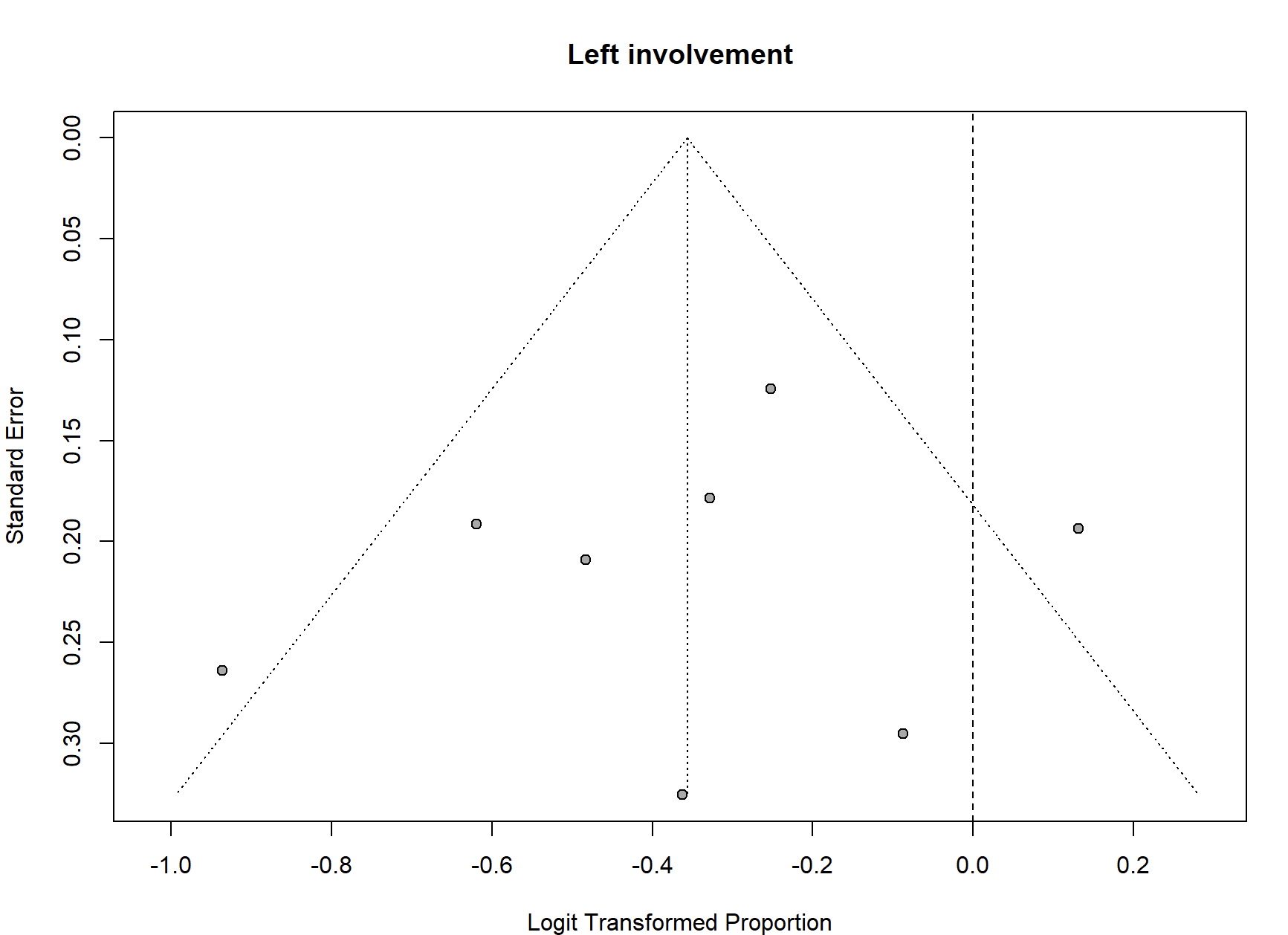
*

**Figure 71.** Funnel plot: left involvement

**Right sided valve involvement**

Right-sided valve involvement had a pooled prevalence of 61% (95% CI: 57%–65%) with moderate heterogeneity (I² = 45%). Most studies reported proportions above 50%, confirming its predominance in IVDU-associated IE. The funnel plot appeared symmetric, indicating low publication bias.

*
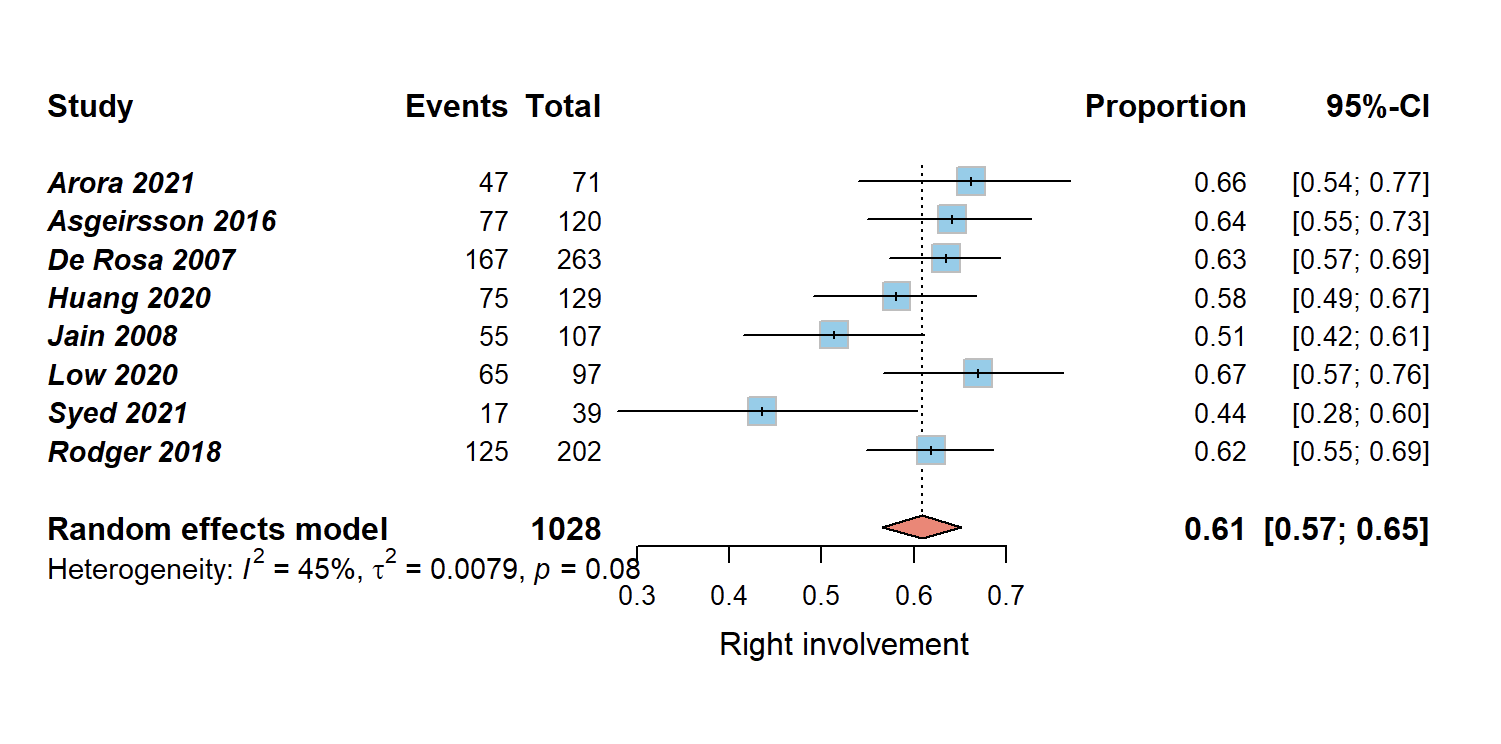
*

**Figure 72.** Forest plot: right valve involvement

*
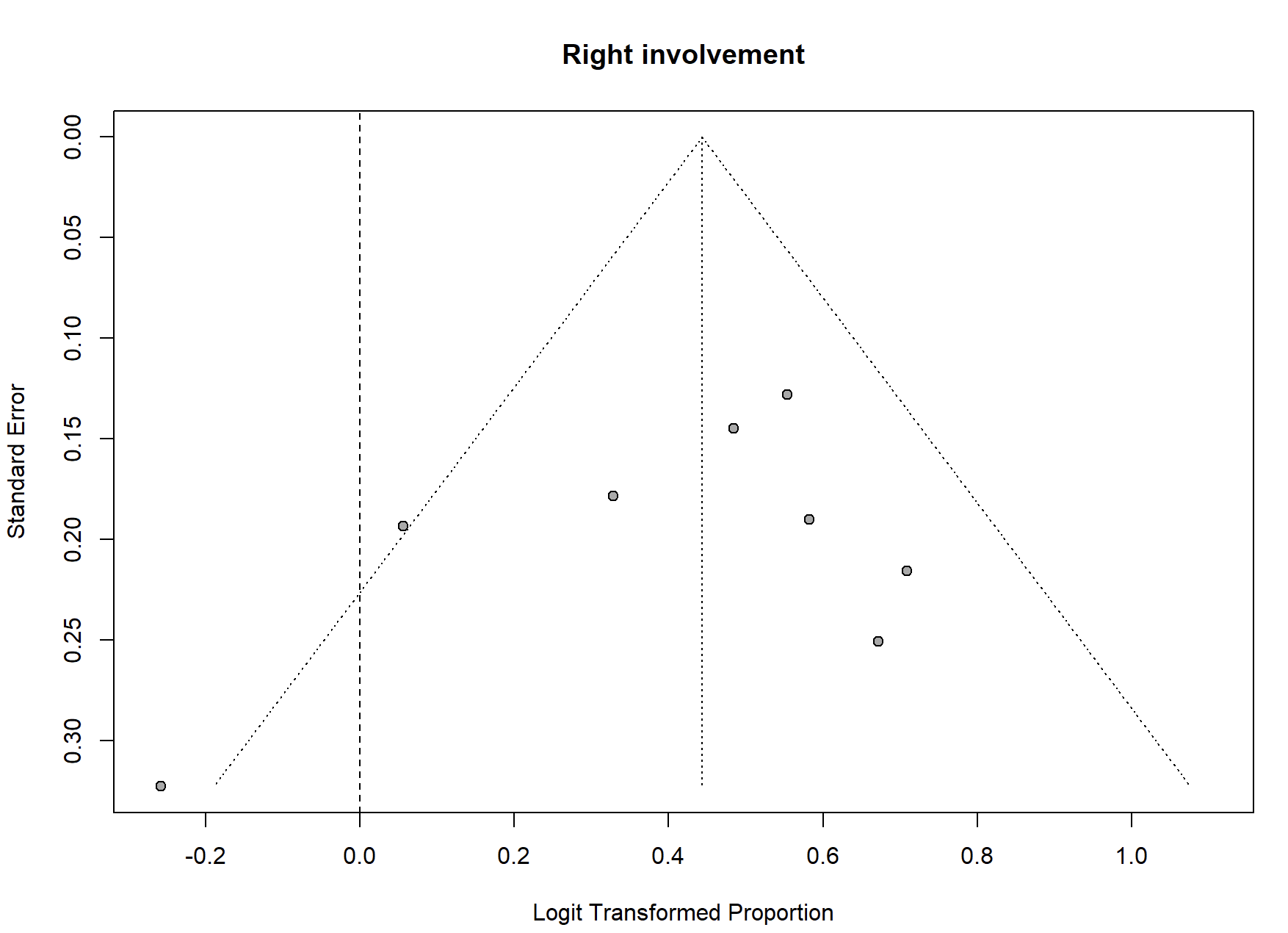
*

**Figure 73.** Funnel plot: right valve involvement

**Both sided valve involvement**

Both-sided valve involvement had a pooled prevalence of 7% (95% CI: 5%–10%) with low-to-moderate heterogeneity (I² = 36%). Study estimates were consistently low, confirming its rarity. The funnel plot was symmetric, indicating minimal publication bias.

*
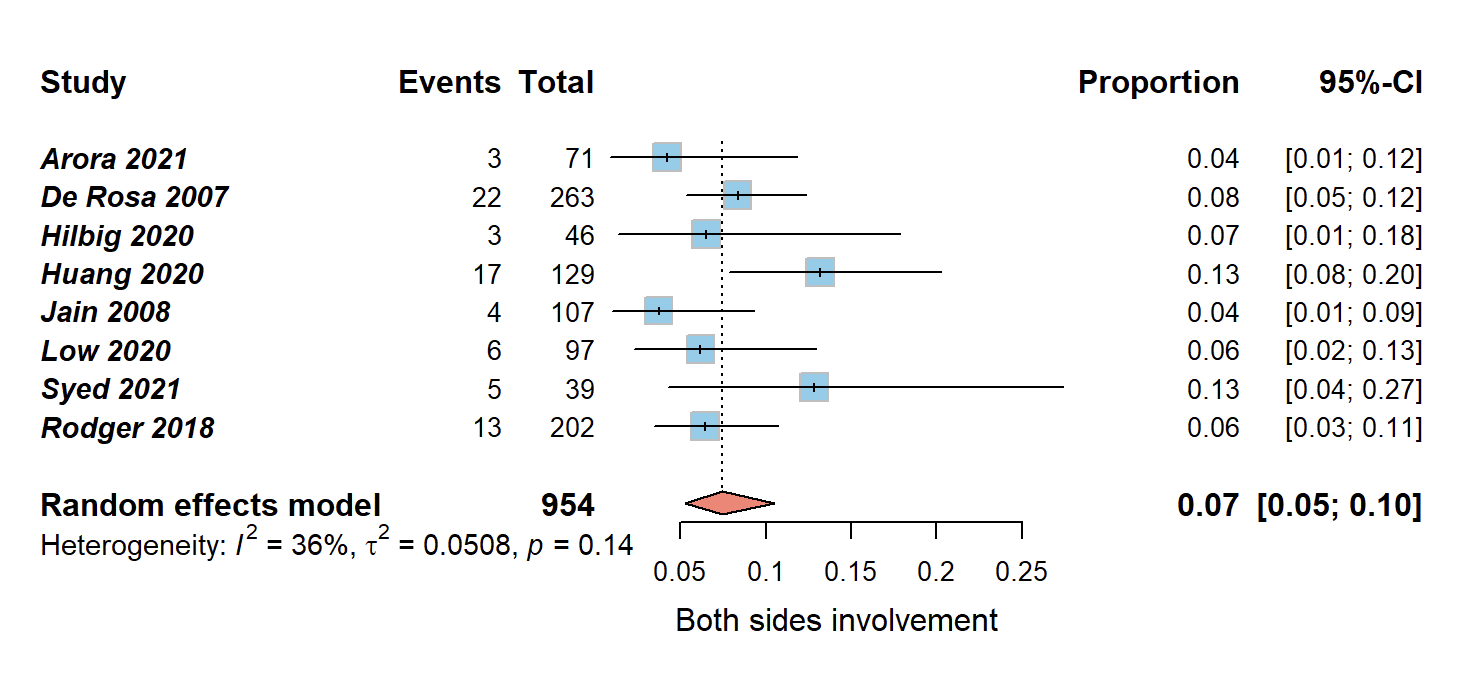
*

**Figure 74**. Forest plot: both-sided valve involvement

*
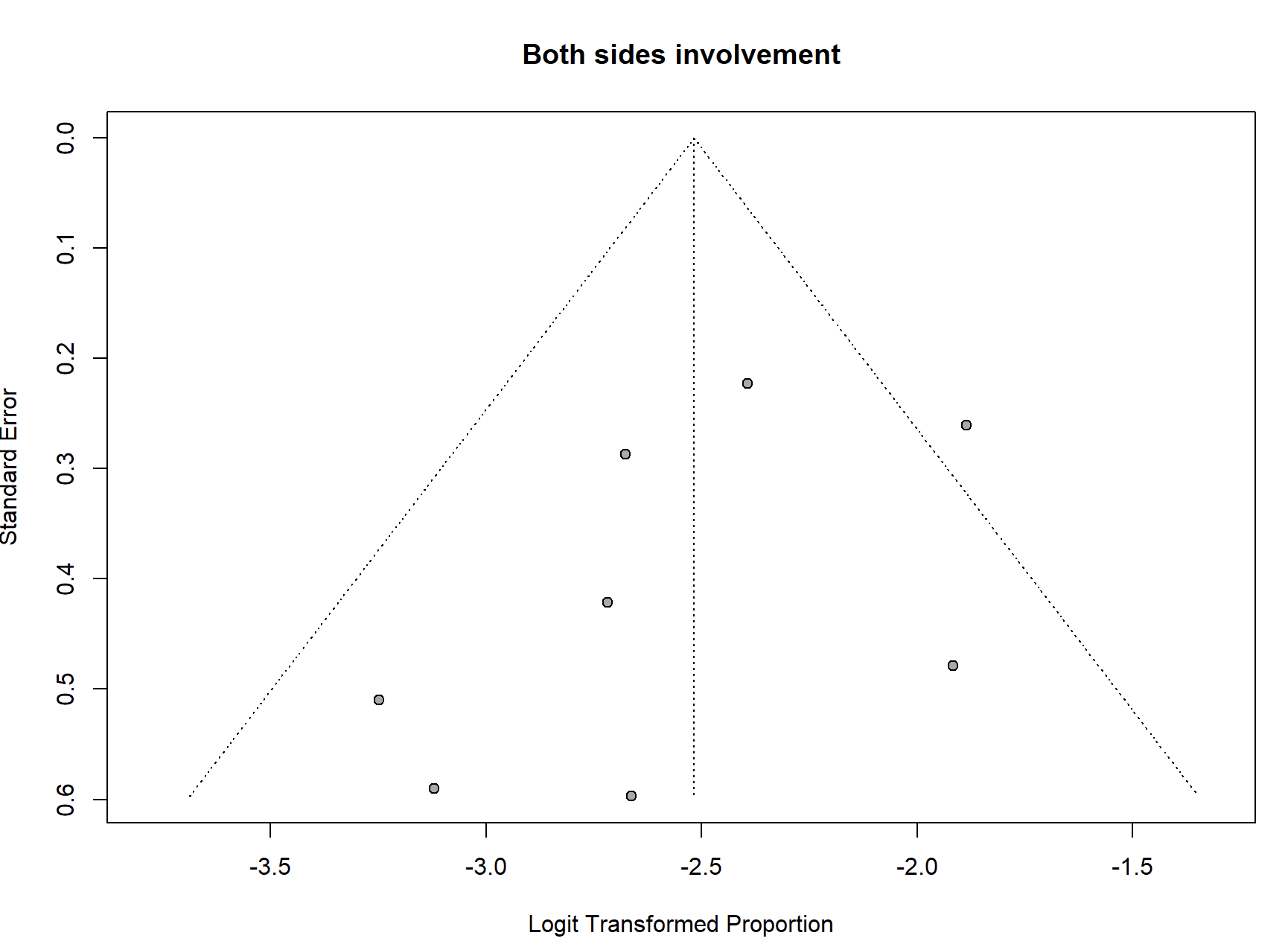
*

**Figure 75.** Funnel plot: both sides involvement
