# Supplementary material for: Valve involvement in infective endocarditis among intravenous drug users: a systematic review and meta-analysis
Source: BMC Infect Dis. 2026 May 12;26:1262. doi: 10.1186/s12879-026-13284-9 (PMC13343716; doi:10.1186/s12879-026-13284-9)
Supplement: Supplementary file 2 — Supplementary Material 2 [file 12879_2026_13284_MOESM2_ESM.docx]

**Pubmed:**

("infective endocarditis" OR "endocarditis") AND ("intravenous drug use" OR "IV drug use" OR "injection drug use" OR "substance abuse" OR IV) AND ("prevalence" OR "epidemiology" OR "incidence")

740 results

**Embase:**

('infective endocarditis'/exp OR 'endocarditis'/exp)

AND ('intravenous drug use'/exp OR 'injection drug use' OR 'substance abuse'/exp)

AND ('prevalence'/exp OR 'epidemiology'/exp OR 'incidence'/exp)

879 results

**Scopus:**

(TITLE-ABS-KEY("infective endocarditis" OR "endocarditis"))

AND (TITLE-ABS-KEY("intravenous drug use" OR "IV drug use" OR "injection drug use" OR "substance abuse" OR IV))

AND (TITLE-ABS-KEY("prevalence" OR "epidemiology" OR "incidence"))

692 results

**WoS:**

("infective endocarditis" OR "endocarditis") AND ("intravenous drug use" OR "IV drug use" OR "injection drug use" OR "substance abuse" OR IV) AND ("prevalence" OR "epidemiology" OR "incidence") (All Fields)

346 results
