# Supplementary material for: Valve involvement in infective endocarditis among intravenous drug users: a systematic review and meta-analysis
Source: BMC Infect Dis. 2026 May 12;26:1262. doi: 10.1186/s12879-026-13284-9 (PMC13343716; doi:10.1186/s12879-026-13284-9)
Supplement: Supplementary file 3 — Supplementary Material 3 [file 12879_2026_13284_MOESM3_ESM.docx]

**Framework and criteria (PECOST)**

To guide study selection and data extraction, the following PECOST (Population, Exposure, Comparison, Outcomes, Study design, Time) framework was used:

- Population (P): Hospitalized adults (≥18 years) diagnosed with infective endocarditis who are intravenous drug users (IVDUs). This includes patients where the diagnosis was confirmed using standard clinical criteria (e.g., the modified Duke criteria or the Duke-ISCVID criteria).
- Exposure (E): Recent recorded use of intravenous drugs, explicitly defined as documented and current use rather than merely a past history. Only patients with recent intravenous drug use and a corresponding diagnosis of definite IE (as defined by the Duke–ISCVID criteria) were included. Subgroup analyses explored differences among IVDU-IE patients who received varying treatments (e.g., surgery vs. medical therapy) and other study-specific subgroups.
- Comparison (C): Not applicable. This review focuses on characterizing the prevalence and clinical features of IE among IVDUs without a direct comparator group; however, some analyses compare prevalence estimates between right-sided and left-sided IE within the IVDU population.
- Outcomes (O):
  - *Primary outcome:* Pooled prevalence of left heart involvement (involvement of the mitral or aortic valve) in infective endocarditis among IVDUs, along with the prevalence of individual valves affected.
  - *Secondary outcomes:* Identification of bacterial agents involved; analysis of pre- and post-treatment complications; short- and long-term mortality; as well as rates of readmissions, re-operations, adherence to treatment protocols, and relapse rates.
- Study design (S): Eligible studies include randomized controlled trials (RCTs), prospective and retrospective cohort studies, case-control studies, and observational studies with comparative arms. Although various study designs were considered, all of the included studies were observational.
- Time (T): There were no restrictions related to the original publication date or language regarding study design; however, our electronic search was limited to the period January 2021 – December 2025 to capture the most recent evidence.

This structured PECOST framework was instrumental in guiding the inclusion criteria and ensuring that all extracted data accurately represented the intended study populations and outcomes.
