# Supplementary figures and images for: Valve involvement in infective endocarditis among intravenous drug users: a systematic review and meta-analysis
Source: BMC Infect Dis. 2026 May 12;26:1262. doi: 10.1186/s12879-026-13284-9 (PMC13343716; doi:10.1186/s12879-026-13284-9)

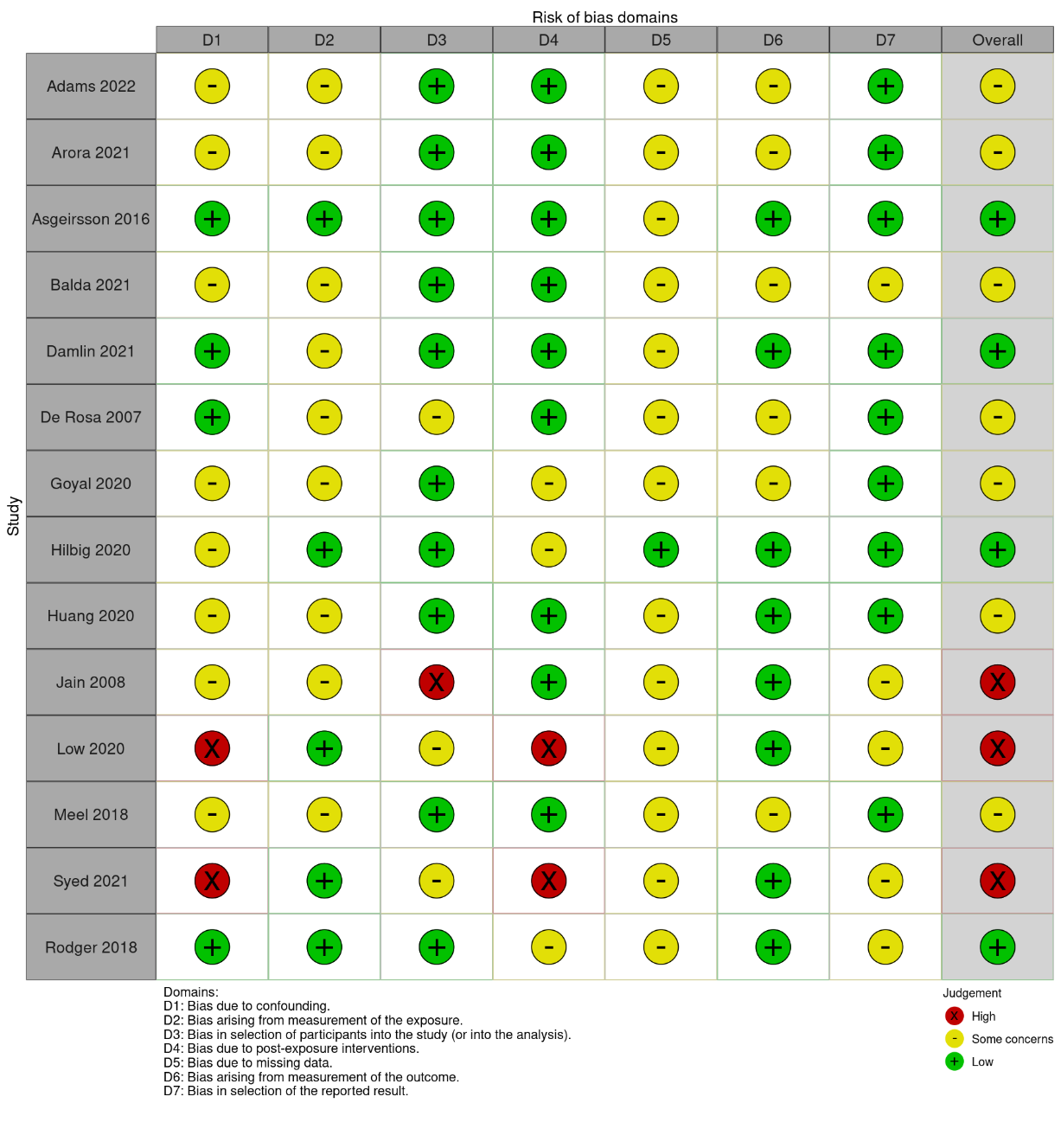


*
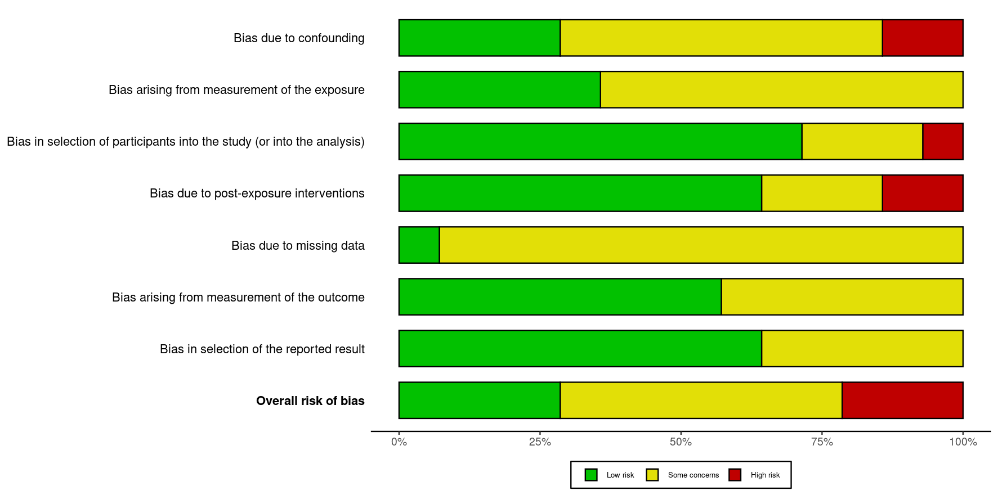
*

**Supplementary Figure 1.** Risk of bias assessment across included studies using the ROBINS-E tool

Supplement: Supplementary file 4 — Supplementary Material 4 [file 12879_2026_13284_MOESM4_ESM.docx]
